# Early evolutionary history of the seed

**DOI:** 10.1002/brv.70134

**Published:** 2026-01-26

**Authors:** Richard M. Bateman, Alan R. T. Spencer, Jason Hilton

**Affiliations:** ^1^ Jodrell Laboratory Royal Botanic Gardens Kew Richmond Surrey TW9 3DS UK; ^2^ Department of Earth Science and Engineering Imperial College London London SW7 2BP UK; ^3^ Science Group The Natural History Museum Cromwell Road London SW7 5BD UK; ^4^ School of Geography, Earth and Environmental Sciences, University of Birmingham Edgbaston Birmingham B15 2TT UK; ^5^ Birmingham Institute of Forest Research, University of Birmingham Edgbaston Birmingham B15 2TT UK

**Keywords:** anatomical preservation, apoptosis, evolution, gymnospermous reproduction, heterochrony, morphological cladistics, palaeobotany, Palaeozoic, phylogeny, reproductive biology, synorganisation, transference of function

## Abstract

The seed is an essential stage in the life history of gymnospermous and angiospermous plants, facilitating both their survival and dispersal. We reappraise knowledge of the evolutionary history of the gymnospermous seed, from its origin in the late Devonian through to the well‐known end‐Permian extinctions – an interval encompassing the origins of most major lineages of seed‐bearing plants. The framework for our broader discussions is a novel cladistic analysis of anatomically preserved Palaeozoic seeds, analysing 79 seed‐species for 89 morphological characters in a matrix containing only 24% missing values. The resulting consensus tree is weakly but fully resolved and compatible with traditional division into three informal seed groups: paraphyletic lagenocarps, paraphyletic trigonocarps and monophyletic cardiocarps. Three seed‐genera – *Rhychosperma*, *Albertlongia* and *Muricosperma* – are revealed as potential ‘missing links’ between groups, and modest re‐circumscription of seed‐genera is required. Although the value of single‐organ phylogenies remains controversial, the present seed‐tree topology receives general support from the dated sequence of first appearances of seed‐species in the fossil record, and from the topologies of morphological cladistic studies that combined conceptually reconstructed fossil plants with primitive extant lineages, notably ginkgos and cycads. Branch lengths in the tree and phenetic distances in ordinations of the matrix indicate similar overall rates of character change through the Palaeozoic, rather than a fractal pattern reflecting progressively increasing constraint, although early changes in architectural and pollination‐related characters gradually give way to greater experimentation with the internal layering and external topography of the testa. Our process‐based evolutionary inferences are informed by extant gymnosperms, particularly *Cycas* and *Ginkgo*. The origin of the true seed is attributed primarily to (1) the complex biochemical signalling needed to allow the sperm to reach the archegonia through the megasporangium wall and (2) the localised apoptosis of the megasporangium hypothesised to have simultaneously allowed hollowing out of the nucellar apex to form a sophisticated pollen‐receiving apparatus (the pollen chamber) and secretion of a pollination drop to capture air‐borne (pre)pollen. Subsequent potential key innovations include transfer of function of both pollination‐drop channelling and pollen chamber sealing from the nucellar salpinx to the integumentary micropyle, and introduction of a haustorial pollen tube to direct spermatozoa towards the archegonia. Assuming that the seed‐plant megasporangium terminates an axis, synorganisation has played a key role in seed evolution, leaf‐like lateral organs being repeatedly pulled towards the apex and incorporated into the terminal structure. Lateral webbing of integumentary lobes eventually almost fully enclosed the nucellus, while a similar synorganisation process affecting a lower set of vegetative organs formed a cupule as yet another protective layer surrounding one or more ovules. Our tree refutes viewing these evolutionary developmental trends as linear transition series. The earliest seeds were small but soon increased to reach the maximum size achievable by gymnosperms. Dehiscence and dormancy mechanisms were likely primitive at best, while increasingly complex layering and sculpting of the testa may have aided both abiotic and biotic dispersal. The end‐Permian extinction of plants bearing lagenocarps and trigonocarps is attributed tentatively to one or more of several features of reproductive biology identified as being vulnerable to desiccation.

## I. INTRODUCTION

### (1) Nature of the seed

Few evolutionary innovations have achieved as great an impact on the Earth's biosphere as the humble seed. A typical seed not only immediately and substantially provisions the next generation of plants but may also permit a period of dormancy during which it operates as a disseminule, potentially allowing colonisation by the species of distant (and, in some cases, previously unoccupied) terrains. In addition, energy‐rich seeds are a fundamental element in many food chains, not least our own.

The seed is best defined as a fertilised ovule, and an ovule is in turn most appropriately defined as an indehiscent integumented megasporangium. In order to comprehend this terminology it is necessary to compare seed‐plants with those potential ancestors among the wide diversity of heterosporous pteridophytic plants (‘ferns’ *s.l*.) that, unlike most pteridophytes, bear two kinds of sporangium: one produces large numbers of small ‘male’ microspores whereas the other, more derived category of sporangium produces larger, better‐resourced, ‘female’ megaspores (e.g. Bateman & DiMichele, 1994). In order for evolution to generate a true ovule/seed, the megaspores became retained within the megasporangium rather than being dehisced individually into the environment (thus ‘indehiscent’), three of the four meiotic products were aborted to leave only one functional megaspore, and the megasporangium wall (termed the nucellus in seed‐plants) then became largely encased within an outer protective layer of sterile tissue termed the integument (thus ‘integumented’). Clearly, the pteridophyte megaspore is homologous with the inner (gametophytic) regions of the seed, whereas the pteridophyte microspore is homologous with the pre‐pollen/pollen that is produced by seed‐bearing plants in order to generate and disseminate the ‘male’ gametes needed to fertilise the ovule and thereby generate a viable seed. Acquisition of the seed‐habit famously liberated gymnosperms from dependency on free water for sperm dispersal (e.g. Chaloner, 1967; Chaloner & Pettitt, 1987; Rothwell & Scheckler, 1988; Bateman & DiMichele, 1994).

**Fig. 1:**
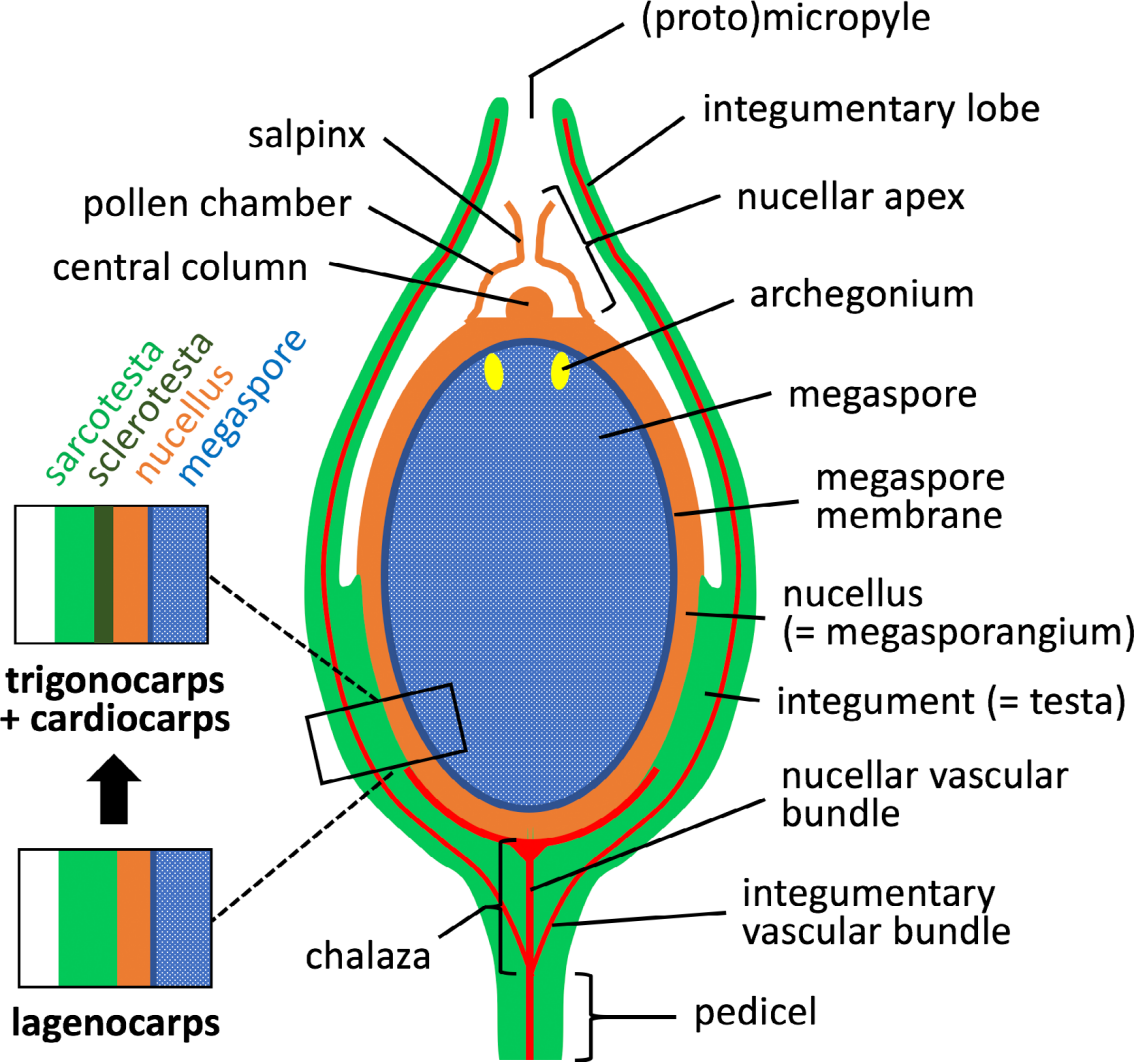
Diagram of typical Palaeozoic lagenocarp seed, including comparison of the three‐layered wall structure of lagenocarps with the four‐layered trigonocarps and cardiocarps. Redrawn and modified after Long (1960a) and Hilton & Bateman (2006) (which see for greater terminological detail).

The fundamental stage in the life history of any seed‐plant that is represented by the seed requires completion of several key roles, each one crucial to successful reproduction. The distal region of the ovule is responsible for pollination and fertilisation, whereas the more vascularised proximal region resources the developing ovule, which must nurture first the megagametophyte and then the embryo (e.g. Rudall, 2021). Throughout its development, the increasingly energy‐rich ovule requires protection from both pathogens and herbivory (Labandeira, Kvacek & Mostovski, 2007; DiMichele *et al*., 2023), and once the seed reaches maturity, both physiological and morphological aids to dispersal come into play. Multiple layers, and local specialisation of function within those layers, provided the obvious foundation to any evolutionary solution to these challenges. Nonetheless, such intense multi‐tasking, critical to the survival of the lineage, inevitably demands functional trade‐offs, given that all of these roles must be completed within a limited repertoire of tissues and a limited volume of space. It is the three‐dimensional arrangement of those tissues (summarised in Fig. 1) that underpins the present study.

### (2) Fossil record of seeds

Given current evidence, the fossil record indicates that seed‐bearing plants (spermatophytes: gymnosperms plus angiosperms) first appeared in the Famennian stage of the Late Devonian [*c*. 365 million years ago (Ma); Rothwell, Scheckler & Gillespie, 1989]. Admittedly, recent attempts to extrapolate the origin of spermatophytes from molecular clocks based on extant taxa collectively offer a much wider range of options, stretching from 388 to 454 Ma (Clarke, Warnock & Donoghue, 2011) and 386–451 Ma (e.g. Barba‐Montoya *et al*., 2018) through 377–421 (Ran *et al*., 2018) to a more youthful 330–365 Ma (e.g. Morris *et al*., 2018). Although some pre‐cladistic reviews envisaged at least two independent evolutionary origins for seed‐bearing plants (*cf*. Arnold, 1948; Beck, 1960, 1966, 1970, 1976, 1981; Chaloner, Hill & Lacey, 1977; Stewart, 1981; Meyen, 1984, 1987; Stein & Beck, 1987), subsequent cladistic studies rooted in explicit data analysis have consistently indicated a single origin of the seed *sensu stricto*, irrespective of whether the resulting phylogenies were derived from matrices based on morphological data (e.g. Doyle & Donoghue, 1986; Hilton & Bateman, 2006; Rothwell & Stockey, 2016; Coiro, Chomicki & Doyle, 2018) or molecular data (e.g. Burleigh & Mathews, 2004; Graham & Iles, 2009; Ran *et al*., 2018; Yang *et al*., 2022). Spermatophytes are thus strongly supported as monophyletic – an assumption that underlies comments made throughout the present study.

Today, the most evolutionarily and ecologically successful group of seed‐bearing plants throughout all but the coldest regions of the globe are the angiosperms, commonly (if somewhat simplistically) termed the flowering plants. The phylogenetic position of the angiosperm lineage remains contentious; its origin is located surprisingly deep in time if we choose to believe the majority of the many molecular phylogenies based exclusively on extant taxa (e.g. Barba‐Montoya *et al*., 2018; Li *et al*., 2019b). Nonetheless, plants that meet modern circumscriptions of angiosperms constitute an evolutionarily derived clade that, according to the fossil record, has dominated most of the Earth's biomes only during the last *c*. 85 million years (Myr). During the previous *c*. 290 Myr of land‐plant evolution, seed‐plants have instead been represented by arguably 12 major lineages of gymnosperms, of which only four – cycads, *Gingko*, conifers (coniferaleans plus pinaleans) and gnetaleans – have survived to each provide us with at least one representative extant species (e.g. Bateman, Hilton & Rudall, 2006). Of these 12 lineages, 10 were already unequivocally represented in the fossil record by the end of the Palaeozoic Era (dated to 252 Ma), excepting only the extinct Pentoxylales and the extant angiosperms. Thus, the early evolutionary history of the seed is inevitably a story told primarily through study of the fossil record during the Devonian, Carboniferous and Permian periods, which together constitute the Late Palaeozoic Era. It is these pioneering seeds that provide the framework for the present account.

### (3) Organ‐phylogenies *versus* whole‐plant phylogenies

Through the last four decades, morphological phylogenetic analyses have played a crucial role in integrating the extant and extinct major lineages of gymnosperms, and in inferring their evolutionary relationships (e.g. Crane, 1985, 1985, 1990; Doyle & Donoghue, 1986; Nixon *et al*., 1994; Rothwell & Serbet, 1994; Hilton & Bateman, 2006). However, extinct taxa included in these analyses have of necessity been based on a disappointingly small number of whole‐plant concepts, each painstakingly reconstructed from up to a dozen component fossil organs into which members of that species almost inevitably disarticulated *post mortem* (Bateman & Hilton, 2009). Employed in cladistic analyses as typological representatives of lineages that were once far more diverse, credible whole‐plant reconstructions of seed‐plants are so few in number that the same familiar archetypal whole‐plant species have, from Crane (1985a) and Doyle & Donoghue (1986) onward, consistently been recycled from one comparative analysis to the next, typically with modest changes made to the characters scored. The ongoing limited supply of reconstructed fossils severely restricts the range of relationships among taxa that can potentially be inferred through matrix‐based analyses.

Given the continued rarity of conceptually reconstructed whole‐plants (Bateman & Hilton, 2009), the only practical approach to exploring a much wider spectrum of that crucial palaeo‐biodiversity is to relax our self‐imposed constraints on morphological analyses and instead attempt to compare isolated organs. Most commonly, an organ‐species of particular interest is placed within a matrix otherwise constructed from conceptual whole‐plants. In some cases, the resolution of the original topology survives and the organ‐species is convincingly placed (e.g. Bateman, Kenrick & Rothwell, 2007; Stevens *et al*., 2010), whereas in other, less successful, cases the topology is destabilised by the myriad missing values that by definition represent the bulk of any data set derived from an organ‐species (e.g. Friis *et al*., 2007; Rothwell, Crepet & Stockey, 2009). However, the present analysis adopts an even bolder strategy, by comparing only different organ‐species within a single selected category of organ.

Any category of organ chosen as a proxy for conceptual whole‐plant fossils of seed‐plants should fulfil three essential requirements: it should be: (*i*) frequently preserved in the fossil record; (*ii*) rich in morphological/anatomical characters; and, through the agency of these characters, (*iii*) have been demonstrated *via* previous reconstructions of whole‐plant phylogenies to provide a comparatively strong phylogenetic signal (Bateman & Simpson, 1998). It is also highly desirable that the chosen category of organ should fulfil biological functions that are both unambiguous and crucial to the well‐being of the lineage. Ideally, the organ should in addition mix characters that are highly conserved and so improve the robustness of the resulting trees with other characters that are more readily modified through evolution and so improve their taxonomic resolution. Here, we argue that fossil seeds offer the most effective and informative solution to this long‐term conundrum, at least when considering early seed‐plants of Late Palaeozoic age. A similar conclusion was reached by Hilton & Cleal (2007) following their biogeographical analysis of Palaeozoic seed plants. Seeds are character rich and, when combined with (pre)pollen, represent an entire stage in the life history of all Palaeozoic seed‐plants.

**Fig. 2:**
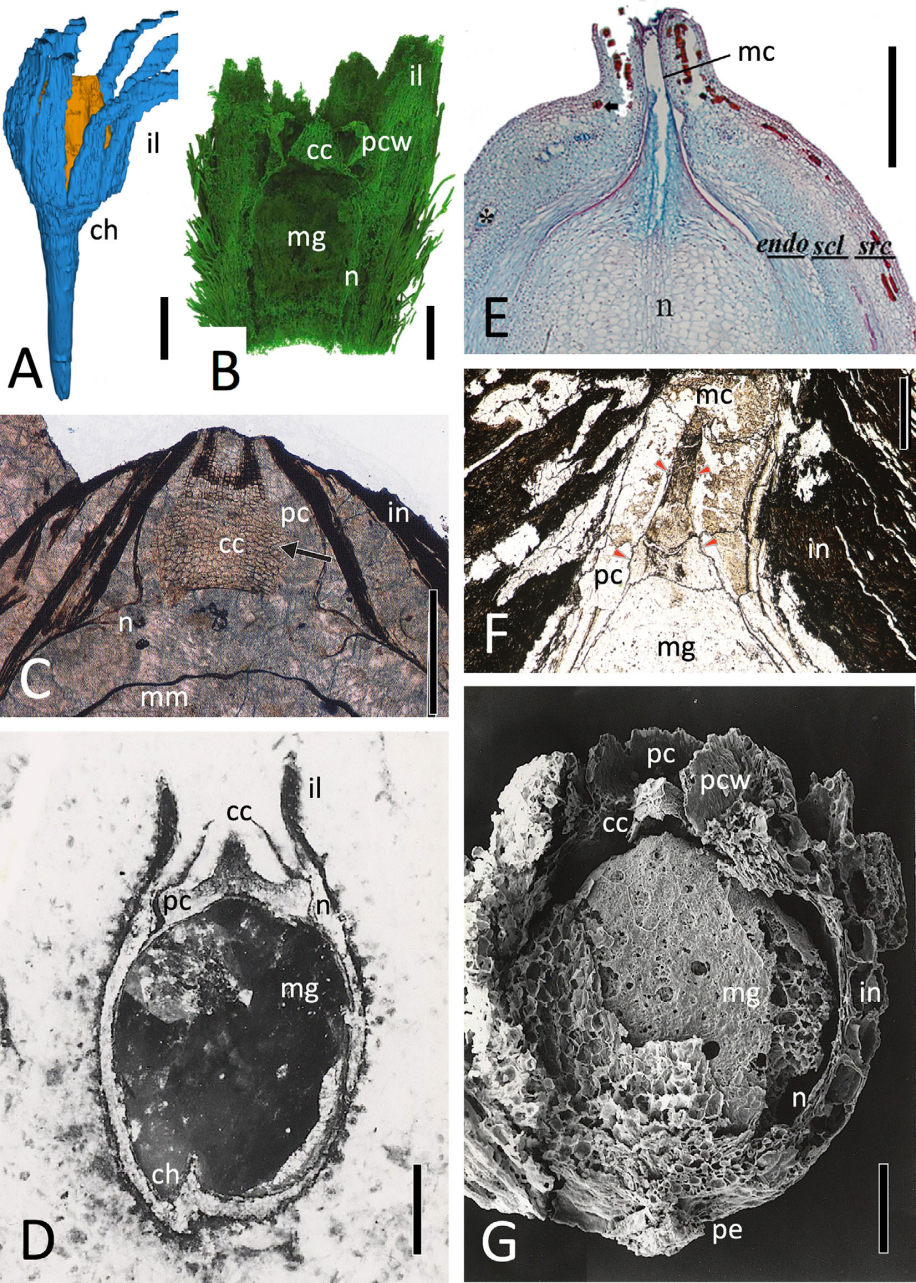
Contrasting methods of obtaining anatomical detail from fossil seeds, as viewed through near‐median longitudinal sections. (A) Computerised reconstruction from serial acetate peels of seeds of *Genomosperma kidstonii*, a lagenocarp from the Tournaisian of NE England. Orange tissues are the nucellus, blue tissues the integument. (B) X‐ray microtomographic reconstruction of *Hirsutisperma rothwellii*, a lagenocarp from the Visean of SE Scotland. (C) Petrological thin‐section of distal region of *Lagenostoma lomaxii* (the ovule that inspired the concept of pteridosperms/seed‐ferns), a lagenocarp from the Bashkirian of N England; arrow indicates central column. (D) Acetate peel of *Stamnostoma oliveri*, a lagenocarp from the Tournaisian of SE Scotland. (E) Anatomical section of distal region of *Zamia furfuracea*, an extant cycad seed from Mexico possessing a cardiocarp‐style anatomy; image included for comparison with F. (F) Acetate peel of *Traskia maahle*, a fossil cycad seed from the Callovian (Jurassic) of W Canada; arrows indicate the apex of the pollen chamber wall. (G) Scanning electron micrograph of a partially dissected charcoalified specimen of *Deltasperma fouldenense*, a lagenocarp from the Tournaisian of SE Scotland. Labels: cc = central column; ch = chalaza; endo = endotesta; in = integument; il = integumentary lobes; mc = micropylar canal; mg = megaspore; mm = megaspore membrane; n = nucellus; pc = pollen chamber; pcw = pollen chamber wall; pe = pedicel; scl = sclerotesta; src = sarcotesta. Scale bars: 0.2 mm (B, E); 0.5 mm (C, D, G); 1 mm (F); 2 mm (A). Sources: (A) Meade *et al*. (2021, fig. 1a); (B) Scott *et al*. (2019, fig. 6h); (C) Taylor *et al*. (2009, fig. 14.34); (D) Bateman & Rothwell (1990, fig. 1g); (E) Zumajo‐Cardona *et al*. (2021, fig. 7E); (F) Rothwell *et al*. (2022, fig. 5e); (G) Bateman & Rothwell (1990, fig. 9b). Reproduced through pemission of the Licensor through CopyrightClearanceCenter.

Although seeds are frequently found in the fossil record, usually isolated from the remainder of the source plant, the great majority are preserved only as two‐dimensional adpressions – a mode of preservation that greatly restricts the range of morphological characters for which the seeds can be scored when developing a numerical matrix. We have therefore confined the present comparative analysis to unusually well‐preserved permineralised ovules. Such fossils are not only three‐dimensional but also retain anatomical preservation, in the form of cellular details that today are typically teased out of fossils through a combination of non‐destructive imaging and destructive serial sectioning (e.g. Friis *et al*., 2007; Spencer, Hilton & Sutton, 2013a; Meade, Plackett & Hilton, 2021). At their best, such fossils yield microscopic detail comparable with that of any living seed. Figure 2 shows examples selected to summarise both the range of preservation available and the various techniques available to extract data from such material.

### (4) Issues addressed

When combined with observations on extant taxa, the novel seed phylogeny generated through the present study provides us with the framework needed to address a broad spectrum of high‐level methodological, evolutionary and ecological questions. The accumulation of successive synapomorphies necessary to develop the combination of characters that defines the seed habit have been explored in numerous previous studies (e.g. Chaloner & Pettitt, 1987; Rothwell & Scheckler, 1988; Bateman & DiMichele, 1994), whereas here we primarily consider events that occurred through the subsequent 110 Myr of seed‐plant evolution. We ask how closely the relationships evident in our taxonomically rich seed phylogeny match those suggested by inevitably more sparsely sampled, but more character‐rich, phylogenies based on the corresponding conceptually reconstructed whole‐plants, and also how they compare with competing traditional taxonomies. We determine which character states of the seed‐species are primarily responsible for their phylogenetic placements, seeking both cohesive groups and potential ‘missing links’ that help us to infer overall evolutionary trends. We then explore possible key innovations and evolutionary processes underlying these trends, exploiting inferences relating to functional morphology that draw on observations of both extinct and extant seed‐plants. Finally, we attempt to determine the sequence and approximate dates of origin of several putatively key evolutionary innovations, and search for credible correlations with other significant events documented in the geological record of the Palaeozoic Era.

We should state at the outset that, while acknowledging that mosaic evolution is widespread and often strongly expressed among higher plants (e.g. Stebbins, 1984), we nonetheless recognise that individual categories of organ such as ovules do not evolve *per se* – they can only legitimately be viewed as indicators of the wider range of heritable changes undergone by their ‘host’ plants.

## II. METHODS

### (1) Matrix compilation

The present morphological matrix of seed‐species was built on foundations laid by Seyfullah *et al*. (2010), whose matrix encompassed 45 species of Palaeozoic ovules scored for 43 characters. This in turn owed much to the earlier matrix of Hilton, Wang & Tian (2003), which contained 27 fossil seed‐species scored for 41 characters. Here, an intensive literature search, combined with a wholesale reappraisal of previous definitions of characters and their component states, allowed us to score initially 81 Palaeozoic seed‐species for 91 morphological characters, although two seed‐species (see online Supporting Information, Appendix S1 for list of included seed‐species) and two characters (Appendix S2) were abandoned prior to analysis. The remaining 89 characters represented a total of 218 character states. The resulting matrix totalled 7031 cells (see Data_S1). It contained 13.9% of cells scored as ‘unknown’ and a further 10.5% of cells scored as ‘inapplicable’, together constituting 24% missing values (a modest proportion when viewed in the context of a matrix consisting wholly of fossil taxa). Moreover, only a further 0.1% of cells were scored as polymorphic. The least complete character, containing 89% missing values, was ‘Overtopping amongst ovules within cupule’ (C78). Among the scored taxa, *Stephanospermum truncatum* yielded (by a considerable margin) the least complete data set, containing 48% missing values, whereas *Genomosperma kidstonii* was most complete, incurring only 4% missing values. Three additional columns concluding the matrix represented extrinsic properties and hence were employed only for *post‐hoc* mapping: permineralising medium, depositional environment, and phytogeographic realm.

### (2) Data analysis

Our matrix was compiled in Nexus format in Mesquite v3.8 (Maddison & Maddison, 2023), and analysed using TNT v1.6 (Goloboff, Farris & Nixon, 2008; Goloboff & Morales, 2023). The oldest scored taxon – the late Devonian *Elkinsia polymorpha* – was identified as the single outgroup, based on its reliable placement as the earliest‐diverging genus in previously published morphological phylogenies that included substantial numbers of both gymnosperms and their evolutionary precursors – the pteridophytes in general and progymnosperms in particular (Rothwell & Serbet, 1994; Hilton & Bateman, 2006; Rothwell & Stockey, 2016). The decision to treat only a single taxon as outgroup also meant that, in practice, seed‐plant monophyly was assumed *a priori*. Seven of the 89 characters (7.9%) included in the matrix proved to be phylogenetically uninformative. Trees were constructed only through parsimony, using ‘Traditional Search’. We ignored the entire array of increasingly popular likelihood and Bayesian tree‐building methods, reflecting our hard‐wired philosophy that no reasonable argument can be put forward to support any prior assumptions regarding how evolution might proceed phenotypically (as opposed to genotypically, where several supposed justifications have in practice caused complex modelling to become obligatory in studies employing nucleic acid sequence data).

Tree‐building involved 1,000 replications, each holding 100 parsimony trees, with random starting seed = 1 and collapse of zero‐length branches under ‘rule 3’; branch swapping employed tree bisection–reconnection. Branch lengths plus Bremer support and bootstrap support values were calculated for sets of individual trees, from which were generated majority‐rule and strict consensus trees. Once imported into Mesquite, the strict consensus tree provided the essential framework for mapping the state changes undergone by each individual character. It also provided the framework for a time‐scaled version of the tree, based on first and last appearances of each seed‐species in the fossil record, that was generated using the *geoscalePhylo()* function from the ‘strap’ R package (Bell & Lloyd, 2015) run under RStudio (R Core Team, 2023).

In order to assess morphological disparity, we also subjected the cladistic matrix of 79 taxa × 89 characters to principal coordinates analysis *via* a Gower Distance matrix (Gower, 1971). It was first necessary to re‐score polymorphic states as unknown (‘?’). Using an R script running under RStudio (R Core Team, 2023), the simplified matrix was read into R using the *read.nexus.data()* function from the ‘ape’ R package (Paradis & Schliep, 2019). Unknown (‘?’) and gap (‘–’) characters, totalling 24% of the matrix, were then transformed into N/A values, as they would otherwise have been treated as unique character states by the subsequent Gower algorithm. The taxa in the original matrix were re‐ordered to follow the base‐to‐apex sequence evident in the previously produced strict consensus tree. The Gower Distance matrix was then calculated using the *daisy()* function of the ‘cluster’ R package (Maechler *et al*., 2022) and used in turn to generate Principal Coordinates Analyses (PCoA; Gower, 1966) by combining the *pcoa()* function of the ‘ape’ R package with the core R *plot()* function. Finally, we employed the *fviz_dist()* function of the ‘factoextra’ R package (Kassambara & Mundt, 2020) to produce a heatmap, aiming to visualise better the matrix of Gower similarities among taxa.

## III. TOPOLOGY OF OUR RECONSTRUCTED SEED PHYLOGENY

### (1) Overview of our consensus tree

**Fig. 3:**
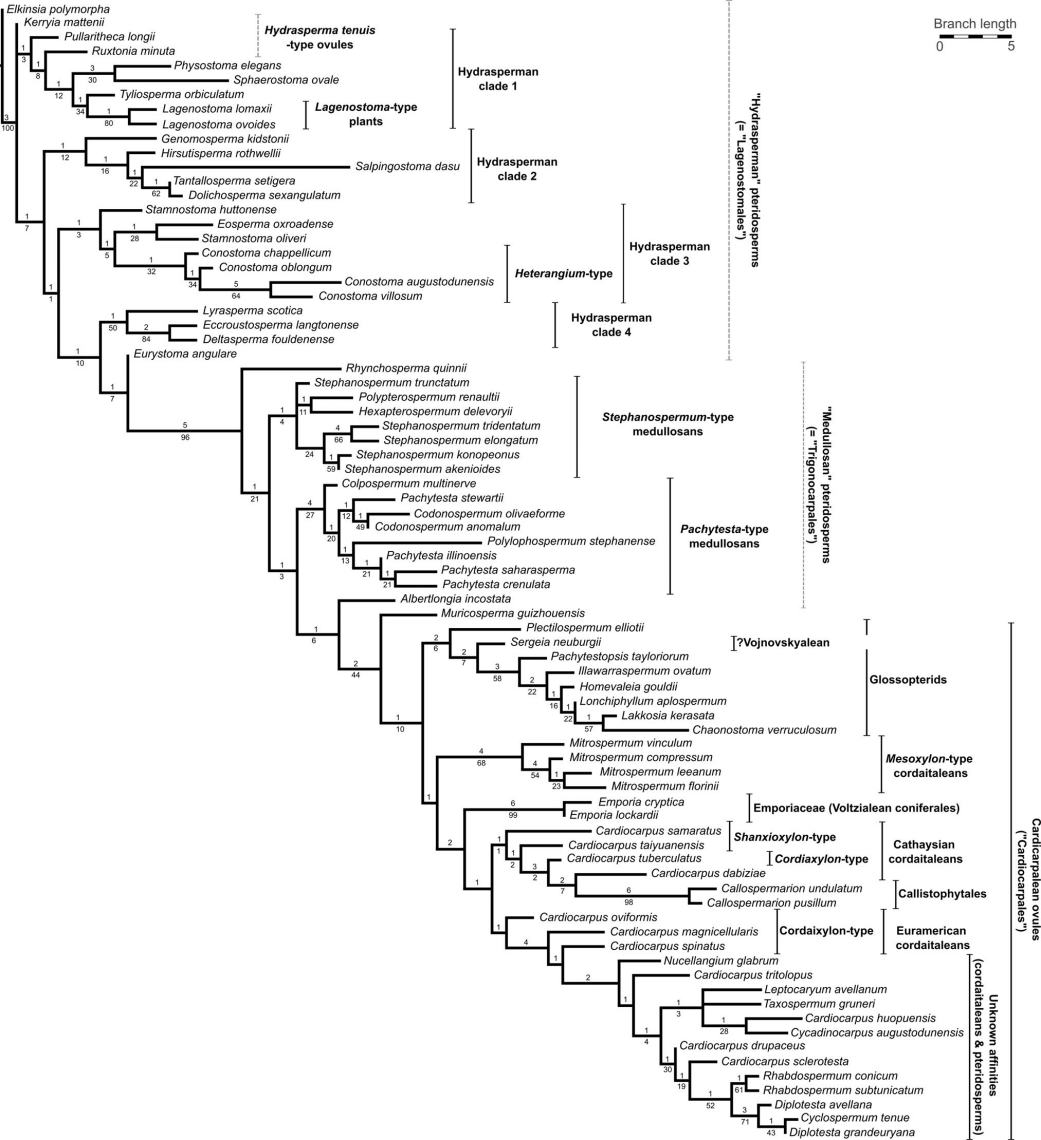
Strict consensus tree summarising 18 most‐parsimonious trees for 79 Palaeozoic seed‐species based on 89 morphological characters. Branches are proportional in length, bearing Bremer support values above and bootstrap values below. Taxonomic attributions relate to whole‐plant concepts of higher taxa.

Our pruned matrix of 79 seed species × 89 characters yielded 18 most‐parsimonious trees, which are summarised as a strict consensus tree in Fig. 3. Although the tree might initially appear well resolved, any neontologist familiar with statistical support values for genomics data is likely to be perturbed by its weak vital statistics. The ensemble retention index (0.726) and especially the consistency index (0.300) ostensibly indicate worryingly high levels of homoplasy. Only three of the 74 surviving internal branches attract more than 80% bootstrap support (and two of these are penultimate branches that merely link pairs of seed‐species already regarded as closely related). Moreover, given that the majority of the internal branches are only between one and three steps long, most yield a Bremer Support value of only 1.

**Fig. 4:**
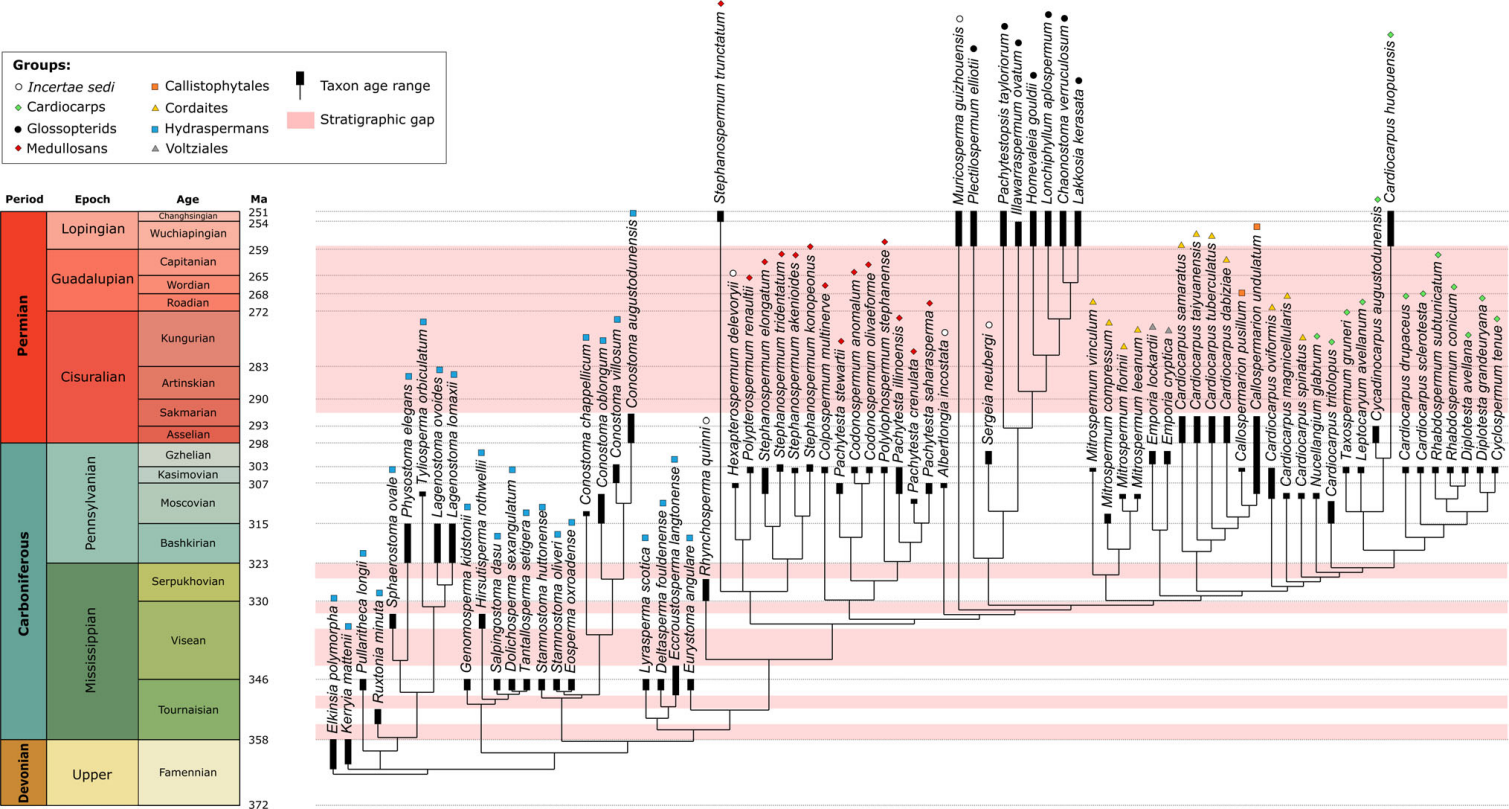
Strict consensus tree of 79 Palaeozoic seed‐species from Fig. 3 plotted against their geological age ranges. Time periods that have so far failed to yield anatomically preserved seeds are shown in pink.

**Fig. 5:**
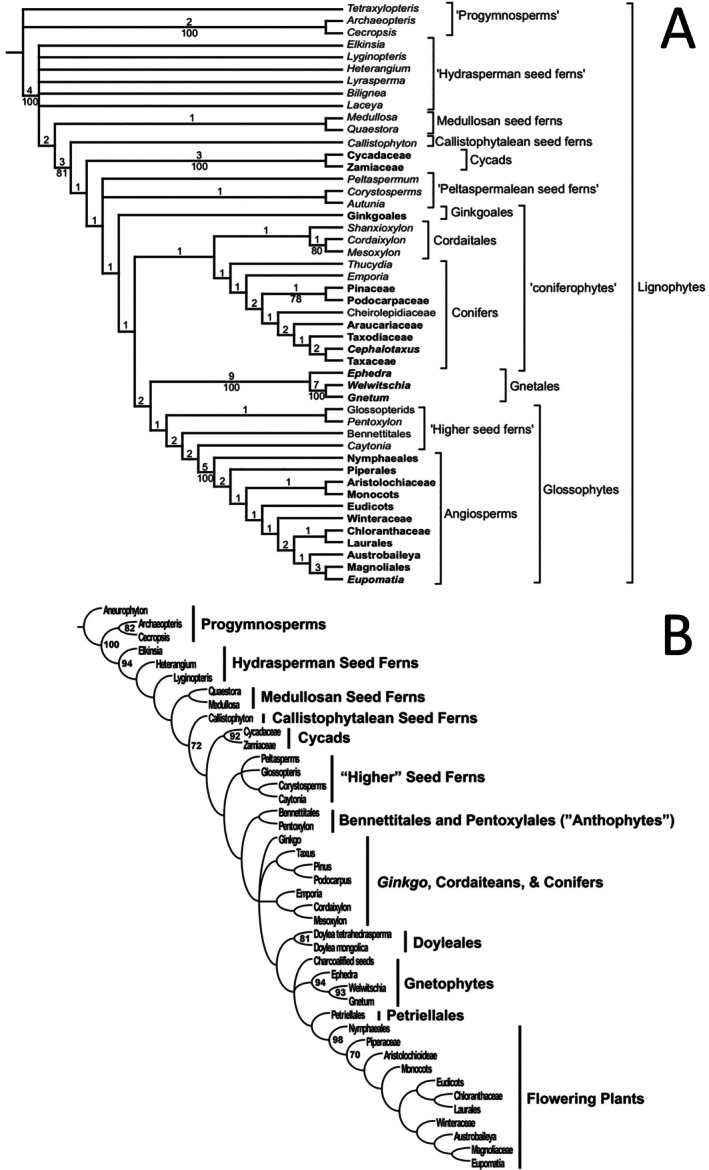
Two contrasting whole‐plant phylogenies that infer the relationships between major groups of extant and extinct seed‐plants. (A) Strict consensus of 21 most‐parsimonious trees for 48 coded taxa, showing Bremer support values above branches and bootstrap values greater than 50% below. Higher taxa with extant members are shown in bold. Figure 4 of Hilton & Bateman (2006). (B) Strict consensus of 12 most‐parsimonious trees for 42 coded taxa, showing bootstrap values greater than 70%. Fig. 28 of Rothwell & Stockey (2016). Reproduced through pemission of the Licensor through CopyrightClearanceCenter.

However, taking into account the inevitably low ratio of coded taxa to phylogenetically informative characters included in the analysis (1.04), and the presence of 24% cells in the matrix that are scored unknown or inapplicable, the resulting weak statistical support is unsurprising. Encouragingly, the recovery from such a taxon‐rich matrix of just 18 most‐parsimonious trees suggests the presence of meaningful structure in the data. Moreover, the stronger tests of the credibility of our topology involve various kinds of congruence: congruence with previous taxonomic assignments of the chosen seed‐species (Appendix S1), congruence with relative first appearances in the fossil record of the scored taxa (Fig. 4), and congruence with previous topologies based on morphological matrices for conceptual whole‐plants. The latter are richer in characters but are obliged to rely on just a few representative ‘whole‐plant’ taxa that have been conceptually reconstructed and act as *de facto* ‘placeholders’ for groups that formerly were far more biodiverse (Fig. 5). We will return to consider these extrinsic tests of credibility after first exploring the broad structure and taxonomic implications of the topology shown in Fig. 3.

### (2) Taxonomic assignment

#### (a) Ongoing relevance of the three groups of Seward (1917)

**Fig. 6:**
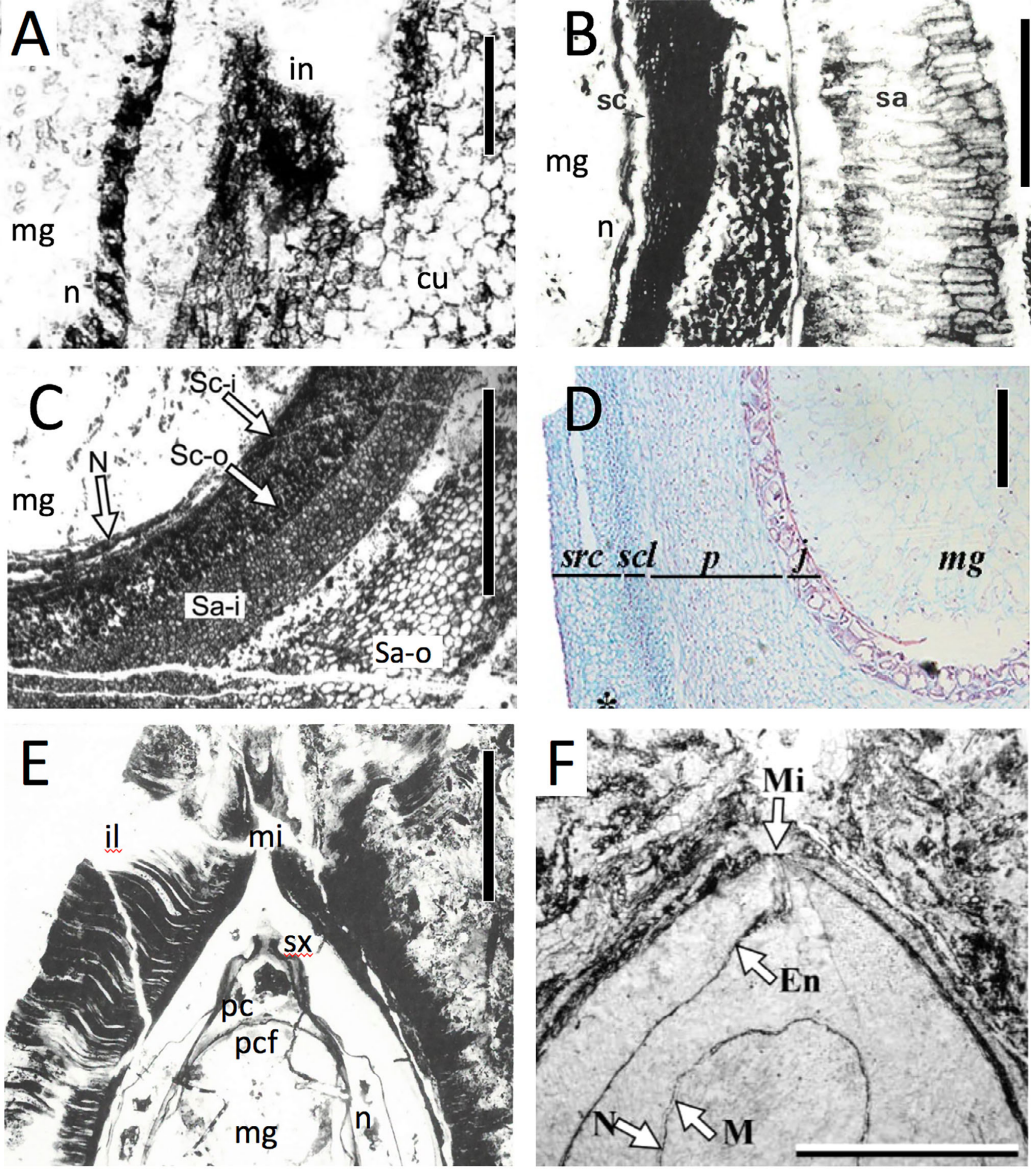
Comparative wall structure of ovules (A–D) and pollen‐receiving apparatus (E, F). (A) Relatively simple wall structure of the lagenocarp *Genomosperma kidstonii* from the Tournaisian of NE England, showing the poorly differentiated integument surrounded by a loose sparsely multi‐ovulate cupule. (B) Wall of the trigonocarp *Codonospermum anomalum* from the Kasimovian of SC France, showing the differentiation of the integument into a hard inner sclerotesta and fleshy outer sarcotesta. (C) Wall structure of the cardiocarp *Muricosperma guizhouensis* from the Wuchiapingian of SW China, showing the classic division of the integument into a dense sclerotesta enclosed by a less‐dense sarcotesta, each of which is further subdivided. (D) Ovule wall of the extant cycad *Zamia furfuracea*, illustrating the intimately fused layers and the difficulty of obtaining definitive homologies with seed‐ferns. (E) Longitudinal section of distal portion of the trigonocarp *Polypterospermum renaultii* from the Kasimovian of SC France, illustrating the reduced pollen chamber and functional micropyle. (F) Longitudinal section of distal portion of the cardiocarp *Cardiocarpus taiyuanense* from the Asselian–Sakmarian of NE China, showing further reduction of the pollen‐receiving apparatus. Labels: cu = cupule; En = endocarp; il = integumentary lobe; in = integument; j = jacket; Mi/mi = micropyle; M = megaspore membrane; mg = megaspore; N/n = nucellus; p = pachychalaza; pc = pollen chamber; pcf = pollen chamber floor; Sa/sa/src = sarcotesta (−i = inner, −o = outer); Sc/sc/scl = sclerotesta (−i = inner, −o = outer); sx = salpinx. Scale bars: (D) = 0.1 mm; (A, C) = 0.2 mm; (B) = 0.5 mm; (E, F) = 1 mm. Sources: (A) Meade *et al*. (2021, fig. 3b); (B, E) Combourieu & Galtier (1985, figs. 4.7, 1.2: www.schweizerbart.de/journals/palb); (C) Seyfullah *et al*. (2010, fig. 8); (D) Zumajo‐Cardona *et al*. (2021, fig. 7f); (F) Hilton *et al*. (2003, fig. 5).

**Fig. 7:**
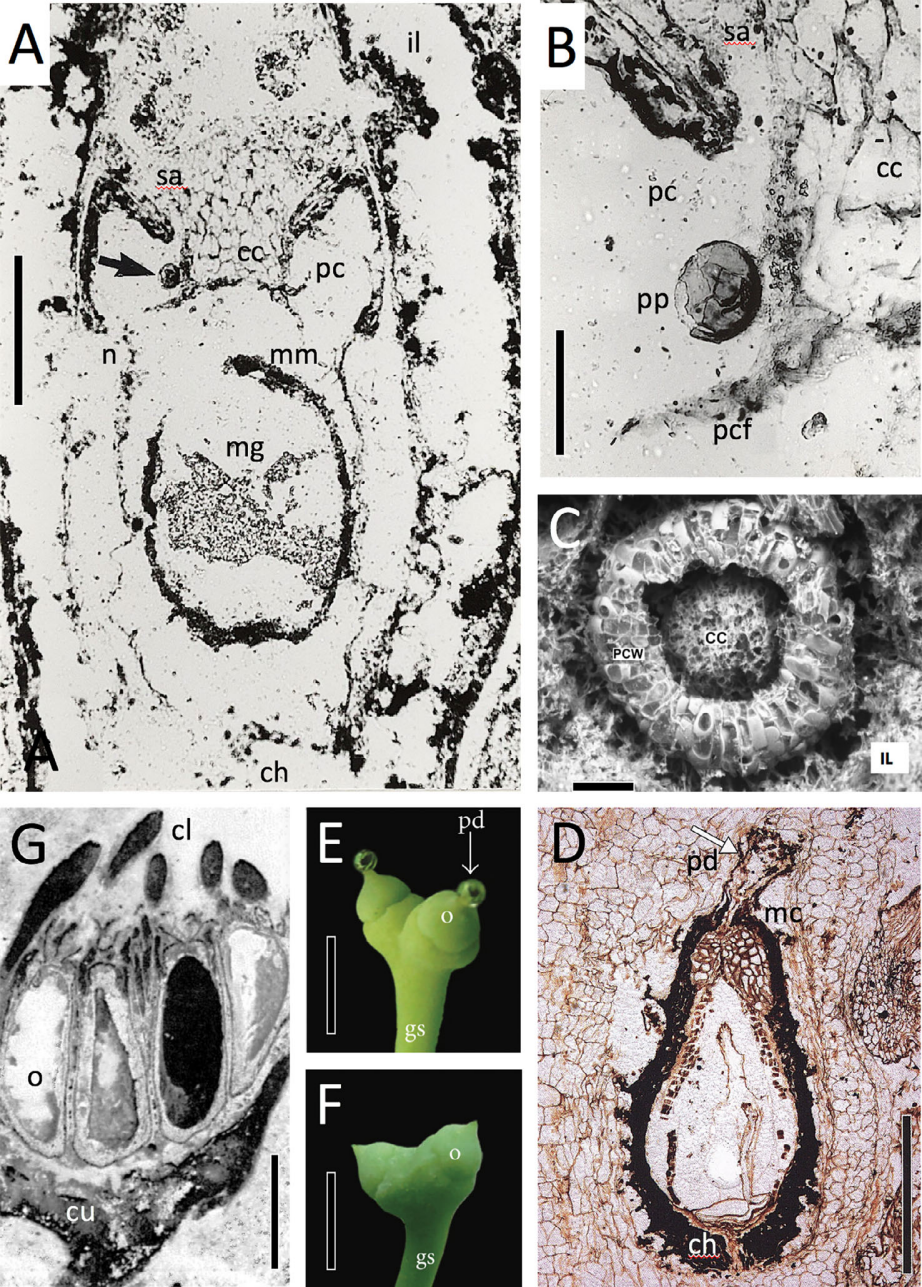
Pollination in early seed‐plants. (A) Median longitudinal section of *Sphaerostoma* sp., a lagenocarp from the Tournaisian of NE England, showing prepollen grain trapped within the pollen chamber (arrowed), which has subsequently been sealed through upward movement of the central column. (B) Enlargement of top‐left region of A. (C) Apical view of the apex of the charcoalified type specimen of *Hirsutisperma rothwellii*, a lagenocarp from the Visean of SE Scotland, showing features of the pollen chamber. (D) Longitudinal section of *Callospermarion pusillum*, a cardiocarp from the Kazimovian of Illinois, showing a fossilised pollination drop exuded from the micropylar canal and bearing pollen on the meniscus. (E, F) Extant primitive gymnosperm and *de facto* cardiocarp *Ginkgo biloba*, showing exudation and subsequent post‐pollination withdrawal of pollination drop. (G) Longitudinal section of a *Pullaritheca longii* cupule packed with mature *Hydrasperma tenuis* ovules, a lagenocarp from the Tournaisian of SE Scotland. Labels: cc = central column; ch = chalaza; cl = cupule lobe; cu = cupule; gs = “general (shared) stalk”; il/IL = integumentary lobes; mc = micropylar canal; mg = megaspore; mm = megaspore membrane; n = nucellus; o = ovule; pc = pollen chamber; pcf = pollen chamber floor; PCW = pollen chamber wall; pd = pollination drop; pp = prepollen grain; sa = salpinx. Scale bars: 0.1 mm (C); 0.2 mm (B); 1 mm (A, D); 2 mm (G); 10 mm (E, F). Sources: (A) Bateman & Rothwell (1990, fig. 4d); (B) Bateman & Scott (1990, fig. 9a); (C) Scott *et al*. (2019, fig. 1d); (D) Taylor *et al*. (2009, fig. 14.162) after Rothwell (1981, Plate III.5); (E, F) Jin *et al*. (2012, fig. 1l, m: CC BY); (G) Rothwell & Wight (1989, plate 1.2).

Although harshly criticised as ‘highly artificial’ by some observers (e.g. Taylor, Taylor & Krings, 2009, p. 519), the division of Palaeozoic ovules into three groups that was advocated by Seward (1917) – formalised as Lagenostomales, Trigonocarpales and Cardiocarpales – remains attractive as a pragmatic starting point for their taxonomic classification (Hilton *et al*., 2003). Examples of each of these three groups are compared in Figs 6 and 7. Our analysis shows that no single morphological character is guaranteed to allow attribution of a particular ovule to any of the three groups, but in each case, three or four characters can usefully be combined to yield a reliable taxonomic assignment.

Lagenostomalean seeds (a group here unconventionally shortened to ‘lagenocarps’ for convenience) have an integument that is partially or wholly adnate to the nucellus, whereas in trigonocarpalean and cardiocarpalean seeds the integument is attached to the nucellus only in a narrow basal region. Moreover, the nucellar apices of lagenocarps are comparatively complex and tripartite, a domed pollen chamber extending distally into a much narrower cylindrical salpinx (*sensu* Gordon, 1941; Long, 1961b; Hilton & Bateman, 2006), located above a parenchymatous central column of broadly similar size and shape that projects perpendicularly upward from the membranous pollen chamber floor (Figs 1, 2, 6A and 7A–C). The integumentary lobes form at best only a rudimentary micropyle, functional micropyles being absent from most – arguably all – lagenocarps. In comparison, trigonocarps and cardiocarps have a smaller, simpler pollen chamber with a thinner membranous floor, but they consistently possess a narrow functional micropyle circumscribed by an integument that otherwise is fully fused and wholly encloses the nucellar tissue (Figs 6B–F and 7D). When viewed in transverse section, the majority of lagenocarps and all trigonocarps show radial symmetry, whereas all cardiocarps show either bilateral or 180° rotational symmetry [*sensu* Rothwell (1986); Seward's (1917) original concept of cardiocarps encompassed only those ovule‐species showing 180° rotational symmetry]. Also, the majority of cardiocarps are characterised by possessing integumentary bundles that pass through the sclerotesta and by exhibiting a surrounding sarcotesta that is multi‐layered. With regard to overall size, few lagenocarps reach the 4 mm maximum girth that is exceeded by all trigonocarps and most cardiocarps.

Our strict consensus seed‐tree (Fig. 3) supports earlier, less well‐sampled Palaeozoic seed‐trees (Hilton *et al*., 2003; Seyfullah *et al*., 2010) in showing these three traditional groups to be cohesive (i.e. non‐polyphyletic). A paraphyletic ‘Lagenostomales’ gives rise to a paraphyletic ‘Trigonocarpales’ which in turn gives rise to the ‘Cardiocarpales’ – a taxonomic order that can be regarded as monophyletic provided that, as here, it is circumscribed sufficiently broadly (Hilton *et al*., 2003).

#### (b) Seed‐species potentially intermediate to the three groups: ‘missing links’?

Nonetheless, these three historical taxa remain equivocal as precise groupings. Firstly, we note that the Trigonocarpales were resolved as monophyletic sister to the Cardiocarpales in the seed‐tree of Seyfullah *et al*. (2010), whereas here no unique synapomorphy was found. Consequently, trigonocarps form two monophyletic groups, epitomised by *Stephanospermum* and *Pachytesta* respectively; only in the latter do vascular bundles pass through the nucellus. Admittedly, the present topology (Fig. 3) draws particular attention to three seed‐species that occupy positions seemingly transitional between the three groups. The branch separating the Lagenostomales from the Trigonocarpales is the best‐supported among the branches that form the spine of the tree (BS 96%) but it subtends a node from which diverges just a single seed‐species, the late Mississippian *Rhynchosperma quinnii* (Dunn, Rothwell & Mapes, 2002). Although this seed possesses most of the derived characteristics required for a typical trigonocarp, it has retained the more typically lagenocarp features of limited nucellar–integumentary fusion and an unlayered sarcotesta; it has therefore adopted the phylogenetic position predicted for this seed‐species by Dunn *et al*. (2002). Immediately below *Rhynchosperma*, *Eurystoma angulare* is placed as the most derived lagenocarp, primarily because it possesses a well‐stratified sclerotesta – a condition more typical of the succeeding trigonocarps.

Even more intriguing are the stepwise departures of first the late Pennsylvanian *Albertlongia incostata* (Taylor, 1967) and then the late Permian *Muricosperma guizhouensis* (Seyfullah *et al*., 2010); both of these seed‐genera are located above the *bona fide* trigonocarps but below all incontrovertible members of the Cardiocarpales (Figs 3 and 4). However, *Albertlongia* deviates from typical trigonocarps only in possessing a layered sarcotesta, and *Muricosperma* deviates from typical cardiocarps primarily in showing partial rather than complete fusion of integument to nucellus. Moreover, the relatively primitive position of *Muricosperma* in our tree may simply reflect optimisation anomalies resulting from matrix cells that we were obliged to score as ‘unknown’ because we lacked detailed knowledge of its pollen chamber. Thus, these ostensible phylogenetic intermediates may not actually be quite as intermediate as they seem. For example, we cannot wholly reject the possibility that *Muricosperma* may represent an early‐divergent cycad, given that vegetative remains of cycads were recently identified in the same geological formation.

## IV. TESTING THE CREDIBILITY OF THE SEED PHYLOGENY

### (1) Comparison with whole‐plant phylogenies

#### (a) Basic principles

The most obvious, and arguably most effective, way to test the likely rigour of our fossil seed‐tree is to compare the relationships inferred here among our seed‐species with the relationships inferred in previous cladistic studies that were based on wholly or partially reconstructed fossil taxa, including conceptual whole‐plants known or suspected to have borne some of the seed‐species included in our seed phylogeny. The approach of treating whole‐plant trees as relative truths is an effective way of assessing the relative accuracy of phylogenetic signal from contrasting organs of the plant as they compete for the honour of providing the strongest phylogenetic signal (Bateman & Simpson, 1998).

The seed‐taxa analysed during the present study are best discussed in terms of six prior ‘whole‐plant’ groups (Fig. 3): hydrasperman pteridosperms (*s.l*.: here equated with the seed group lagenocarps), medullosan pteridosperms (here equated with the seed group trigonocarps), callistophyte pteridosperms (represented here by *Callospermarion* only), cordaites, voltzialean conifers (represented here by *Emporia* only) and glossopterids (the latter four lineages here collectively equated with the cardiocarp seed‐group, thus circumscribed in the broadest possible sense of this seed‐based taxonomic concept).

The two key topological questions consistently asked of any reconstructed phylogeny are: (*i*) in what relative temporal sequence did the taxonomic groups of interest arise and (*ii*) is each group perceived as monophyletic, paraphyletic or polyphyletic? It is worth noting at this point that, in many previous ‘whole‐plant’ phylogenies, all (e.g. Doyle & Donoghue, 1986, 1992; Nixon *et al*., 1994; Doyle, 2006) or most (Rothwell & Serbet, 1994; Hilton & Bateman, 2006; Rothwell & Stockey, 2016) of the six groups listed above were each represented only by a single place‐holding taxon, thereby precluding any test of their presumed monophyly through constructing ‘whole‐plant’ trees. Admittedly, Hilton & Bateman (2006; echoed by Friis *et al*., 2007) did include in their analysis six hydrasperman pteridosperms, which were poorly resolved as paraphyletic, and three cordaites, which were poorly resolved as monophyletic (Fig. 5A). But with these modest exceptions, cordaite monophyly has not previously been tested. More recently, Toledo, Bippus & Tomescu (2018) included eight putative hydraspermans in their analysis, but the resulting trees were poorly resolved.

In the present seed‐based topology (Fig. 3), paraphyletic hydraspermans give rise to paraphyletic medullosans which in turn give rise to paraphyletic cordaites. Embedded within the cordaites are the three remaining ‘whole‐plant’ groups, each resolved as putatively monophyletic. Listed in order of their relative dates of origin, as inferred from their first convincing appearances in the fossil record, they are the glossopterids, voltzialean conifers and callistophytes. All three of the putatively monophyletic groups are considerably less well‐sampled in our study than are the putatively paraphyletic groups that originated earlier in the history of seed‐plants.

#### (b) Summary of selected comparable whole‐plant trees

All of the comparable whole‐plant phylogenies considered here recognise lagenostomaleans (‘hydraspermans’) as the earliest group bearing true seeds, consistently originating immediately prior to the medullosans. However, the next group to diverge in the majority of studies is the callistophytes – solitary in most phylogenetic studies (Fig. 5) but placed as sister to glossopterids plus peltasperms in the tree of Rothwell & Serbet (1994, fig. 1). One topology that deviated from this rule is the earliest of the trees produced by Doyle & Donoghue (1986, fig. 4), which interpolated between the medullosans and callistophytes a clade consisting of cordaites plus conifers plus ginkgos. Cordaites are traditionally regarded as sister to (and often as the presumed ancestral plexus eventually giving rise to) conifers, although in some morphological phylogenetic studies they formed one component of a polytomy that predictably included conifers but also encompassed other groups of seed plants (Nixon *et al*., 1994, fig. 3; Rothwell & Stockey, 2016, fig. 28). One notable exception was the study by Doyle (2006, fig. 6), which placed cordaites as sister to a clade consisting of most seed‐plant groups other than the earlier‐diverging hydraspermans, medullosans and callistophytes; cordaites diverged immediately prior to the sequential divergences of first ginkgos, then conifers, and then glossopterids. This relatively late divergence of glossopterids, placed as arguably the earliest divergent of the much‐discussed ‘anthophyte/glossophyte’ clade, characterises the majority of morphological cladistic studies (Doyle & Donoghue, 1986, 1992, fig. 2; Doyle, 2006; Hilton & Bateman, 2006, fig. 10) (Fig. 5A). However, along with other Mesozoic pteridosperms, glossopterids were placed lower in the trees of Rothwell & Serbet (1994) and Rothwell & Stockey (2016) (Fig. 5B), and they formed part of the disappointingly inclusive 10‐taxon polytomy that dominated the central portion of the consensus tree presented by Nixon *et al*. (1994).

#### (c) Comparison of present seed‐tree with previous whole‐plant trees

In summary, the paraphyly of the hydraspermans and of the medullosans that is evident in our present tree (Figs 3 and 4) accords acceptably well with topologies resulting from previous whole‐plant‐based analyses (Fig. 5). Routine representation of cordaites as a single entity in previous whole‐plant studies means that, unfortunately, those earlier topologies are unable to challenge the cordaite paraphyly that is implied by our tree. Moreover, the role suggested here for cordaites as ancestral to conifers *via* voltzialean intermediates supports a hypothesised genealogical relationship long cherished by many palaeobotanists (e.g. Florin, 1950, 1951, 1954; Rothwell, 1982b, 1988; Mapes & Rothwell, 1991; Spencer *et al*., 2015). The position of glossopterids as either sister to, or shallowly embedded within, cordaites (depending on which taxonomic assignments are imposed on the seed‐genera *Plectilospermum* and *Sergeia*) is novel but credible following recent reassessment of glossopterid homologies (McLoughlin & Prevec, 2019, 2021). Moreover, it also remains consistent with the widely recognised role of glossopterids as the earliest‐diverging lineage of the much discussed ‘anthophyte’ clade – the group of higher taxa that was hypothesised in some studies to have reached what is widely (if subjectively) regarded as a phylogenetic acme by generating the angiosperms. The only aspect of our seed‐based phylogeny that appears unequivocally discordant with earlier studies is the placement of the origin of the callistophytes midway through the diversification of the cordaites, rather than prior to their origin. However, in the absence of previous phylogenetic studies that included multiple cordaite taxa (an essential prerequisite for testing cordaite monophyly), even this unexpected inference merits serious consideration in future phylogenetic studies.

#### (d) Equivocal taxonomic assignment of some fossil seed‐species limits comparison

We should note at this point that several of the seed‐species included in our matrix have not yet been assigned with confidence to higher taxa. These include multiple seed‐species that occupy key locations along the spine of our tree, thus constituting potential ‘missing links’ between previously circumscribed higher taxa. Particular interest is inevitably evoked by taxa which bracket the ‘spine’ branches that separate the three informal groups to which seed‐species have traditionally been assigned (i.e. ‘Lagenostomales’, ‘Trigonocarpales’ and ‘Cardiocarpales’; Fig. 3). The unusually long branch separating Lagenostomales from Trigonocarpales is here bridged by *Eurystoma angulare* from the early Mississippian of northeast England (Long, 1960a, 1965) and by *Rhychosperma quinnii* from the late Mississippian of Arkansas (Dunn *et al*., 2002). The potentially long branch separating Cardiocarpales from Trigonocarpales is bridged by two seed‐genera of especially ambiguous affinities: first *Albertlongia incostata* from the mid‐Pennsylvanian of Illinois (Taylor, 1967), then *Muricosperma guizhouensis* from the latest Permian of southern China (Seyfullah *et al*., 2010). These seed‐species inevitably attract particular interest, although we should note that none of these taxa diverged alone from the spine of the comparable seed tree produced by Seyfullah *et al*. (2010).

Uncertain assignments to higher taxa in the present seed tree are most frequent in the most derived clade within cardiocarps *s.l*., which features as its earliest‐diverging member *Nucellangium glabrum* (Andrews, 1949). The 13 seed‐species comprising this clade, dominantly of late Pennsylvanian age, represent eight poorly differentiated seed‐genera that are clearly in need of taxonomic revision to achieve a better fit with the principle of monophyly. This clade is of particular interest as it may include early‐diverging cycads, whose seeds have long been sought in rocks of Pennsylvanian–Permian age. Both morphological (e.g. Spencer *et al*., 2017; Rothwell *et al*., 2022) and molecular (e.g. Clarke *et al*., 2011; Stull *et al*., 2021) phylogenies suggest that cycads should have been present at that time (Coiro *et al*., 2023), and potential candidates exist among the more ambiguous two‐dimensional compression floras (e.g. Wachtler, 2013).

### (2) Comparison with first appearances in the fossil record

Figure 4 recasts our seed tree against stratigraphic time through the 107–114 Myr period that, on present evidence, separated the earliest seeds of the latest Devonian from the much‐debated end‐Capitanian extinctions (e.g. Bond *et al*., 2010; Stevens *et al*., 2010) and the universally recognised, profound Permian–Triassic mass extinction (e.g. Dal Corso *et al*., 2022). This exercise provides a further valuable test of the credibility of our topology, and also usefully highlights six time intervals that have not yet yielded anatomically preserved seeds of acceptable quality and so constitute potentially problematic gaps in our data. Fortunately, four of these gaps are comparatively short (*c*. 3 Myr; all Mississippian) and do not appear to interrupt the phylogenetic signal significantly. Less easily dismissed are the 7 Myr gap in seed data for the mid‐Visean, which may have witnessed the origin of the trigonocarps, and especially the later 34 Myr gap that constitutes 72% of the Permian period. Among the seed‐species analysed here, this gap is spanned by only two putative members of late Pennsylvanian–early Permian seed‐genera, both from southern China: the trigonocarp *Stephanospermum truncatum* (Spencer *et al*., 2013b) and the cardiocarp *Cardiocarpus huopuensis* (Wang *et al*., 2006). It is also possible that lagenocarps extended as far as the late Permian in China, as possible *Conostoma* seed‐species have been found but require stronger characterisation (Hilton *et al*., 2001). This substantial lacuna in knowledge should in future be preferentially targeted when prospecting for anatomically preserved fossil seeds.

With regard to assessing further the credibility of our topology, we are encouraged by the fact that relationships among seed‐species implied by our tree are broadly consistent with the first appearances of taxa in the fossil record (Fig. 4). The most obvious exceptions are the seed‐genera *Sergeia* (the sole putative representative of the vojnovskyalean conifers) and *Callospermarion* (the sole representative of the callistophyte pteridosperms). Both genera are nested – deeply in the case of *Callospermarion* – within clades otherwise represented by considerably younger seed‐species (61 Myr younger in the case of *Sergeia*). It is tempting to ask whether these two lineages actually diverged lower in the phylogeny than is suggested by our seed‐based topology, in placements more consistent with previous cladistic reconstructions based on the conceptual whole‐plant species considered to have borne these seed‐species (e.g. Doyle & Donoghue, 1986; Hilton & Bateman, 2006; Rothwell & Stockey, 2016). By contrast, as suspected by Dunn *et al*. (2002), the end‐Mississippian age of *Rhynchosperma* fits well with its inferred phylogenetic position as transitional between the Lagenostomales and Trigonocarpales (Fig. 3).

Supposedly earlier fossil occurrences having failed to survive close scrutiny, conventional wisdom (e.g. Anderson, Anderson & Cleal, 2007; Seyfullah *et al*., 2010) now states that the cordaites first appeared in the fossil record in the Bashkirian (early Pennsylvanian), relative to the conifers in the Gzhelian (latest Pennsylvanian) and the glossopterids in the Asselian (earliest Permian; Fig. 4). By contrast, our ovule tree implies that the glossopterids – or at least, close relatives of the glossopterids – originated before both the cordaites and the voltzialean conifers. Arguably the most novel inference suggested by our ovule‐based tree is that the cordaites may have played a greater role in plant macroevolution than is generally believed, giving rise not only to the conifers but also independently to the callistophytes and perhaps even to the glossopterids. This hypothesis receives tentative support from recent morphological re‐interpretations of glossopterids that reduces the supposed morphological disparity separating them from cordaites (Nishida *et al*., 2013; McLoughlin & Prevec, 2019, 2021). Also relevant is the presence towards the apex of our ‘unknown affinities cardiocarps’ clade of two species of the seed‐genus *Diplotesta* (Fig. 3) that were attributed by Galtier (2008) to the cordaite cone *Renaulticonus*.

### (3) Comparison with phenetic cohesion and disparity patterns

We sought to explore further the cohesion of the three main seed groups, and to estimate relative disparities within them, by subjecting our morphological cladistic matrix to phenetic analysis through multivariate ordination.

**Fig. 8:**
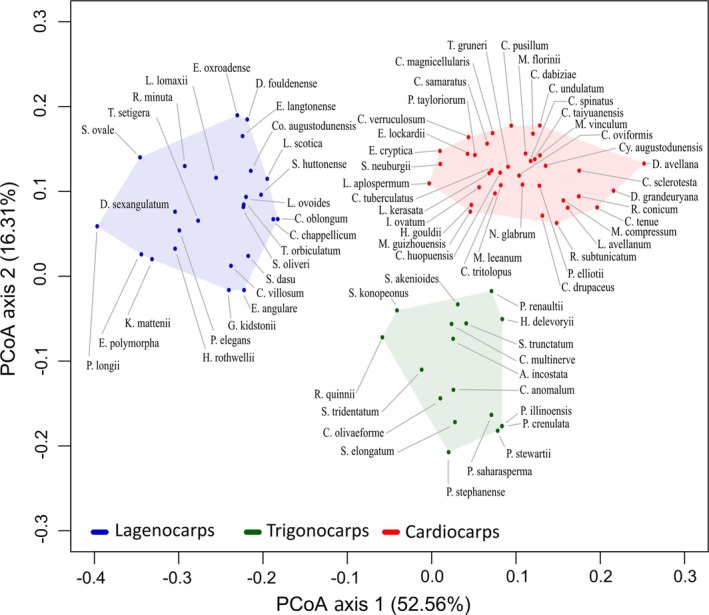
Plot of the first two principal coordinates (PCoA axes 1 and 2) based on morphological characters for 79 Palaeozoic seed‐species, showing lagenocarps, trigonocarps and cardiocarps as three cohesive groups and suggesting preferred placements for phylogenetically intermediate seed‐species such as *Rhynchosperma quinnii*, *Albertlongia incostata* and *Muricosperma guizhouensis*. Note the great disparity in strength between the first and second axes.

The plot of the first two principal coordinates (Fig. 8) encompassed an impressive 69% of the total variance. Searching within each of the three prior groups – lagenocarps, trigonocarps and cardiocarps – failed to reveal any patterns of obvious interest, but it did yield helpful insights into the relationships among the three main groups and into the morphospace that each encompasses. The first coordinate reveals a clear morphological discontinuity separating the lagenocarps from the remaining taxa, thus mirroring the longest, strongest branch on the spine of our phylogeny, located immediately below *Rhynchosperma* (Fig. 3). The second coordinate (admittedly much weaker) offers smaller, but nonetheless substantial, separation of the trigonocarps from both the cardiocarps and the lagenocarps. Thus, the cohesion of the three groups receives further support.

**Fig. 9:**
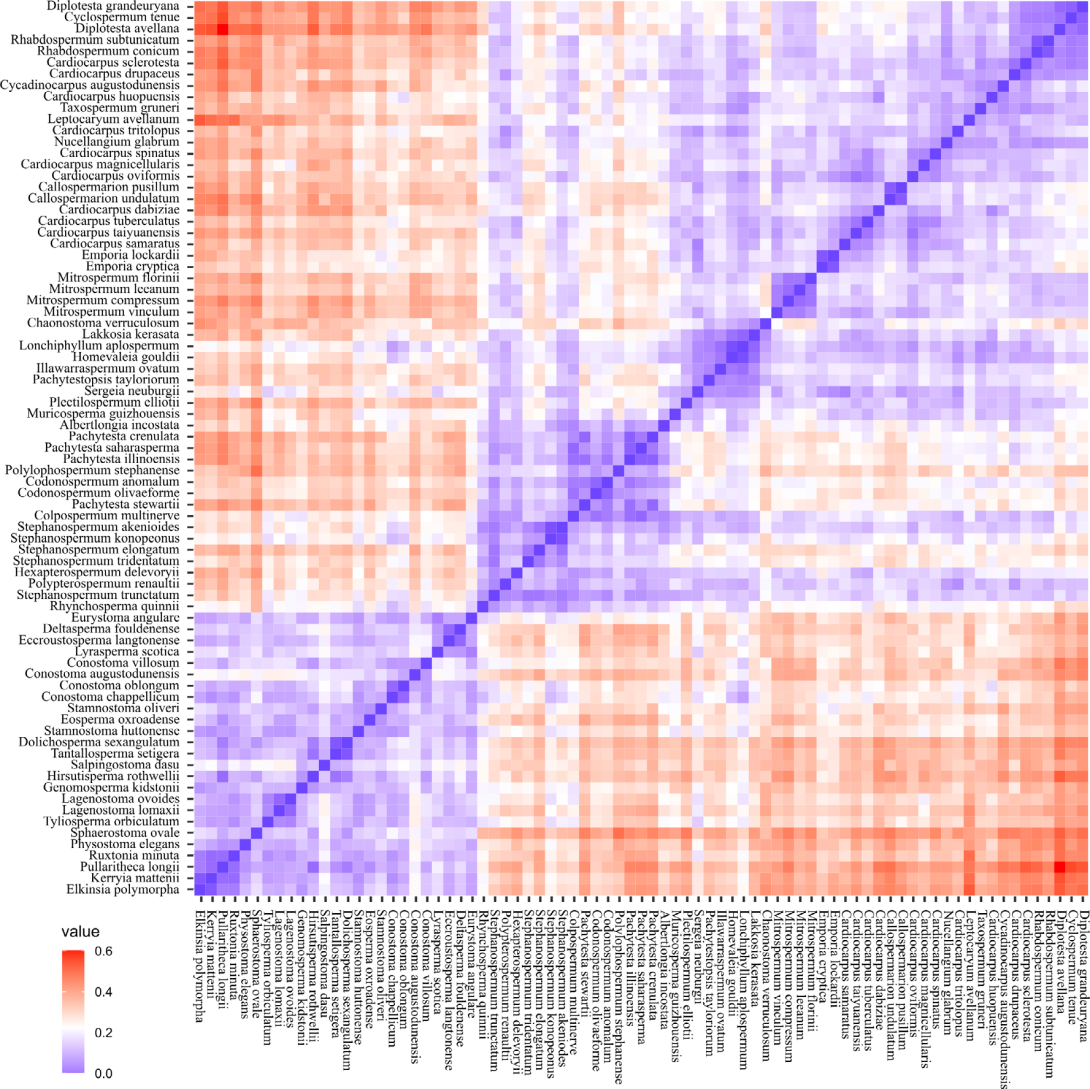
Heatmap of morphological similarities among 79 Palaeozoic seed‐species, represented as disparity estimates derived from Gower similarity values. The boundaries separating lagenocarps from trigonocarps and trigonocarps from cardiocarps are evident, as is the intermediate morphology of *Rhychosperma quinnii*.

Viewed from the perspective of disparity, the three groups occupy broadly similar volumes of morphospace (Fig. 8), though the lagenocarps are slightly more scattered, despite representing a smaller taxonomic sample than the cardiocarps. Thus, overall levels of disparity in seed morphology may have experienced a modest decrease as seed‐plants evolved through the Palaeozoic. The accompanying heatmap quantifying disparity (Fig. 9) reveals comparatively high disparity between the lagenocarps and both the trigonocarps and the cardiocarps (especially with respect to *Leptocaryum* and *Diplotesta* – taxa placed within the clade of unknown affinities which constitutes that portion of our tree immediately above *Cardiocarpus tritolopus*: Fig. 3). Also, the heatmap provides additional evidence that *Rhynchosperma* is a potential transitional taxon linking lagenocarps to trigonocarps, as it has roughly equal affinities with both groups.

### (4) Comparison with molecular phylogenies of extant gymnospermous groups

**Fig. 10:**
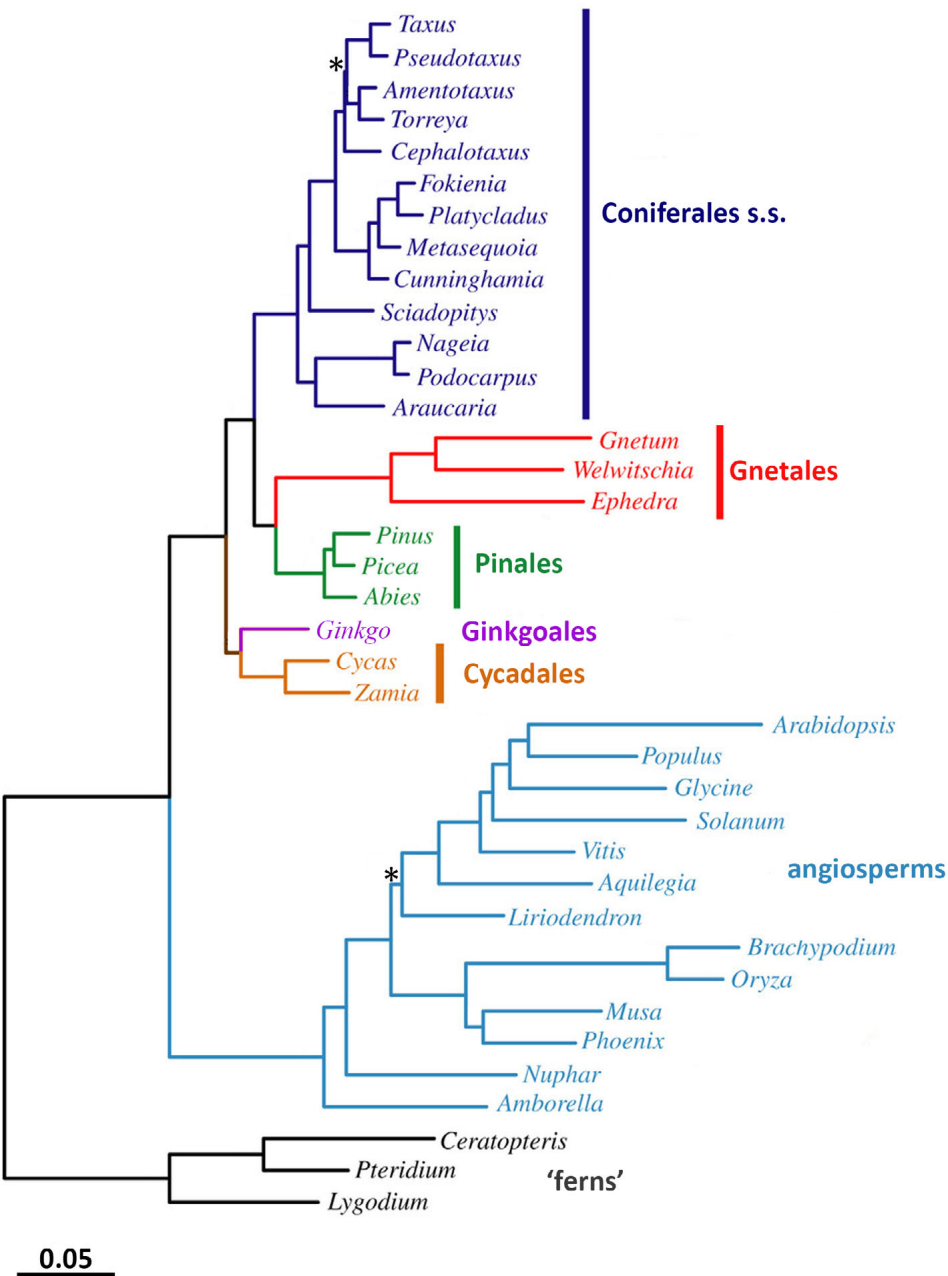
Molecular phylogeny showing relationships among genera of six major groups of extant seed‐plants. Likelihood tree constructed using RAxML (Randomized Axelerated Maximum Likelihood); all branches except those asterisked received 100% bootstrap support. Based on first plus second codon nucleotides for 1308 groups of orthologous nuclear genes. Modified from fig. 1 of Ran *et al*. (2018). Reproduced through pemission of the Licensor through PLSclear.

Phylogenies of major seed‐plant groups that include fossil taxa and are based solely on morphological characters have been notoriously incongruent with those analysing only extant taxa and relying solely on DNA‐based characters (discussed by Bateman, Hilton & Rudall, 2006; Coiro, Chomicki & Doyle, 2018; Bateman, 2020). Less than half of the major lineages of gymnosperms that ever existed persisted sufficiently long to leave at least one representative in the extant flora; the credibility of topologies based solely on extant taxa (e.g. Burleigh & Mathews, 2004; Graham & Iles, 2009; Barba‐Montoya *et al*., 2018; Ran *et al*., 2018; Yang *et al*., 2022) is challenged by these crucial absences. When considering morphological characters, some authors have chosen to impose upon them a phylogenetic structure obtained through molecular analysis, effectively prioritising character‐rich taxon‐deficient matrices over taxon‐rich character‐deficient matrices (e.g. Doyle, 2006; Shi *et al*., 2021). We find this approach philosophically questionable, believing that it is more important to seek explanations for the radically different morphological versus molecular placements of extant groups, notably the angiosperms and the Gnetales (compare Fig. 5 with Fig. 10). In recent molecular trees, Gnetales tend to place as sister to pines, thereby dividing Coniferales *s.l*. into Coniferales *s.s*. and Pinales (Fig. 10). Relationships among the three remaining extant groups of gymnosperms differ less radically between morphological and molecular studies, cycads (two extant families) and ginkgos (one extant family) typically being placed below a monophyletic group of seven extant conifer families (*cf*. Burleigh & Mathews, 2004; Graham & Iles, 2009; Ran *et al*., 2018; Yang *et al*., 2022). Most studies, both molecular and morphological, place ginkgos either above, or more often as sister to, cycads (Figs 5 and 10).

However, it is important to bear in mind that most of the genera depicted in Fig. 10 originated later than the period of time covered in the present review, and that the major groups represented in the molecular tree are happenstance survivors of past seed‐plant diversity. Although molecular trees should indeed be used as independent tests of equivalent trees constructed on morphological foundations, they cannot directly address the question of whether our fossil seed trees accurately represent our fossil whole‐plant trees; the vulnerability of DNA to decay renders this particular debate entirely morphological.

## V. RELATIVE EVOLUTIONARY LABILITY OF CONTRASTING CATEGORIES OF CHARACTER

**Fig. 11:**
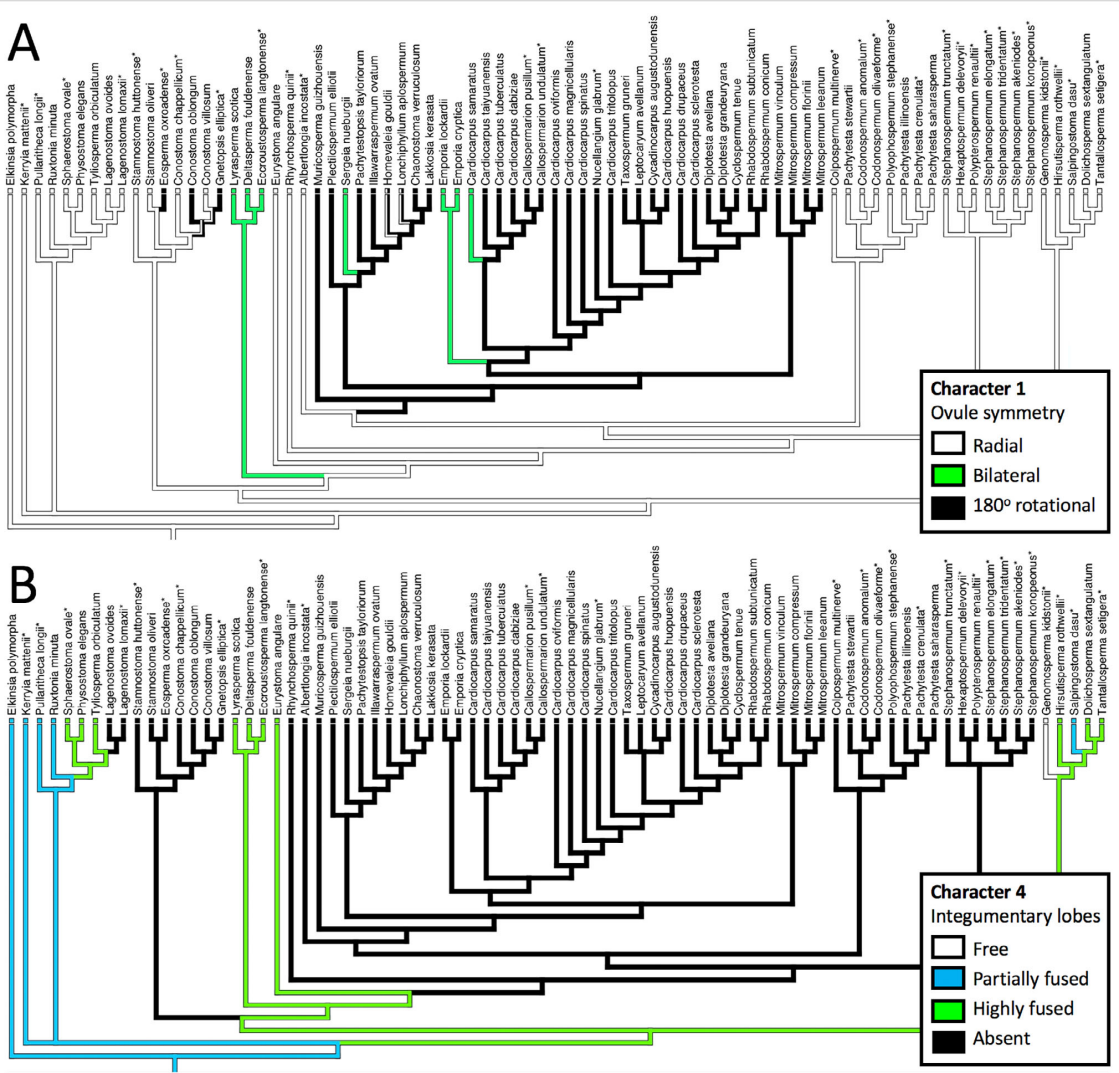
Optimisations across our strict consensus tree (Fig. 3) of characters representing ovule symmetry (A) and integumentary lobe fusion (B).

**Fig. 12:**
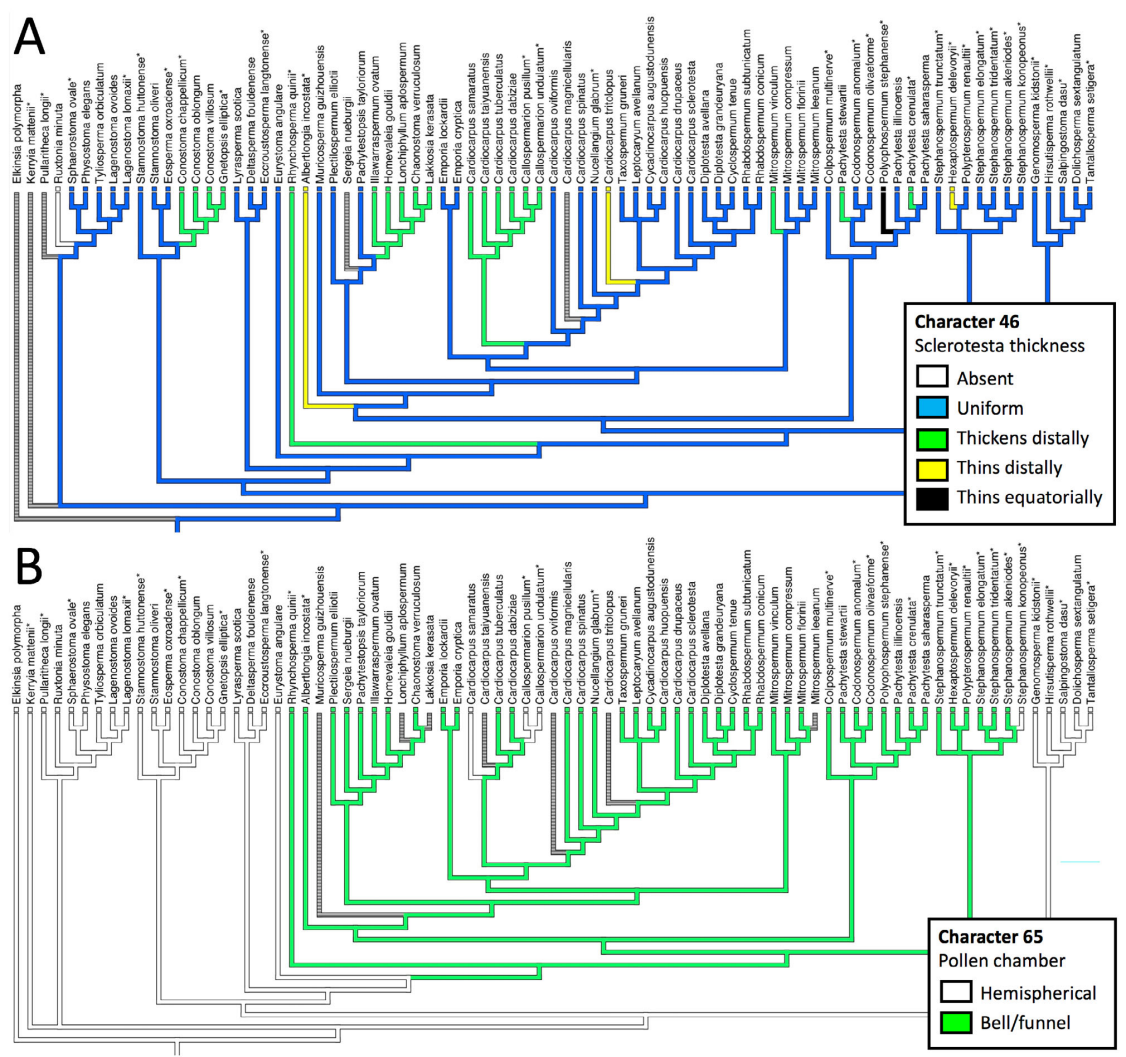
Optimisations across our strict consensus tree (Fig. 3) of characters representing variation in sclerotesta thickness (A) and pollen chamber shape (B).

All 89 characters were optimised across our tree in sequence; four examples of contrasting behaviour are shown in Figs 11 and 12. Ovule symmetry (Fig. 11A) is a classic character of greater complexity than first appears (Rothwell, 1986). Most cardiocarps show 180° rotational symmetry, whereas all trigonocarps and most lagenocarps show radial symmetry, but sprinkled among both lagenocarps and cardiocarps are seed‐species that have diverged into bilateral symmetry. Figure 11B shows contrasting degrees of fusion among integumentary lobes of lagenocarps, but the general trend of increasing levels of fusion does not resolve as the steadily progressive transition series envisaged by some observers (e.g. Andrews, 1963). Trigonocarps and cardiocarps reliably possess unlobed (i.e. fully fused) integuments. Figure 12A explores the subtleties of proximal–distal change in sclerotesta thickness, wherein various deviations from uniformity characterise only single or small monophyletic groups of seed‐species within all three major groups. Lastly, Fig. 12B readily distinguishes the domed pollen chambers of the lagenocarps from the campanulate/funnel‐shaped pollen chambers of the trigonocarps and cardiocarps, but also reveals reversals to domes in a few phylogenetically isolated cardiocarps.

Overall, our character optimisations left the impression that different categories of character were more prone to evolutionary change in contrasting groups of early seeds. We attempted to investigate this possibility by assigning each of our 89 utilised characters to one of five categories according to whether they represent the architectural framework of the seed, its wall structure, its external topography, smaller‐scale cellular features, or were able to influence directly the pollination process. We then counted the number of state‐transitions experienced by each character in each of the three main groups of seed‐species, adjusted for the number of seed‐species in each group that could be scored for that character (i.e. making allowances for the reduced opportunity for change caused by missing values). This approach allowed us to calculate values for mean and standard deviation for each category of character in each of the three taxonomic groups of seeds. Homoplasy in cladograms is traditionally assessed by calculating a consistency index and retention index for each character across the entire tree, whereas here the task facing us was more complex; we sought values for each of three segments of our tree, two of which were resolved as paraphyletic.

The results of this exercise (summarised in Table 1) are undeniably crude, as demonstrated by standard deviation values that approximate values of the associated means. When considering these figures, it is important to bear in mind that the cardiocarps represent greater diversity of formalised higher taxa than do the lagenocarps or the trigonocarps. Despite this fact, the three groups showed remarkably similar average overall frequencies of change per character per seed‐species: 0.082 in lagenocarps, 0.086 in trigonocarps and 0.088 in cardiocarps. However, when comparison was restricted to particular categories of character, significant differences among taxa became apparent.

**Table 1:** Comparison of the frequency of character‐state changes in three portions of the consensus tree presented in Fig. 3, calculated as the number of state transitions per character per scorable seed‐species. Figures given are mean (standard deviation) values calculated for each character assigned to that category, as limited to the specified portion of the tree.

Category of character	Number of characters	Lagenocarps	Trigonocarps	Cardiocarps
Architectural	25	0.105 (0.108)	0.059 (0.084)	0.098 (0.078)
Wall structure	8	0.075 (0.065)	0.156 (0.135)	0.124 (0.118)
External topography	18	0.043 (0.074)	0.111 (0.093)	0.075 (0.098)
Cellular scale	20	0.129 (0.071)	0.051 (0.132)	0.106 (0.091)
Pollination‐related	18	0.059 (0.092)	0.052 (0.050)	0.035 (0.053)

Specifically, greater architectural experimentation is evident among the lagenocarps and cardiocarps than among the trigonocarps, which collectively share a relatively conservative seed bauplan. The same taxonomic distinction is evident among cellular‐scale characters; again, the trigonocarps proved most conservative. Wall structure and external topography contribute considerably less to lagenocarps, primarily reflecting the fact that they lack the potential for specialisation among layers within the multi‐layered integuments that characterise the trigonocarps and cardiocarps (*cf*. Fig. 6A–D). Characters relating to pollination are least prone to change in general, but particularly so among cardiocarps, presumably reflecting powerful evolutionary constraints operating on a pollination syndrome that – as discussed at greater length in Sections VI and VII – had become so strongly dependent on the capture of pollen (as opposed to pre‐pollen) *via* a pollen drop extruded through a micropyle (Fig. 7).

Overall, there is insufficient evidence, either in the character change rates (Table 1) or in the disparity assessments of Section IV.3, to claim an early explosive radiation of form following the origin of gymnospermy – one that subsequently became subject to increasingly more effective evolutionary constraints (*contra* Gould, 1989). However, anyone still seeking evidence of an early phase of morphological experimentation could justifiably point to the comparatively wide range of architectures (and indeed of seed sizes) evident among Mississippian lagenocarps. In addition, it is important to consider the possibility that seeds are relatively conservative organs, and that the remaining 10 or so organs that constituted these gymnospermous plants could have been indulging in mosaic evolution *sensu* Stebbins (1984), diversifying radically while maintaining a seed morphology that differed only subtly among species.

## VI. INFERRED BIOLOGICAL PROPERTIES

### (1) Pollination and fertilisation

#### (a) Haustorial zooidogamy

Transitions in the structures and mechanisms underpinning pollination and fertilisation in early gymnosperms surely represent critical evolutionary steps, but they can only realistically be inferred by integrating the few fragmentary pieces of circumstantial evidence from the Palaeozoic fossil record into a framework of direct observations made on extant gymnosperms, particularly the earliest‐divergent extant groups: cycads and *Ginkgo*. Both lineages date back to the Palaeozoic and so are directly relevant to the present study. Possible cycads of the late Pennsylvanian are succeeded in the early Permian by fossils that broadly resemble the extant family Cycadaceae (Kungurian age *sensu* Fig. 4: e.g. Gao & Thomas, 1989). In the case of Ginkgoales, equivocal evidence of *Ginkgo*‐like plants in the lower Permian becomes more robust by the upper Permian (Lopingian age; e.g. Fischer *et al*., 2010).

Both extant cycads and extant ginkgos produce large, fleshy seeds broadly reminiscent of those borne by Palaeozoic trigonocarps and cardiocarps. Most extant cycad seeds appear radially symmetrical in external view, whereas *Cycas* and *Ginkgo* show modest deviations from equidimensional equators that result in relatively subdued 180° rotational symmetry (e.g. Spencer *et al*., 2017; Zumajo‐Cardona, Frangos & Stevenson, 2021; Rothwell *et al*., 2022; Muto *et al*., 2024). Cycads and ginkgos lack the siphonogamy that characterises extant conifers, gnetaleans and angiosperms, wherein multiple non‐motile nuclei, including the ‘male’ nuclei, are delivered directly to the archegonium *via* cytoplasmic streaming through an elongate pollen‐tube (e.g. Rothwell, 1980; Labandeira *et al*., 2007; Rudall & Bateman, 2007). Rather, it is likely that all of the Palaeozoic taxa included in the present study possessed motile sperm. In the case of ginkgos, wind‐borne pollen grains land on the meniscus of a classic gymnospermous pollination drop (Fig. 7E), which is then withdrawn by the ovule (Fig. 7F) *via* a relatively long, narrow channel through the integument that is termed the micropyle, drawing the pollen grains toward the distal surface of the nucellus where a few (typically three) archegonia have recently developed. Meanwhile, the homologue of the pollen tube instead fulfils a plesiomorphic, and radically different, role as a branched haustorium supplying nutrients and water derived from the nucellus (e.g. Johri, 1992; Poort, Visscher & Dilcher, 1996; Rudall, 2021; Muto *et al*., 2024). Although most extant cycads are pollinated biotically, by beetles and weevils, they too rely on pollination drops to transfer the pollen within the integument.

Remarkably, the occurrence of this relatively derived mode of zooidogamy has been demonstrated convincingly in at least two of the fossil seed‐genera included in our study: the late Pennsylvanian *Callospermarion* [seed of the derived pteridosperm *Callistophyton* (Rothwell, 1970, 1972, 1977, 1980)] and the late Permian glossopterid *Homevaleia gouldii* (Nishida, Pigg & Rigby, 2003; Nishida *et al*., 2007; Pigg & Nishida, 2006). Zooidogamy is also suspected to have featured in the Palaeozoic cordaites and Emporiaceae (Mapes & Rothwell, 1991), thus characterising at least the basal two thirds of our cardiocarp *s.l*. clade (Fig. 3). We are inclined to view this condition as the null hypothesis for most Palaeozoic gymnosperms.

#### (b) Non‐haustorial zooidogamy

By contrast, both the trigonocarps and evolutionarily preceding lagenocarps are considered to have maintained a more primitive mode of zooidogamy, producing not pollen *sensu stricto* (i.e. capable of generating a pollen tube through a distal aperture, the leptoma) but rather prepollen that simply released motile antherozooids within the distal region of the ovule from a proximal triradiate aperture, thereby requiring the presence of at least a film of free water for the antherozooids to reach and fertilise the archegonia (Chaloner, 1970). In the case of the trigonocarps, the pollination drop enclosing the prepollen was withdrawn through a micropyle penetrating the integument, as in the cardiocarps. This role had been acquired by trigonocarps through transference of function from the lagenocarp condition, wherein the nucellus rather than the integument was responsible for channelling the emission and withdrawal of the pollination drop.

In a remarkably sophisticated integration of functional morphology and ontogeny, selective apoptosis hollowed out the apical portion of the nucellus of lagenocarps into a domed pollen chamber that became apically elongated to form a cylindrical pollen‐receiving apparatus termed the salpinx (Rothwell, 1986; Hilton & Bateman, 2006) (Fig. 1). We presume that it was from this apparatus that a pollination drop was first exuded and later withdrawn. Once the antherozooids were in contact with the floor of the pollen chamber, chemical signals encouraged expansion of the megasporangial tissue, rupturing the floor of the pollen chamber and thus permitting the antherozooids to access, and fertilise, one of several archegonia present.

#### (c) Sealing the pollen chamber

In lagenocarps, expansion of the megagametophyte forced upwards a column of nucellar tissue central to the pollen chamber floor, thereby sealing the base of the salpinx (Fig. 7A, B); this process prohibited entry of further prepollen (Rothwell, 1986; Rothwell & Scheckler, 1988; Rothwell & Serbet, 1992) or pathogens, and presumably also reduced water loss. Thus were the later stages of pollination and the fertilisation process protected from the external environment.

Trigonocarps (and also the earliest voltzialean conifers such as *Emporia*) had a pollen chamber that was less complex in shape and lacked a central column, but featured a thicker salpinx wall (Fig. 6E). Rather than employing a central plug, the entrance of the salpinx narrowed to form a nucellar beak before finally being sealed through secretion of mucilage by the inner walls (Serbet & Rothwell, 1995). Although the micropyle was not fully sealed, it is likely that this narrow orifice also limited threats posed by the outside world.

As summarised by Serbet & Rothwell (1995), the callistophytalean sealing mechanism also encompassed not only the extinct callistophytes, cordaites and glossopterids but also the extant cycads and ginkgos. These taxa retained the mucilaginously sealed nucellar beak of the trigonocarps but also introduced narrowing and sealing of the micropyle, achieved *via* cell expansion and/or cellular degradation to yield a mucilaginous glue. In more evolutionarily derived fossil groups such as bennettites and pentoxylaleans, as well as extant gnetaleans and conifers, micropylar sealing was sufficiently effective to allow abandonment of pollen chamber sealing, the pollen chamber becoming vestigial or, in a few gymnosperm lineages, lost entirely.

#### (d) Pollination drops and ovule orientation

Among extant conifers that have been shown to produce pollination drops (the great majority), two contrasting modes of bringing the pollen into close proximity with the archegonia are evident (Tomlinson, 1994). In taxa such as most Pinaceae (excluding *Tsuga* and *Larix*) and Podocarpaceae, the pollen grains are saccate and unwettable, adhering to the surface of the drop, which is invariably inverted rather than held upright. Thus, gravity alone cannot effect fertilisation; the pollination drop must therefore be actively withdrawn into the ovule. By contrast, taxa such as Cupressaceae and Taxaceae have non‐saccate, wettable pollen, the pollen being capable of sinking through the drop under gravity if, as in some cases, the ovule – and thus the pollination drop – are held upright, making pollen‐drop withdrawal possible but no longer essential. In this context, it is of considerable interest that there is a notable evolutionary transition potentially located at the evolutionary origin of the cardiocarps. Pre‐pollen associated with lagenocarp ovules is consistently non‐saccate, as is the majority of pre‐pollen associated with the trigonocarps. In cases where small sacci are present they appear non‐functional and unlikely to resist the pull exerted by gravity on these exceptionally large monolete pre‐pollen grains. By contrast, it is our impression that *bona fide*, distally germinating pollen associated with cardiocarps are reliably saccate.

If the pattern observed among extant gymnosperms is superimposed onto our seed‐species tree, the implication is that all cardiocarp seeds are likely to have been inverted at the time of pollination, whereas there is at least a possibility that some lagenocarps and/or trigonocarps were oriented upright at the time of pollination. Nonetheless, given the slender nature of the axes subtending most Palaeozoic cupules, it seems probable to us that the majority of Palaeozoic seed‐plants bore ovules (and, when present, cupules) that were inverted. If true, this would have unfortunate implications for studies that have assumed a central role for gravity when modelling wind‐mediated pollination of upright cupules only, either experimentally in wind tunnels (e.g. Niklas, 1981) or entirely *in silico* (e.g. Li *et al*., 2025). Much depends on the timing of pollination within the developmental trajectory of the ovule or ovule‐truss, which might transition from upright to pendent during ontogeny.

Our matrix includes a few characters that require correlation of seed‐species with particular cupule‐species and/or particular (pre)pollen species. Ideally, the (pre)pollen‐species thought to correspond with a particular seed‐species is demonstrated through correlating the cupule bearing the seeds with the pollen‐organ producing the (pre)pollen (Bateman & Hilton, 2009). Further evidence, albeit far less secure, comes in the form of allometry; the diameter of (pre)pollen often approximates 20% of the diameter of the corresponding salpinx when measured at its narrowest point. In most cases, only one (pre)pollen‐species is found within the pollen chamber of a seed‐species (e.g. Bateman & Rothwell, 1990), implying – but by no means demonstrating – that it represents the same whole‐plant species. In the case of one seed‐species scored for our matrix, the trigonocarp *Polypterospermum renaultii*, two prepollen species were found within the pollen chamber, but the more frequent of the two was rejected as conspecific, being saccate airborne prepollen produced in abundance by adjacent cordaite plants that would have instead borne cardiocarp seeds (Combourieu & Galtier, 1985).

#### (e) Ontogeny

The fact that almost all Palaeozoic seeds are found separated from their parent plant, and that most cupulate ovules are found separated from their cupules, means that it is extremely difficult to infer their pattern of development, which in turn distances us from our ability to interpret their evolution on the basis of those important processes collectively termed heterochrony (e.g. Gould, 1977; Rothwell, 1987; Bateman, 1994).

One notable exception to this rule is provided by the cupule *Pullaritheca* from the Tournaisian of Oxroad Bay in Scotland – one of three broadly similar but nonetheless distinguishable cupules demonstrated to host the classic hydrasperman lagenocarp seed‐genus *Hydrasperma* (Long, 1977b; Rothwell & Wight, 1989; Bateman & Rothwell, 1990). Numerous specimens obtained from two contrasting beds at the site provide examples of this ovule in three contrasting contexts: isolated mature ovules (Long, 1961b, 1977b; Bateman & Rothwell, 1990); several mature ovules packed within a compact cupule (Rothwell & Wight, 1989) (Fig. 7G); and several immature, sessile ovules distributed sporadically across the ‘placentae’ of cupules otherwise similar to that enclosing the mature ovules (Long, 1977b; Bateman & Rothwell, 1990). Isolated immature ovules were not found. Ovule size is strikingly bimodal; the dispersed and mature *in situ* ovules reach *c*. 3.5 mm long but the immature *in situ* ovules are only *c*. 1 mm long.

Although juvenile, the smaller ovules nonetheless possess well‐developed distal regions, in which the integumentary lobes are readily distinguished and the nucellus has already formed the external topography of the pollen chamber, although this region of the nucellus has not yet been hollowed out through apoptosis. A short salpinx can also be discerned. By contrast, the proximal portion of the ovules is greatly under‐developed compared with their mature manifestation, suggesting that substantial expansion of the megagametophyte occurs relatively late in ontogeny. The immature ovules give the overall impression of a balloon that has not yet been inflated, suggesting that cell proliferation (presumably dictated by the nucellus) largely precedes cell expansion. The absence of *Hydrasperma* ovules of an intermediate size, despite the large number of ovules found, suggests that development was rapid following pollination, and that only mature ovules were dispersed.

Briefly considering modern analogues, the seeds of both *Ginkgo* and cycads abort if not pollinated (Zumajo‐Cardona *et al*., 2021; Muto *et al*., 2024). Pollen capture triggers ovule enlargement, stimulating hormone signalling while simultaneously inhibiting senescence and apoptosis (and, after a one‐week delay, ceasing maintenance of any ‘unused’ pollination drops). The archesporial cell is located in the subepidermal layer of the nucellar apex, but often shifts proximally during ontogeny (Rudall, 2021). The three distal meiotic products degenerate as the chalazal element forms the single functional megaspore. This in turn forms a haploid, highly multicellular megagametophyte (embryo sac) and archegonia. Epithelial layer cells of the nucellus are both endopolyploid and secretory. However, given that a protracted delay can separate pollination from fertilisation, arguing that only fertilised ovules continue to develop to maturity can represent an assumption rather than a demonstrated fact.

### (2) Dormancy and dispersal

#### (a) Dormancy and germination

Seeds of extant cycads and *Ginkgo* are classed as recalcitrant, lacking the ability of orthodox seeds to survive longer term when subjected to drying or freezing. They have been said to show a primitive form of dormancy that is often termed morphological class (Forbis, Floyd & Queiroz, 2002; Baskin & Baskin, 2004). However, they are perhaps better viewed as lacking true dormancy. Rather, they initiate germination quickly but thereafter growth progresses unusually slowly; for example, several months elapse between pollination and fertilisation in *Ginkgo*. Such development is typical of seeds that possess low embryo: seed ratios, wherein most of the seed volume is occupied by a large nutritive megagametophyte that provides prolonged resourcing for the young plant (Linkies *et al*., 2010).

However, unambiguous embryos are absent from lagenocarps and trigonocarps; the earliest clearly demonstrated haustorial cotyledons were demonstrated in the cardiocarp seeds of the early Permian voltzialean conifer *Emporia* (Mapes, Rothwell & Haworth, 1989). They show endoscopic embryo orientation, the suspensor being located at the micropylar pole. Among extant plants, a crucial haustorial role, transferring nutrients from the female gametophyte to the sporophyte upon germination, has been demonstrated for cotyledon tips in both *Ginkgo* and *Cycas*. After critically evaluating four contrasting scenarios, Sokoloff *et al*. (2015) concluded that the presumed evolutionary derivation of cotyledons from megaphyllous leaves, combined with the failure to find embryos in dispersed lagenocarp and trigonocarp seeds, suggest that the distal part of the female gametophyte was probably released from the testa, most likely *via* the micropyle and with the embryo potentially still zygotic and fed solely by the suspensor. An origin of multiple cotyledons early in the evolutionary history of cardiocarps appears most likely. The oldest seed known to possess an embryo retained within the testa is the Middle Pennsylvanian putative cordaite *Nucellangium glabrum* (Stidd & Cosentino, 1976; Taylor *et al*., 2009) – the basalmost member of the ‘Unknown affinities’ clade that caps our seed‐species tree (Fig. 3). Later diversification into a range of more sophisticated, physiologically dictated dormancy mechanisms appears to involve considerable homoplasy (Linkies *et al*., 2010).

Examination of a wide range of Palaeozoic seeds reveals cracks that highlight the role of commissures as planes of structural weakness, apparently splitting the integument into germination valves as a means of reducing the physical constraint of the tough sclerotesta. The commissures follow the major planes of symmetry; thus, bilaterally symmetrical cardiocarps have one commissure in the major plane of symmetry whereas trigonocarps typically show three slits separated by 120° in transverse section. Similar valves characterise extant *Cycas* (Arnold, 1948; Spencer *et al*., 2017).

#### (b) Ovule and/or cupule dehiscence

Surprisingly little is known regarding seed dehiscence in these early gymnosperms. Isolated seeds and cupules of most taxa are very rarely found in organic attachment with the axes that bore them, confounding any search for possible abscission zones. The main exception is cupules bearing multiple ovules, where observers might aspire to find evidence of a detachment mechanism that would ultimately separate pedicels from the placenta of the cupule (e.g. Walton, 1949; Long, 1977b). To the best of our knowledge, such abscission zones have not been reported; an unsurprising observation in view of the negligible evidence that the plants that bore them could abscise their megaphyllous leaves (Thomas & Cleal, 1999); presentation of senescent leaves as a pendulous skirt, analogous to that of modern palm trees and tree‐ferns, appears more likely. There is circumstantial evidence that the lagenocarp *Deltasperma* lacked seed abscission; the seeds had a peduncle oriented approximately perpendicularly to their long axis, and both permineralised and charcoalified (Fig. 2G) specimens suggest that the seeds were torn away from the parent plant about 0.5 mm along the peduncle (Bateman & Rothwell, 1990). The few cases where trigonocarp seeds have been found in apparent organic attachment to megaphyllous leaves also show no evidence of abscission mechanisms and may simply have parted company from the leaf as it decayed.

The earliest‐emerging seed‐plant lineage to show unequivocally a leaf abscission zone appears to be the ginkgos (Lin *et al*., 2022). Indeed, the dense packing of numerous apparently mature *Hydrasperma tenuis* ovules within the goblet‐shaped digitate cupules of *Pullaritheca longii* (Rothwell & Wight, 1989) (Fig. 7G) encourages speculation that such cupules might in some cases have acted as the primary dispersal unit – a *de facto* ‘fruit’. A contrasting hypothesis would allow the inrolled lobes of the *Pullaritheca* cupule to eventually recurve outwards, thus freeing the by‐then mature seeds for individual dispersal, in a process parallel to the late‐stage outward curvature of the integumentary lobes demonstrated in coeval *Genomosperma* ovules by Meade *et al*. (2021) (Fig. 2A).

## VII. INFERRED EVOLUTIONARY TRENDS

Revisiting the evolutionary origin of the seed, several crucial challenges arise once the products of meiosis have been reduced to a single functional megaspore that is retained within the megasporangium throughout the cycle of pollination, fertilisation, dispersal and germination. Pollen must be captured, the male gametes conveyed to the female gametes, and the resulting embryo resourced during its early growth. Throughout this essential stage in the life history of the lineage, the megasporangium and its contents must be protected rigorously from desiccation, pathogens and herbivory (specifically, granivory). Given such a wide range of functions, it is unreasonable to expect the seed to be optimised for any particular function; seed evolution must surely involve numerous trade‐offs. These in turn necessitate a toolkit of several underlying evolutionary processes.

### (1) Localised apoptosis

Apoptosis is a category of programmed cell death in which irreversible biochemical changes ultimately eliminate ‘unwanted’ cells, either intrinsically (essentially, cell suicide) or extrinsically as a result of signals received from adjacent cells. Highly conserved specialist proteases termed caspases first initiate and then execute protein degradation, through processes that at present are far better understood in higher animals than in higher plants (e.g. Dickman *et al*., 2017). Of particular interest is vacuolar cell death, which not only consumes the protoplast but also degrades the cellulose wall of each affected cell (Wertman *et al*., 2012).

The most obvious relevance of apoptosis *sensu lato* to seed evolution is that it dictates a necessary step in the origin of the seed, specifically monomegaspory. Initially, the number of megasporocytes in the megasporagium must by definition be reduced to just one. Then, given that meiosis requires a megaspore mother cell to yield four spores, three of those spores must be aborted if only a single functional megaspore is to remain within the megasporangium (Pettitt, 1970; Bell, 1979; Bateman & DiMichele, 1994). This process is readily observed in extant heterosporous taxa, and Pettitt & Beck (1968) famously demonstrated the retention of the three aborted apicalmost products of meiosis at the distal pole of the nucellus of the Upper Devonian lagenocarp *Archaeosperma* – fossils that sadly otherwise lack anatomical preservation. Abortion of three of the four meiotic products is a phenomenon that is undoubtedly the consequence of routine apoptosis and presumably is ubiquitous among gymnosperms.

A more complex, but equally important, case of localised apoptosis is the formation of the pollen chamber, which arose in the lagenocarps but was retained by the succeeding trigonocarps and cardiocarps. The latter are more closely comparable with the seeds of extant *Ginkgo* and cycads, which have provided most of our still limited knowledge of how programmed cell death determines the functional morphology of the distal pole of the ovule (e.g. Douglas, Stevenson & Little, 2007). In the case of *Ginkgo*, local auxin concentrations dictate both female gametophyte development and nucellus degeneration (Muto *et al*., 2024). A pathway of programmed cell death in precisely pre‐defined apical regions of the nucellus is essential to create the pollen chamber (Li *et al*., 2019a; Rudall, 2021) but also appears to be necessary for secretion of the pollination drop, suggesting an intimate developmental relationship that involves complex bidirectional communication between (pre)pollen and ovule (e.g. Jin *et al*., 2012; Aderkas, Prior & Little, 2018; Breygina, Klimenko & Schekaleva, 2021; Muto *et al*., 2024). In *Ginkgo*, about six layers of nucellar cap cells degrade to leave a small cup‐like invagination into the apex of the nucellus, whereas the chamber is larger and more conical in cycads, somewhat resembling that of a trigonocarp. A further phase of nucellar degeneration occurs later in the post‐pollination ontogeny of both *Ginkgo* and cycad ovules, when the integumentary cells lining the micropyle degenerate to create an effective seal (Zhang, 2019; Muto *et al*., 2024). Unsurprisingly, proteins present in the archegonial fluid of *Cycas* ovules differ substantially from those present in the preceding pollination drop (Aderkas *et al*., 2022). The genetics underpinning this largely epigenetic phenomenon are, unsurprisingly, proving extremely difficult to untangle (e.g. Becker *et al*., 2025).

Neither *Ginkgo* nor cycads require the precision of apoptosis through lysigenous dissolution that would surely have been needed to generate the more complex pollen chamber, extended into a salpinx, that characterises the lagenocarps. We therefore infer that, crucially, the mechanisms underlying both pollen chamber formation and pollination drop secretion reached a high level of sophistication early in the evolutionary history of the seed. We further speculate that the mechanisms involved may have been co‐opted and adapted from those needed to allow spermatozoa to penetrate the nucellus in a presumed precursor to the typical lagenocarp anatomy that underpins hydrasperman reproduction *sensu* Rothwell (Rothwell, 1986; Rothwell & Scheckler, 1988; Rothwell & Serbet, 1992). The extant cycad *Zamia* (Fig. 2E) has recently been shown to project its nucellar beak through the micropyle and slightly beyond the outer surface of the integument (Zhang, 2019), presumably capturing pollen before the onset of lysigenous dissolution re‐opens the micropylar canal.

### (2) Transference (and loss) of function

The most obvious, and arguably most significant, example of transference of function in early seed evolution is the switch from the salpinx (nucellus) to the micropyle (integument) as the primary conduit linking the pollen chamber with the outside world, and through which a pollination drop was almost certainly extruded and then withdrawn once it had collected appropriate (pre)pollen. By contrast, responsibility for generating the pollination drop is likely to have remained a consequence of apoptosis in the distal regions of the nucellus.

A second, related case of transference of function occurs following successful pollination, when the role of sealing the pollen chamber is also transferred from the nucellus to the integument (Serbet & Rothwell, 1995). Specifically, in lagenocarps, a parenchymatous plug termed the central column, located at the centre of the pollen chamber floor (Figs 1 and 7A, B), is pushed upward by expansion of the megaspore into the inverted funnel of nucellar tissue that constitutes the salpinx. In trigonocarps, the role of sealing the pollen chamber is transferred to the cells lining the interior of the narrow micropyle, which degenerate to form what is effectively an organic glue (Fig. 6E). The more primitive among the cardiocarps (and their extant descendants among the cycads and ginkgos) continue to employ trigonocarp‐style sealing but for insurance they also indulge in a broadly similar style of micropylar sealing. In many seed‐species, the endotesta is thicker within the micropyle than elsewhere, presumably aiding production of the pollination drop and/or of the succeeding adhesive. Lastly, the more derived cardiocarps, including all but the most primitive conifers, abandoned pollen chamber sealing (and pollen chambers *per se*), thereby completing transference of function from the nucellus to the integument.

A third case of transference of function is the pollen tube, which was initially branched and haustorial, supplying the developing microgametophyte with nutrients derived from the nucellus of the ovule. Only later was the pollen tube co‐opted for transfer of spermatozoa to the megagametophyte, eventually losing its haustorial function and thus its branched architecture as it became fine‐tuned for gamete delivery alone. In a few extant conifers such as the pine‐relative *Tsuga*, pollen grains are able to germinate external to the ovule, the pollen tube extending towards and through the micropyle in order to enact fertilisation (Shang *et al*., 2025).

Further cases of transference or loss of function can be envisaged, albeit probably less profound in their consequences. For example, the robust integumentary extensions that project well beyond the salpinx in some lagenocarp seed‐species, such as *Salpingostoma dasu* and *Conostoma augustodunensis*, are likely to have offered increased protection against physical harm and, to some degree, against desiccation, but their function was probably lost once the integumentary lobes had undergone sufficient lateral congenital fusion to almost completely encase the nucellus, leaving only a narrow micropyle.

### (3) Heterochrony

There is little doubt that heterochrony – a temporal change in the expression of a trait between putative ancestor and putative descendant (Gould, 1977; Alberch *et al*., 1979; Rothwell, 1987; Bateman, 1994) – has been a frequent agent of seed evolution at all levels, from the most trivial to the most fundamental. The well‐documented contrasts in degree of fusion of integumentary lobes to each other and to the nucellus (Fig. 11B), and the degree to which in some seed‐species they project beyond the salpinx, probably owe their origins to subtle shifts in onset and offset during ontogeny, as do contrasts in the size of the pollen chamber relative to that of the remainder of the nucellus.

Similarly, the potential incorporation of leaf‐like structures subtending ovules to form integuments and cupules (see Section VII.5) is likely to have been encouraged by heterochronic shifts that allowed precocious expression of the ovule and/or radical shortening of internodes separating foliar organs. Sokoloff *et al*. (2015) also regarded the presumed evolutionary origin, within the seed, of cotyledons from megaphyllous leaves as an extreme form of heterochrony.

San Martin, Pozner & Di Stilio (2022) pursued a particularly instructive study of the extant gnetalean genus *Ephedra* comparing species that possessed fleshy *versus* papery seed‐cone bracts. They concluded that fleshy mucilaginous bracts reflected the retention and repurposing of features typical of young leaves, whereas the derived papery bracts exhibited a developmental trajectory of hyper‐mature leaves, showing a peramorphic shift from mature anatomy to younger developmental stages that aided *Ephedra* species occupying relatively arid habitats.

Arguably the most fertile ground for inferring heterochronic shifts lies in the precise timing of tissue patterning, programmed cell death, sexual maturation, and maternal resource allocation to the fertilised embryo sac. One extreme example is provided by the extant gnetalean *Gnetum*, in which the female gametophyte completes the entire ancestral somatic ontogeny after undergoing precocious sexual maturation, resulting in the evolution of its remarkable post‐fertilisation development of embryo‐nourishing female gametophytic tissues (Friedman & Carmichael, 1998).

### (4) Increased complexity and functional specialisation of the seed wall

It is helpful that, when an ovule is viewed outwards from its centre, the first two discrete layers of enveloping tissue encountered have unequivocal homologies with the megaspores of the preceding heterosporous pteridophytes: outside the thin megaspore membrane lies the megasporangium, now referred to as the nucellus. However, the consensus breaks down when considering the homologies of layers external to the nucellus that are often simply (perhaps simplistically) aggregated under the term ‘integument’ – a tissue referred to in gymnosperms as the testa. The most famous homology debate concerns the (again, over‐simplified) truism that gymnosperms have only a single integument whereas angiosperms have both inner and outer integuments (Rudall, 2021; Scutt, 2021; Shi *et al*., 2021; Herting & Stützel, 2022). There are in fact several interesting exceptions to this rule, such as the extant gnetalean *Ephedra*, an undisputed gymnosperm that possesses either two or three integuments, depending on the interpretation preferred by the observer (*cf*. Doyle, 1996; Rudall, 2021; San Martin *et al*., 2022; Zumajo‐Cardona & Ambrose, 2022).

But even for the early fossil gymnosperms that are our present focus, opposing views exist regarding how the comparatively uniform testa of the lagenocarps and trigonocarps evolved into the two distinct layers that characterise the cardiocarps, specifically a dense inner sclerotesta transitioning into an outer sarcotesta that is more malleable, both physically and evolutionarily (Figs 6A–D and 12A). Most observers perceive the sarcotesta as originating *sui generis* as a specialisation within the original undifferentiated testa (in effect, homologised with the entire lagenocarp testa). However, an alternative, palaeobotanically inspired interpretation regards the sarcotesta as an additional layer co‐opted from other lateral organs, potentially originating in broadly the same way as the original testa (e.g. Rudall, 2021). The evolutionary advantages of possessing three distinct layers of surrounding tissue, each of which can become specialised for one or more of the many functions that nature demands of a seed, are obvious. But how did they arise? The short answer to this question is congenital synorganisation (fusion) – integration of multiple organs from their inception onward with concomitant loss of previously obvious organ boundaries. Admittedly, the parenchymatous endotesta and sarcotesta of fossil seeds are differentiated primarily by noting that they reside on either side of the tougher sclerenchymatous sclerotesta, so a synorganisational origin would require loss of multiple cuticular layers from the ancestral laminar organs. This is one area of investigation where two‐dimensional adpression fossils might prove more informative than their anatomically preserved equivalents; it is a mode of preservation that brings cuticular layers into starker relief (e.g. Cleal *et al*., 2009).

### (5) Vegetative congenital synorganisation

#### (a) Viewed through the lens of telome theory

Within palaeobotanical circles at least, no paradigm has been more influential on morphological perspectives on evolution than Zimmermann's (1930, 1952, 1966) telome theory (Wilson, 1953; Smith, 1964; Stewart, 1964; Kenrick, 2002; Stein & Boyer, 2006). As a summary of how organs can diversify, it offers an elegant simplicity that translates into an associated explicit terminology. In order to appreciate the implications of telome theory, it is necessary to begin with a model vascular plant considerably more primitive than a seed‐plant, as follows.

Take one poorly differentiated truss of isotomously branching axes, termed a telome. If it terminates in one or more sporangia, regard it as a fertile telome. If the apical meristems of adjacent branches differ in size, one branch will outgrow the other in a process (overtopping) that leads to the distinction between primary branches and lateral branches. A branched lateral truss, first planated into two dimensions and then webbed through lateral fusion of the by now dorsiventrally flattened lateral branches, forms a megaphyllous leaf. (Unfortunately, Zimmermann termed this fusion process syngenesis – a term used widely today for the formation of a zygote from the union of egg and sperm; we therefore prefer to use ‘synorganisation’.) A distinction between primary and lateral branches is needed to establish the basis of plants that possess the flexibility of combining determinate and indeterminate growth in their aerial stems. A key aspect of this process was gaining the ability to express sporangia – borne either singly or more often clustered as synangia – as lateral rather than terminal organs. Vegetative leaves and reproductive organs were now free to undergo axial (longitudinal) differentiation, developing as serial homologues along the axis. This process freed the lateral organs for further evolutionary–developmental differentiation (for example, of megaphyllous leaves into bracts) and – crucially for our understanding of early seeds – it also offered opportunities for those lateral organs themselves to become subjected to further synorganisation events.

It is this complex process that is traditionally considered to have led to the formation of the integument in lagenocarps (Walton, 1953; Camp & Hubbard, 1963; Herr, 1995). Shortening of the distances separating branch points in a telome, including that of the pedicel subtending the megasporangium, is sufficient to generate the morphology of the reproductive trusses observed among the earliest seed‐plants, such as the Late Devonian *Elkinsia* (Rothwell & Scheckler, 1988; Rothwell, Scheckler & Gillespie, 1989), *Moresnetia* (Fairon‐Demaret & Scheckler, 1987) and *Archaeosperma* (Pettitt & Beck, 1968). It interests us that all three of these examples are similar in having several robust integumentary lobes surrounding, and projecting beyond, the megasporangium, albeit differing in degree of lateral fusion. And in each case, few ovules develop with varying degrees of asymmetry within an approximate ring of structures that are similar in architecture and (where known) in anatomy to the integumentary lobes – bifid structures that resemble lobster's claws and are difficult to categorise as either axis or leaf, being neither terete nor thinly laminar. They broadly resemble the integumentary lobes but are considerably larger. Collectively, these sterile lobes surrounding the ovules are generally accepted as a primitive form of cupule, although they are far less laterally fused and inrolled than the more compact among the Mississippian lagenostomalean cupules, such as *Pullaritheca* (Long, 1977b; Rothwell & Wight, 1989) (Fig. 7G) and the larger ‘megacupule’ *Calathospermum* (Walton, 1949; Barnard & Long, 1973; Long, 1975). Hence, they offer considerably less protection to the ovules within – an interpretation that also applies to the Late Devonian compression fossil *Pseudosporogenites hallei*, which appears to place a single lagenocarp seed within a shallow but fully laterally fused cupule (Prestianni, Hilton & Cressler, 2013).

Given their similar anatomical constructions and tissue organisations, it is universally believed that synorganisation of lagenocarp integumentary lobes led to the integument morphology of the trigonocarps and cardiocarps – profoundly congenitally fused, both laterally and to the nucellus within. It is therefore understandable that some observers (e.g. Camp & Hubbard, 1963) have speculated that the division of the cardiocarp testa into distinct sclerotesta and sarcotesta layers could represent a repetition of the same process of compression combined with fusion, with the cupule lobes fusing both laterally and inwardly to form the cardiocarp sarcotesta after the contents of the cupule had been reduced to a single seed. Similar hypotheses have been advanced for the trigonocarps (Meyen, 1984) and for extant cycads (Stopes, 1905) (Fig. 6D). Long (1966, 1984) conceived an alternative, and even more radical, scenario in which the lagenocarp cupule lobes fused together to form the carpels that reliably enclose the seeds of angiosperms. Given that most recent molecular phylogenies place angiosperms as sister to all extant gymnosperm lineages, it is a hypothesis that might experience a late‐stage renaissance.

What most interests us is that the integumentary lobes and cupule lobes resemble each other strongly, differing mainly in the larger size of the latter. The impression gained is one of a developmental programme of progressively shorter internodes linking axes that became increasingly leaf‐like (i.e. less terete, more prone to lateral fusion) – a shared developmental programme that is expressed at a smaller scale, and arguably to a greater extent, in the integument relative to the cupule. A scenario of sequential evolutionary origins through essentially the same process seems likely for first the integumentary lobes and then the cupular lobes. If this hypothesis of repetitive origins of enclosing organs is correct, it further strengthens belief in their telomic origin relative to the competing hypotheses of an origin through lateral expansion of the subtending pedicel/funiculus (Meeuse, 1966; Herr, 1995) or sterilisation of a surrounding ring of sporangia (Benson, 1904; Kenrick & Crane, 1997) (reviewed by Worsdell, 1904; Herr, 1995; Brenner & Stevenson, 2006; Zumajo‐Cardona *et al*., 2021).

#### (b) Viewed through the lens of evo‐devo

Before developing this argument further, we will again briefly consider plants more primitive than seed‐plants. Heterosporous pteridophytes that produce through meiosis at least one tetragonal tetrad of megaspores reliably possess spores that are near‐spherical and enclosed by a spore wall that is of approximately equal thickness throughout other than proximal to the suture. However, once three of the four meiotic products become routinely aborted (monomegaspory *sensu* Bateman & DiMichele, 1994), and the remaining functional megaspore is retained within the megasporangium for dispersal (endomegasporangy), it develops a proximal–distal polarity relative to the subtending axis and the previously spherical shape becomes at least moderately elongated parallel to that axis.

This elongation surely represents a proximal–distal cline in plant hormones that has presumably been facilitated by increased intimacy between the single remaining megaspore and the surrounding and subtending tissues (e.g. Munné‐Bosch, 2025). We regard this developmental cline as an essential pre‐adaptation to evolutionary innovations subsequently acquired by the seed, not least the acquisition of one or more integumentary layers and the ability to localise particular features and functions proximally (e.g. the chalaza), distally (e.g. the pollen chamber and archegonia) or laterally (e.g. ribs, wings). Research has, for example, demonstrated in seeds of the extant ‘model’ angiosperm *Arabidopsis* a distal auxin maximum (Cucinotta, Colombo & Roig‐Villanova, 2014; Sehra & Franks, 2015; Lora, Laux & Hormaza, 2019) that promotes cell division in opposition to cytokinins. The resulting proximal‐to‐distal cline polarises primordia and is likely to provide a physiological environment that is conducive to differential developmental patterning in contrasting regions of the ovule. Moreover, the small size and elliptical shape of early ovules probably left patterning vulnerable to local perturbations within specific tissues (Mathews & Kramer, 2012; Rudall, 2021).

Once the ovule is perceived as (*a*) the developmental product of a proximal–distal hormonal cline and (*b*) the evolutionary product of multiple rounds of synorganisation of lateral organs, it becomes far easier to accept the controversial argument that, in terms of homology, the seed is actually a meristematic axis bearing integument(s) as lateral determinate organs (Singh, 1978; Gross‐Hardt, Lenhard & Laux, 2002; Mathews & Kramer, 2012; Rudall, 2021).

Ovules of a wide range of extant seed‐plants have been shown to possess genetic properties of meristems, suggesting derivation of integumentary structures from nucellar meristems. In particular, *WUSCHEL* expression tends to be concentrated in the apex of the developing ovule primordium, and reliably initiates before the lateral integuments, mirroring the hierarchical relationship between apical meristems and leaf primordia. Development dictated by an apical meristem would help to explain the apparently constant ‘apical pull’ exerted on lateral organs by the megasporangium that, aided by internodal compression, allowed the origin and subsequent increases in complexity of the ovule. But also present in extant gymnosperms are multiple copies of *YABBY* genes that supposedly determine outer (i.e. non‐gymnospermous) integuments in angiosperms, operating alongside multiple copies of gender‐specifying gene families such as *AGAMOUS* (e.g. Scutt, 2021). Such observations led Mathews & Kramer (2012) to conclude that seeds emerged as a result of the fusion of the megasporangial and shoot developmental programmes. This insight is of fundamental importance; as neatly summarised by Rudall (2021, p. 960), ‘it rejects the traditional paradigm of seed‐plant evolution as an incremental accumulation of adaptive innovations in favour of a more labile genetic context’.

Multiple research projects are currently exploring several of the most relevant gene families in a wide range of extant gymnosperms, and are experimenting with ancestral sequence reconstruction of these genes in the hope of inferring the nature, and underlying mechanism(s), of the key phenotypic transition(s) that led to the first angiosperm flower (e.g. Murat *et al*., 2017). Interest in this topic is increasingly focused on whole‐genome duplication – specifically, the epsilon duplication event that is widely believed to have occurred at the base of the extant angiosperms (Zwaenepoel & Van der Peer, 2019; Scutt, 2021; Stull *et al*., 2021; McKibben, Finch & Barker, 2024). If successful, it would then be intriguing to apply these analytical approaches earlier in land‐plant phylogeny, aiming to isolate the morphological transition that generated the first seed. Meanwhile, palaeobotanists are actively seeking potential anatomical proxies for genome size, such as stomatal cell size (Beaulieu *et al*., 2007; Lomax *et al*., 2014), partly with the aim of identifying potential whole‐genome duplications captured in the fossil record. Stomatal cell size was recently shown to yield more accurate results than (pre)pollen size (Jardine, Morck & Lomax, 2025).

#### (c) Phenotypic transition series

The dangers of over‐literal interpretation of supposed transition series are well illustrated by the suite of anatomically preserved Mississippian lagenocarps that were so ably described and compared by Long (e.g. Long, 1960, 1960, 1960) but were then arranged diagrammatically into a classic sequence of increasing nucellar–integumentary fusion by Andrews (1963) – a sequence often reproduced thereafter (e.g. Stewart, 1983; Anderson *et al*., 2007). Specifically, the deeply divided integumentary lobes of *Genomosperma kidstonii* are shown progressing through the moderately divided lobes of *G. latens* and the shallowly divided lobes of *Eurystoma angulare* to the unlobed cylindrical integument of *Stamnostoma huttonense*, capable of circumscribing a crude micropyle. But recently, *Genomosperma latens* has been shown simply to be an earlier developmental stage of seeds formerly assigned in maturity to *G*. ‘*kidstonii*’ (Meade *et al*., 2021) (Fig. 2A), while in our tree, it is *Eurystoma* rather than *Stamnostoma* that is resolved as the potential ‘stepping‐stone’ seed‐species linking the lagenocarps to the trigonocarps (Fig. 3). Evolution proceeds more often through noisy apomorphic trends than through nice neat linear compulsions (Fig. 11B).

#### (d) Preovules versus ovules, precupules versus cupules

According to consensus definitions, a seed is a fertilised ovule, and an ovule is an indehiscent integumented megasporangium. Yet in practice, the early‐evolving lagenocarps are often referred to as ‘preovules’, defined as such by possessing integuments that do not wholly enclose the nucellus (e.g. Rothwell & Wight, 1989; Bateman & DiMichele, 1994; Gerrienne *et al*., 2004; Hilton & Bateman, 2006; Linkies *et al*., 2010; McLoughlin, 2020). Read further and you are told that being ‘completely enclosed’ actually means ‘almost completely enclosed’; an exception must be made for the micropyle – the pore through the integument that is essential to permit pollination *via* withdrawal of the pollination drop. Yet some lagenocarps approach the condition found in trigonocarps, exhibiting fully fused integumentary lobes that consequently circumscribe a micropyle. Admittedly, micropyles of some lagenocarps (*Lagenostoma*, *Stamnostoma*, *Eosperma*) are larger in relative diameter than those of trigonocarps, but others are comparable in size (notably *Conostoma*). Thus, as previously conceived, preovules are actually defined less by incomplete integumentary fusion and the consequent absence of a micropyle than by the presence of the core features of hydrasperman reproduction: a domed pollen chamber furnished with a salpinx and a central column to seal the chamber post‐pollination (Figs 1 and 7A–C). Complete enclosure can also be compromised by integumentary commissures, which constitute zones of weakness and in some cases leave the nucellus partly exposed. As noted by T. Stützel (personal communication, 2025), the evolutionary trajectory of the pollen chamber places greater functional emphasis on the transition from a dry to a wet (lysigenous) opening mechanism than on the transition from dehiscence to indehiscence.

Subtending the ovules of many of these seed‐species are cupules which, as discussed in Section VII.5.*a*, show a similarly wide range of degrees of fusion among adjacent lobes that are again hypothesised to be derived from telomes. Yet we are hesitant to advocate describing the less‐condensed examples of these organs as ‘precupules’.

Finally, we note that the likely pollination process, operating through pollination drops, appears to have passed seamlessly from lagenocarps to trigonocarps, the responsibility for this process simply having been transferred from the salpinx to the micropyle. Given that this evolutionary step appears to us rather less profound than the origin of the invaluable, multifunctional integument, we are inclined to adhere to the literal definition of the term ‘ovule’ that introduced this paragraph – an indehiscent integumented megasporangium. Preovule is a term that would be better reserved for megagametophytic structures that lack a *bona fide* integument but possess advanced heterosporous features (notably, retention and dispersal within the megasporangium of a single functional megaspore; Bateman & DiMichele, 1994).

We feel obliged to conclude this subsection by explaining the exclusion from our morphological matrix of two enigmatic potential seed precursors considered to have lacked a pollen chamber, thus potentially fitting our revised concept of a preovule: the Givetian compression fossil *Runcaria* (Gerrienne *et al*., 2004; Meyer‐Berthaud, Gerriene & Prestianni, 2018) and the Tournaisian anatomically preserved *Coumiasperma* (Galtier & Rowe, 1989, 1991).

*Runcaria* ante‐dates unequivocal seeds such as *Elkinsia* by *c*. 20 Myr. It is a two‐dimensionally preserved compression fossil, thus preventing its inclusion in our matrix of exclusively three‐dimensionally preserved seed‐species. A putative megasporangium 1.5 mm in length, assumed to contain a megaspore, subtends a much longer (*c*. 5 mm) phallic extension that in some specimens terminates in a slightly swollen apex. This structure resembles an unusually elongate salpinx, but Gerrienne *et al*. (2004, p. 858) considered its apex to be closed rather than open; they also regarded as absent the subtending pollen chamber typical of lagenocarps. They therefore in effect analogised this extended core structure with an angiosperm style and stigma, arguing that its apex trapped airborne pollen which then induced ‘dissolution of sporangial cells to allow fertilisation’ (p. 858). In the almost certain absence of a pollen tube, we are unable to visualise how the spermatozoids/ antherozoids could successfully burrow through a 5 mm‐long column of tissue to reach the archegonia, unless the archegonia were located in the swollen tip – improbably far from the oval body assumed to represent the megaspore [a conundrum previously noted by Gerrienne, Meyer‐Berthaud & Fairon‐Demaret (2005) and Meyer‐Berthaud *et al*. (2018)]. This ambiguous structure is surrounded by, and slightly exceeds, numerous filiform lobes that lack lateral fusion but nonetheless have been interpreted as integumentary. Closely surrounding these filiform structures are a smaller number of more leaf‐like organs featuring bifid apices, considered collectively to constitute a loose cupule; if so, it suggests a remarkably early origin of the (pre)cupule. Gerrienne & Meyer‐Berthaud (2007) argued that *Runcaria* constitutes a ‘proto‐ovule’, more primitive than the traditional concept of a preovule. But other observers (including ourselves) question the presence of a single functional megaspore, its indehiscent status within the sporangium, and even the status of the source plant as a vascular land plant.

By contrast, *Coumiasperma* is anatomically preserved and was provisionally scored for our matrix. Unfortunately, it is known as only a single isolated specimen, which means that we do not know whether it was cupulate. More crucially, we do not know which developmental stage it had reached, nor whether this particular seed was a typical product of the Mississippian plant that bore it. These factors matter because *Coumiasperma* appears to show a typical lagenocarp architecture except in one critical regard – the salpinx is absent and the apical region, normally differentiated through apoptosis into a pollen chamber, is instead filled with apparently undifferentiated parenchyma (Galtier & Rowe, 1989, 1991). Circumstantial evidence against immaturity is provided by its comparatively large absolute size (6.5 mm long) and the relative sizes of the megaspore *versus* the integumentary lobes. Maturity is also indicated by the presence of a cellular megagametophyte. An alternative explanation is that this particular seed is a developmental anomaly in which apoptosis failed, presumably because the required hormonal cline was insufficiently strong (Bateman & DiMichele, 2002). Teratology has been demonstrated in the Tournaisian lagenocarp *Hydrasperma*, as found *in situ* within numerous *Pullaritheca* cupules at Oxroad Bay, Scotland (this seed‐genus is located close to the base of our tree; Fig. 3). The placenta of one teratological cupule produced a cline of variation leading from well‐formed, juvenile ovules through chimaeric intermediate structures to fairly well‐formed microsporangia that would normally be borne as synangial trusses on a separate ‘male’ frond (Long, 1977b; Bateman & DiMichele, 2002).

Whatever its true nature, arguing that *Coumiasperma* ‘represents … fossil evidence of an evolutionary stage between pteridophytic and gymnospermous reproduction’ but also ‘could be interpreted as a very primitive gymnosperm which contradicts current theories of monophyletic gymnosperm origins’ (Galtier & Rowe, 1989, p. 225) seems ambitious. Missing only one lagenocarp feature, it seems far more likely that, if it is a genuine product of evolution, *Coumiasperma* would either have originated immediately prior to conventional lagenocarps or lost its salpinx through paedomorphosis – presumably as an adaptation to the aquatic environment and water‐borne pollination envisaged by Galtier & Rowe (1989). In either case, it would not challenge the widely accepted monophyly of seed‐plants.

## VIII. PALAEOECOLOGY

### (1) Seed/cupule dispersal

When herbivory threatens their seeds, plants are capable of evolving two ostensibly opposite responses: deterrence or encouragement. Deterrence is the obvious, and earlier evolved, strategy, operating either through physical or chemical means. Physical deterrents include a robust, resilient testa and dense epidermal trichomes, whereas chemical deterrents include toxins, resins and adhesives (e.g. Kesseler & Stuppy, 2006). At least one of our study species, *Hirsutisperma rothwellii* (Scott *et al*., 2019), apparently combines the physical and chemical approaches to deterrence by producing abundant glandular hairs. But equally, an argument could be made that the dense glandular hairs were an adaptation that encouraged positive rather than negative interactions between plants and vertebrates, potentially gluing seeds to the skin of vertebrates as a means of permitting longer‐distance dispersal. However, there is no evidence that any of the seed‐species developed trichomes or other larger‐scale epidermal projections that ended in hooks of the kind often seen in extant angiosperms, perhaps because hooks function better on the fur of mammals (a group entirely absent from the Palaeozoic) than on the scales of reptiles or the oily skin of amphibians.

The case for diverse approaches to arthropod herbivory has been well made (Scott, Stephenson & Chaloner, 1992; Scott *et al*., 2019; Labandeira *et al*., 2007; Labandeira, 2019; Santos, Wappler & McLoughlin, 2024), but could Palaeozoic seeds also have been distributed inside vertebrates (Reisz & Sues, 2000; Tiffney, 2004)? There are obvious similarities between trigonocarps and the seeds of extant cycads and *Ginkgo*, all sharing division of the integumentary testa into two comparatively thick layers: a hard resistant sclerotesta within a softer, fleshier sarcostesta, which is often brightly coloured and encourages consumption by vertebrates as a dispersal mechanism. Considerable diversity in thickness, texture and colour is evident, even within a single genus (e.g. the extant *Cycas*; Dehgan & Yuen, 1983). A similar mechanism could in theory have been employed by these early seeds, as they were coeval with fish, early amphibians and (from the early Mississippian onwards) with the earliest reptiles (e.g. DiMichele & Hook, 1992; DiMichele, Phillips & Pferfferkorn, 2006; DiMichele *et al*., 2023). The less‐differentiated testa of all lagenocarps, coupled with the relatively small size of most, offer less opportunity to evolve fleshiness. Nonetheless, their testa more closely resembles a sarcotesta than a sclerotesta, suggesting that none of the seed‐species in either group is likely to have become desiccated prior to dispersal (unfortunately, in fossil seeds it is impossible to distinguish loss of the external sarcotesta through programmed developmental processes from loss through *post‐mortem* decay). The exposed nucellus of lagenocarps is unlikely to have offered the embryo much protection against the digestive enzymes of vertebrate herbivores, and even for trigonocarps and cardiocarps, the micropyle represents an obvious point of weakness. This weakness is further exacerbated in the case of seeds that possess a dissected commissure, such as the trigonocarp seed‐genus *Pachytesta* (e.g. Taylor, 1965) and a minority of seed‐species scattered widely across the cardiocarp clade.

It seems likely that most Palaeozoic seed‐plants relied primarily on abiotic dispersal through wind or water. The estimated weight: volume ratio of many Palaeozoic seeds suggests that they likely would have floated on water, possibly facilitated by internal air chambers apparently present at the base of the nucellus in some trigonocarps (Combourieu & Galtier, 1985). Flotation may also have been aided by external features – at the larger scale by wings or ribs, and at the smaller scale by trichomes. Lagenocarps, trigonocarps and cardiocarps all show repeated evolutionary origins of expanded wings (most commonly two in lagenocarps and cardiocarps, but three or four in trigonocarps) that would have significantly increased the distribution radius around the parent tree through gravity alone, and would have further expanded that radius when wind‐assisted. Substantial wings first evolved in the Late Devonian, soon after the origin of the seed habit (Rowe, 1992, 1997). Nonetheless, we suspect that both biotic and abiotic dispersal mechanisms were inefficient. Combined with rudimentary dormancy, this means that the vast majority of Palaeozoic seeds were probably comparatively ineffective disseminules, typically germinating close to their parent plant.

### (2) Seed size

**Fig. 13:**
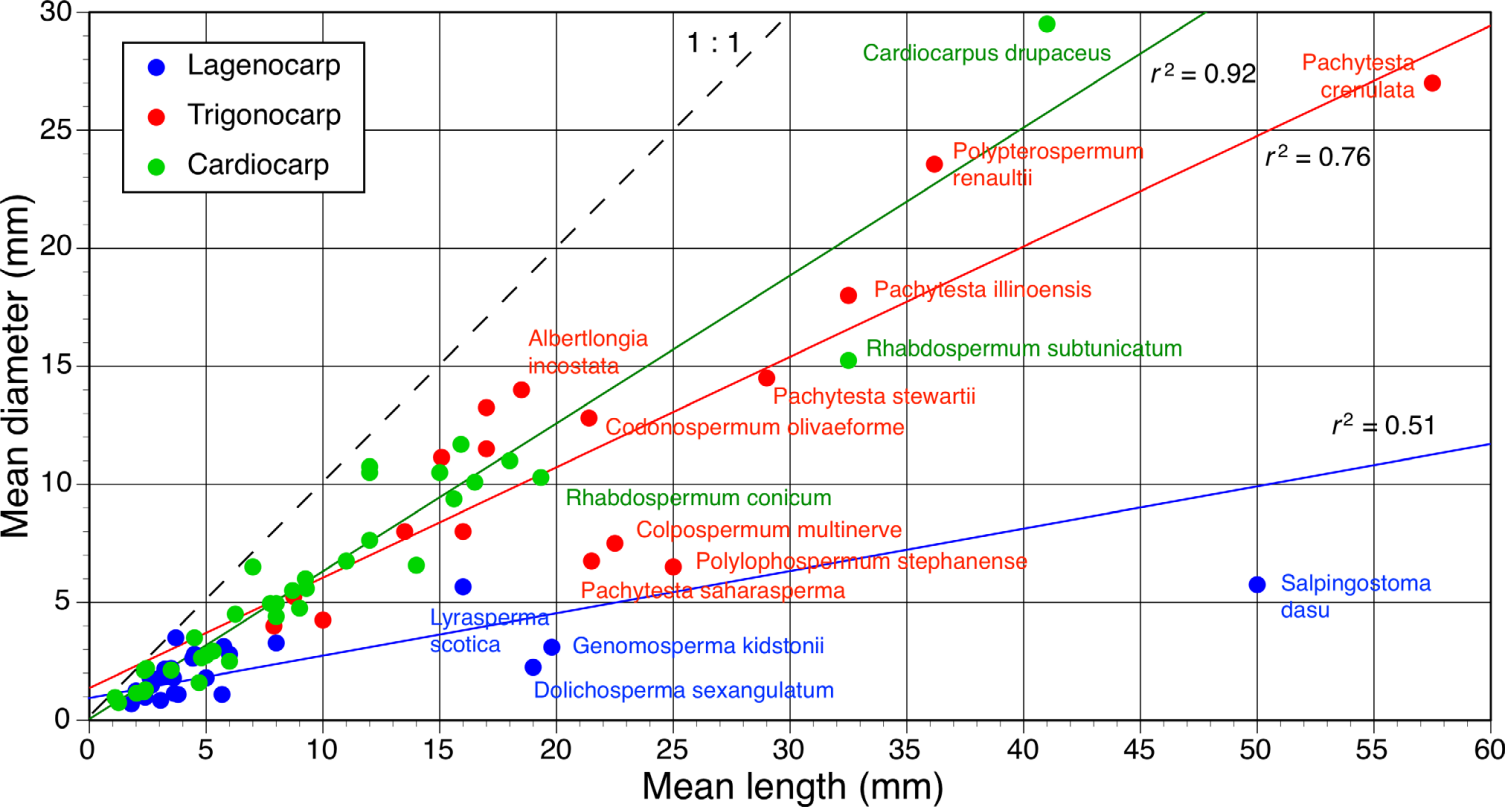
Plot of mean seed length *versus* mean diameter for 77 Palaeozoic seed‐species (usable data could not be obtained from *Stamnostoma oliveri* and *Cyclospermum tenue*). Separate regressions are presented for lagenocarps, trigonocarps and cardiocarps. Plotted using Deltagraph v7.1 (SPSS/Red Rock software 2013).

Although Sims (2012) argued that the Palaeozoic seed record is probably biased in favour of the larger among the seeds present at that time, we believe that our decision to ignore adpressed seeds and instead confine this study to anatomically preserved specimens is likely to have yielded a more representative size spectrum (Fig. 13). Three‐dimensional plant fossils are commonly studied through serial sectioning, serial acetate peeling or, more recently, X‐ray microtomography. Within the relevant rock specimens, each of these approaches can detect all seeds present, irrespective of their sizes.

Our approach to measuring Palaeozoic ovules involved averaging lengths with and without external ornamentation and diameters across the longest and shortest distances, again both with and without external ornamentation (Fig. 13). In terms of overall size, all would have considerably exceeded the 5 μg dry mass calculated as the minimum size for a viable seed by Raven (1999). There is little overlap between the smaller lagenocarps (all but two of our study species being less than 3.5 mm in mean diameter) and the larger trigonocarps (all but three exceeding 6.5 mm in mean diameter). Given that all of the Devonian and Mississippian ovules measured by us were lagenocarps (Fig. 4), it is unsurprising that values for both mean and maximum seed size increased through the late Devonian and Carboniferous. However, a few unusually large seeds emerged relatively soon after the origin of the seed habit, notably *Salpingostoma dasu*, which had reached *c*. 50 × 6 mm by the Tournaisian (Gordon, 1941). The cardiocarps span the combined size range of the lagenocarps and trigonocarps. Comparing the taxonomic groups within the cardiocarp category reveals that most cordaite ovules exceed in size seeds produced by callistophytes, glossopterids and primitive conifers (Figs 3 and 13). In terms of shape, all seeds are broadly ovate in shape and longer than wide (Fig. 13). Both the trigonocarps and cardiocarps yielded similar well‐supported regressions around a length: width ratio approximating 3:2, whereas the regression for lagenocarps was weaker and approximated 5:1, influenced by large‐seeded species possessing robust integumentary lobes that extend well beyond the salpinx (*Genomosperma kidstonii*, *Dolichosperma sextangulatum*, and especially *Salpingostoma dasu*).

It is unfortunate, at least for our purposes, that the vast comparative data sets available on seed size in extant species are routinely based on seed mass, rather than on the linear dimensions to which palaeobotanists are inevitably restricted (*cf*. Mazer, 1990; Sims, 2012); this contrast in methodology limits our ability to make direct comparisons between the dead and the living. Among extant taxa, seed mass is both a good proxy for the energy invested in producing the seed and a strong determinant of dispersal syndrome (Moles *et al*., 2005). In this context, average seed mass diminishes greatly with increasing distance from the equator (Moles *et al*., 2007). Large seeds are especially advantageous when attempting to germinate under low light conditions (e.g. Linkies *et al*., 2010), suggesting that the increase in size and light‐capturing ability of forest trees that evolved in several lineages through the Late Devonian and Carboniferous (e.g. DiMichele *et al*., 2006, 2023) is likely to have helped drive the coeval progressive increase in maximum and average seed size. However, the larger among the Pennsylvanian trigonocarps and cardiocarps match in size the larger‐seeded taxa of extant cycads (Whitelock, 2002), suggesting that there has existed an upper limit to viable seed size throughout the evolutionary history of the gymnosperms (Sims, 2012) – a limit that could be broken only by the later advent of the angiosperms. This apparent constraint most likely reflects evolutionary trade‐offs in maintaining acceptable levels of physical robustness, dispersibility and resource allocation to the subsequent juvenile plant.

### (3) Habitats and preservational environments

#### (a) Taphonomy and preservation

Before attempting to infer the preferred habitats of seed‐bearing plants through the Palaeozoic, it is important to note that the fossil record has passed through a severe taphonomic filter. Specifically, most plant fossils are transported from their source communities to environments more conducive to preservation, typically either in water or through mass‐flow events. Seeds are sufficiently small to risk becoming sorted into contrasting size/weight classes during transport (e.g. Mazer, 1990; Chambert & James, 2009), sometimes forming strong concentrations. For the purpose of habitat inference at least, foliar megafossils may in some circumstances be more reliable than seeds (DiMichele *et al*., 2006).

A particularly unusual set of circumstances is needed in order to preserve the three‐dimensional anatomy of fossil plants, requiring rapid permeation of appropriate mineral‐rich fluids throughout the deposited plant organs. Three materials were each responsible for preserving *c*. 30% of the 79 seed‐species included in Fig. 3: silica (Fig. 6B, E), bedded calcium (or calcium–magnesium) carbonate (Fig. 2D) and nodular calcium carbonate (Fig. 7C). A smaller proportion of the seed‐species had been preserved (generally less successfully) in iron carbonate or iron sulphide. In a few cases, the fossil seeds had been charcoalified by wildfires or volcanic activity prior to mineralisation (Figs 2B, G and 7C). In terms of preservational environments, volcanic terrains and peat swamps (often peri‐oceanic) figure most prominently, the latter consisting of highly fossiliferous coal balls – calcite nodules formed within certain coal‐forming peats of the Pennsylvanian and early Permian (Figs 2C and 7D). A few of the seeds were preserved in lakes, lagoons or shallow seas that developed appropriate water chemistries.

Given the unusual nature of the preservational habitats reported here, considerable bias inevitably affects our perceptions of floristic composition. Nonetheless, especially when they are anatomically preserved and allowance is made for the possible presence of multiple ontogenetic stages, seeds are arguably the best single plant organ for assessing species‐level diversity of seed‐plants within these communities (Bateman, 1991). Although leaf and spore/pollen assemblages often prove more diverse when summarised at the organ‐species level, their greater potential for long‐distance transport (especially in an era dominated by wind pollination) means that they are even more likely than seeds to blend components of multiple plant communities.

#### (b) Preferred habitats

Given the important contribution of plants to soil formation, early seed plants such as *Elkinsia* would have had the disadvantage of having access only to weakly developed soils, poor in oxygen and nutrients. Being small shrubs that were rarely significant components of late Devonian floras, they are regarded as primary successional plants in lowland clastic situations such as river levées and prograding deltas (e.g. Scheckler, 1986). Thus, although the evolution of the seed supposedly freed gymnosperms from dependency on free water for sperm dispersal (e.g. Chaloner, 1967; Bateman & DiMichele, 1994), in practice free water appears to have remained a requirement during the early evolutionary history of seed‐ferns.

Seed‐ferns play a greater role in Mississippian than late Devonian floras (e.g. Retallack & Dilcher, 1988), aided by an architectural radiation that permitted occupation of a much wider range of ecological niches and diversification into both precociously reproducing *r*‐strategists and larger‐bodied, woodier *K*‐strategists (*sensu* MacArthur & Wilson, 1967). Species‐level diversity, in terms of both whole‐plant species and seed‐species, was greatest in mosaic habitats subject to significant levels of abiotic disturbance, such as volcanigenic landscapes (Bateman, 1991).

Much of our knowledge of Pennsylvanian seed‐plants derives from the coal‐swamp floras that occupied depositional basins in Euramerica, particularly from those providing coal‐ball preservation (DiMichele, 2014). Architectural diversification continued into vines, scramblers and slender understorey shrubs capable of forming semi‐self‐supporting thickets, collectively producing stratified forests of a structurally (if not taxonomically) modern aspect (Rothwell & Scheckler, 1988; Bateman, 1991; Rothwell & Serbet, 1992; Bateman *et al*., 1998; DiMichele *et al*., 2006; Anderson *et al*., 2007; Cleal, 2008; DiMichele, 2014). Major contributors included the medullosans, which were responsible for producing the larger, fleshier trigonocarp seeds along with correspondingly large prepollen (Dunn, Rothwell & Mapes, 2006; DiMichele *et al*., 2023). Lyginopterids and callistophytes were less ecologically prominent. However, seed‐plants continued to prefer clastic over organic soils and to act as primary colonisers, preferentially benefiting from environmental perturbations such as forest fires. Drier extra‐basinal habitats were under‐saturated with plant life, providing opportunities for evolutionary innovation in newly arisen, less desiccation‐prone seed‐plant lineages (DiMichele *et al*., 2020). Cordaites predominated in seasonally arid habitats during the early and mid‐Pennsylvanian but in the late Pennsylvanian they were succeeded by early members of the conifer lineage, sometimes supported by putative cycads or cycad precursors.

**Fig. 14:**
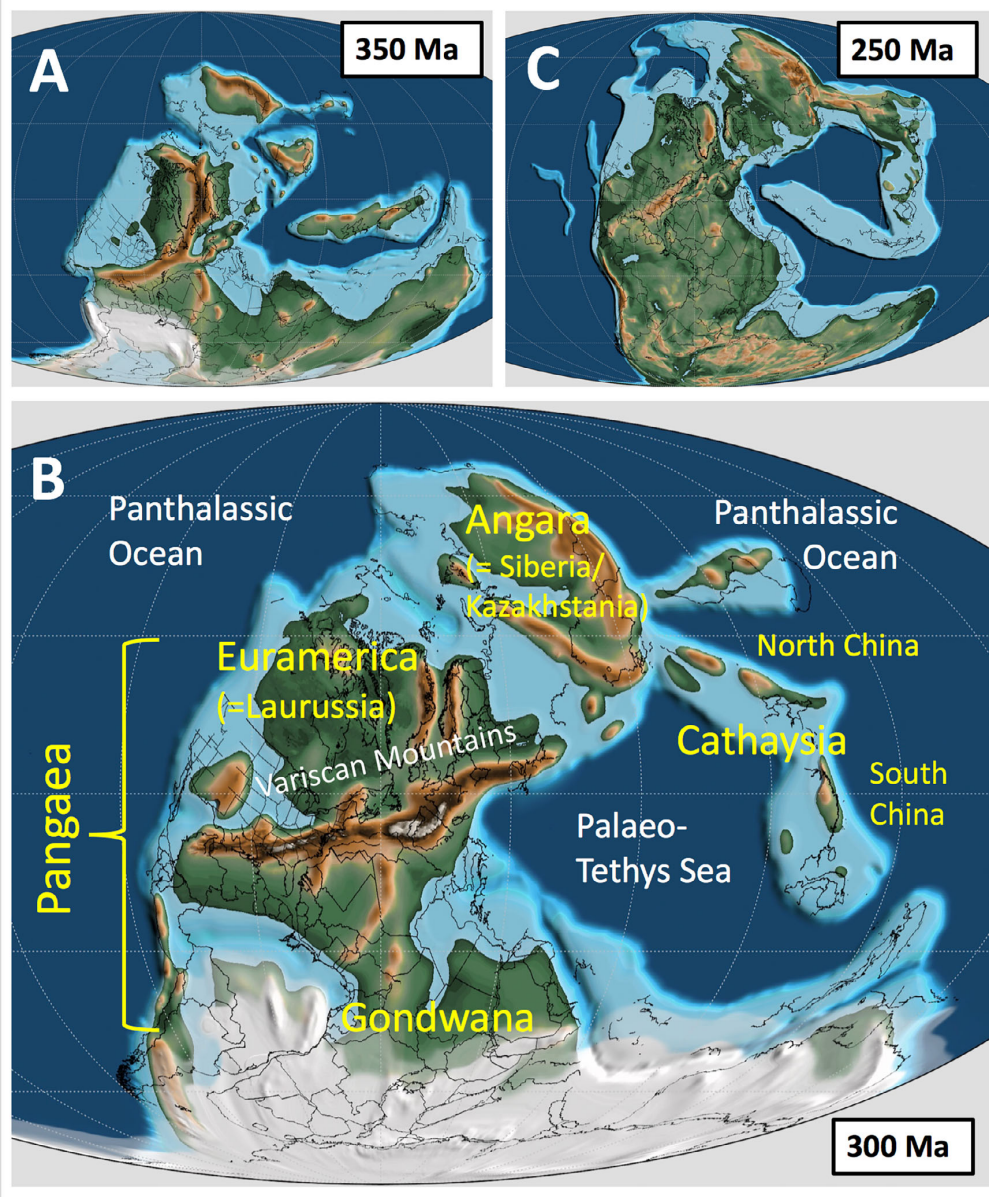
Palaeogeographic reconstructions for the late Tournaisian (A, 350 Ma), late Moscovian (B, 300 Ma), with major features labelled, and end‐Permian (C, 250 Ma). Base maps cropped from fig. 14 of Scotese (2021). Reproduced through pemission of the Licensor through CopyrightClearanceCenter.

It was these and other related groups of seed plants that apparently rose to ecological dominance in the more seasonal and drier climates that increasingly dominated Pangaea through the Permian, along with derived seed‐ferns such as callipterids and peltasperms. Determining the precise timing and scale of these perceived floristic changes is made difficult in that wetland plants were not only far more likely to be preserved but also far more likely to be preserved well, thus preferentially attracting the attention of Palaeozoic palaeobotanists (DiMichele *et al*., 2020). Certainly, by the mid‐Permian, specialised arid biomes had formed from lineages that have left extant descendants, dominated by conifers with subordinate cycads and ginkgos (Rees *et al*., 1999). However, further east in Cathaysia (Fig. 14), the wetland seed‐plant lineages persisted through the early Permian in North China, subsequently benefitting from more humid climates that persisted even into the late Permian in the comparatively palaeogeographically isolated South China fragment of continental crust (Hilton & Cleal, 2007; He *et al*., 2021).

### (4) Extinctions

#### (a) Global change through the Carboniferous and Permian

The bulk of the anatomically preserved seeds included in the present analysis were found in the palaeogeographic equivalents of modern Northeast America and Northwest Europe. At the beginning of the period covered by this review (summarised in Fig. 4), the southern continent of Gondwana collided with the northern continent of Euramerica, forming a single supercontinent named Pangaea and inducing along the collision zone the Variscan–Alleghanian phase of mountain‐building, which continued until the end of the Capitanian. Pangaea drifted northward throughout the review period; for example, the British Isles were placed at *c*. 15° S during the Famennian (late Devonian: Fig. 14A), crossed the equator at the beginning of the Moscovian (Fig. 14B), and by the end of the Permian had reached *c*. 15° N (Fig. 14C). Throughout this period, northeast North America lagged *c*. 15° of latitude behind Britain (Scotese, 2021).

Thus, current evidence suggests that the seed plants originated in the southern subtropics and underwent an initial diversification in the tropics, spreading primarily northward into the subtropical zone through the Mississippian and into the north‐temperate zone through the Pennsylvanian. By the mid‐Permian, provincialism distinguished between seed‐plants of the north‐temperate Angaran Province (cordaites, peltasperms) and those of the south‐temperate Gondwanan Province (most notably glossopterids).

Considered from a climatic perspective, there was a short‐lived Southern Hemisphere glaciation at the close of the Devonian (Lakin *et al*., 2016), followed by evidence of Gondwanan glaciations in the mid‐Tournaisian and again in the late Visean. At the end of the Visean, these limited glaciers around the South Pole gave way to the Late Palaeozoic Ice Age – an oscillatory series of several more profound glacial periods separated by interglacials and consequent, dominantly eustatic, changes in sea level of up to 120 m (Montañez & Poulsen, 2013; Fielding, Frank & Birgenheier, 2023). In Euramerica, the glaciers were most extensive during the Bashkirian–early Moscovian before yielding to a period between the late Moscovian to the mid‐Gzhelian when a *c*. 1.5 Myr cyclicity of several glacials and interglacials is becoming increasingly evident as research progresses. The greater seasonality inherent in the interglacials destabilised the vegetation, periodically obliging wetland biomes to retreat into refugia (DiMichele *et al*., 2009; DiMichele, 2014; Montañez *et al*., 2016). Glaciers expanded again from the mid‐Gzelian to the early Sakmarian, before becoming heavily depleted during the Artinskian Warming Event and disappearing by the close of the Wuchiapingian (Fig. 14).

Major ecological turnovers tended to occur at a CO_2_ threshold of *c*. 500 ppm. During the Pennsylvanian glacial maximum, atmospheric composition achieved the highest ever ratio of O_2_:CO_2_. Crucially, CO_2_ levels were exceptionally low by geological standards (*c*. 200 ppm), arguably falling even below those of the modern pre‐industrial level of *c*. 280 ppm. By contrast, O_2_ levels were up to 14% higher at *c*. 35%, increasing atmospheric pressure and allowing even lush vegetation to burn furiously during wildfires that returned much CO_2_ to the atmosphere (Scott & Glasspool, 2006; Franks *et al*., 2014; Richey *et al*., 2020). Global mean annual temperature fell by about 6 °C during the early Serpukhovian, from *c*. 19 °C to a long‐term average of *c*. 13 °C, before rising fairly steadily throughout the Permian to *c*. 20 °C by its close (Scotese, 2021). The ecologically catastrophic end‐Permian event saw temperatures rise exceptionally rapidly to *c*. 32 °C, heralding geological history's most profound icehouse‐to‐hothouse transition that led inexorably to substantial reduction in terrestrial biodiversity (Xu *et al*., 2022, 2025; Hua *et al*., 2023). Given such an unstable environmental backdrop, the major biotic extinctions that occurred during this period are hardly surprising; even ‘advanced’ seed‐bearing plants proved far from immune to such radical perturbations.

#### (b) Losses of major groups of early seed‐plants

Four periods of relatively intense terrestrial extinctions are recognised in Euramerica during the late Palaeozoic, approximating the junctions of the Moscovian and Kasimovian, Gzhelian and Asselian, Capitanian and Wuchiapingian, and the end‐Changhsingian (Fig. 4). The causes of each of these periods of enhanced extinction rate remain under debate, the uncertainty heightened by circumstantial evidence that each represents a substantial, and in some cases cyclical, period of environmental change rather than a single catastrophic event of the kind inferred to have been caused by the asteroid impacts that famously delimit the Cretaceous–Palaeogene (K–P) boundary. Climatic change is typically invoked, most notably the diachronous west‐to‐east increase in seasonality that characterised much of the late Pennsylvanian and Permian. However, climate change actually represents explanation rather than causation. Likely underlying factors such as mountain‐building, changes in oceanic and/or air currents, and especially exceptional volcanic activity are frequently placed under suspicion as factors contributing to climate change, operating within a framework of regular, interacting astronomical cycles.

Before addressing the relative survivability of lagenocarps, trigonocarps and cardiocarps, we should first note that understanding the Capitanian–Wuchiapingian transition is made more difficult by the absence of anatomically preserved seeds for the 32 Myr that separate the end of the Sakmarian from the end of the Capitanian (Fig. 4). Only two of the seed‐genera included in our analysis apparently span this gap, and we view both as taxonomically ambiguous. *Cardiocarpus huopuensis* can now be discounted as a remarkable gap‐jumping ‘Lazarus’ taxon (a species that disappears from the fossil record for a considerable interval of time before reappearing), because our results (Fig. 3) clearly demonstrate that this seed‐species has in fact been taxonomically mis‐assigned to a genus that has long suffered from lacking apomorphic characters, leading to an inadequate genus‐level diagnosis (Hilton *et al*., 2003). By contrast, *Stephanospermum trunctatum* does not appear to deviate significantly from the morphology of any other species of *Stephanospermum* included in our analysis, although it is unfortunate that this is the seed‐species that yielded the least complete data set. However, despite the paucity of Lazarus taxa, the impression gained of early lineage extinction is misleading. Although lagenocarps and trigonocarps disappeared from Euramerica at the close of the Pennsylvanian, a combination of compression seeds and unpublished anatomically preserved seeds shows that further East, both lagenocarps (Hilton *et al*., 2001; Hilton & Li, 2003; He *et al*., 2021) and trigonocarps survived into the early Permian in North China. Moreover, trigonocarps extended into the late Permian in South China, eventually succumbing only to the exceptional Permian–Triassic extinction event along with the cordaite cardiocarps (Hilton & Cleal, 2007; Spencer *et al*., 2013b; McLoughlin, 2020).

The question of why first the lagenocarps and then the trigonocarps became extinct during the Palaeozoic era, whereas the cardiocarp lineage progressed to the present day, is obviously of particular interest. We admit at the outset of this particular discussion that the answers could lie in organs other than the seed, or simply in the overall physiology of the plants in question, acknowledging the increasing loss of ever‐wet habitats that most likely reflected increasing seasonal aridity. However, given that the origin of the seed is credited with liberating seed‐plants from the need for free water, and that seed‐plant groups such as the cordaites and conifers responded more positively than other land‐plant groups to increasing aridification (or, more accurately, decreasing humidification), we are inclined to consider whether there may have existed vulnerabilities in the reproductive phase of the life histories of trigonocarps and lagenocarps. The loss of both trigonocarps and lagenocarps together diachronously, first in Euramerica at the Carboniferous–Permian transition, then in North China and finally in South China at the Permian–Triassic transition (Hilton & Cleal, 2007), suggests that if these two groups did indeed possess a weakness then they shared it.

It seems almost perverse to critique the incredibly elegant (pre)pollination mechanism employed by even the earliest lagenocarps, famously termed hydrasperman reproduction by Rothwell (1986; Rothwell & Scheckler, 1988). Previous concerns regarding functionality have focused on the reliance of a single layer of tissue, the nucellus, first to capture the prepollen grains and then to protect them from herbivory, pathogens and, perhaps most crucially, desiccation. However, one of us (J. Hilton) now casts suspicion on the central column. Located at the centre of, and extending upward from, the pollen chamber floor, the central column is considered responsible for sealing the salpinx, as a result of outward expansion of the underlying megagametophyte. We note that this plug is composed entirely of parenchyma cells (albeit with comparatively thick walls) and hence was probably more vulnerable to physical damage, pathogen infection and especially desiccation than the more robust integumentary wall cells responsible for mucilagenously sealing the integumentary micropyle in trigonocarps and cardiocarps.

Both the lagenocarps and trigonocarps are judged likely to have lacked pollen tubes, meaning that vulnerable sperm were obliged to undergo the treacherous swim from the aperture of the prepollen to the archegonia of the ovule. Indeed, one recent theory (Clark *et al*., 2021), prompted by the extraordinarily large size of their prepollen grains (Taylor & Rothwell, 1982), suggests that the swim might have been a long one in the case of at least some trigonocarps. The outsized prepollen appears poorly adapted for wind pollination and has instead been regarded as circumstantial evidence that insect vectors were employed. However, it has recently been suggested (Clark *et al*., 2021) that the medullosans represent a regression to a pollination mechanism more typical of advanced heterospory (*sensu* Bateman & DiMichele, 1994), pollination potentially being enacted between prepollen and ovules abiotically as they floated together on the surface of standing water. If so, this would make trigonocarps especially vulnerable to increasing aridification (DiMichele, 2014).

Comparison with studies of plant responses to the infamous K–P asteroid impact suggests an alternative threat that could have impacted on lagenocarps, trigonocarps and possibly also the more primitive among the cardiocarps, all of which are suspected to have produced recalcitrant (desiccation‐sensitive) seeds. Specifically, the K–P extinctions induced by the ensuing ‘nuclear winter’ preferentially eliminated angiosperm groups that today are known to possess short‐lived, recalcitrant seeds (Berry & Jaganathan, 2022). The need to survive increasingly long periods of aridity during the Permian and especially into the Triassic is likely to have disadvantaged early‐divergent gymnosperm lineages, particularly as current evidence suggests that the slow recovery of plants may have limited carbon sequestration for as long as 5 Myr (Xu *et al*., 2025). Certainly, the gymnospermous lineages that diverged through the Triassic (conifers, cycads, bennettites, ginkgos) show increasing evidence of adaptations likely to aid survival during periods of substantial environmental fluctuations. They successfully made permanent the global ecological dominance of seed‐plants that had arisen gradually through the late Palaeozoic, long before the belated appearance of morphologically recognisable angiosperms.

## IX. CONCLUSIONS AND FUTURE DIRECTIONS

Having prepared a *de facto* sequence of topical essays that extend all the way from basic seed anatomy to mass extinctions and climate change, we have chosen to conclude by identifying issues discussed in successive sections of this review (Sections III., VIII.) that we regard as being of sufficient gravity to demand prioritisation for further targeted research.

### (1) Organ phylogenies

We hope that we have amply demonstrated the value of the painstaking field searches for, and subsequent descriptions of, anatomically preserved seeds that underpin the present synthesis. Such data are increasingly unfashionable in an era of fast (often recycled) data and an emphasis on scientific questions of obvious immediate benefit to humankind. The highest priority for future field surveys must surely be to bridge the 34 Myr gap in seeds of adequate preservation that characterises in the mid‐Permian (Fig. 4). We also emphasise the under‐rated value of constructing evolutionary trees from organ‐species, provided that the many risks inherent in employing such limited data are fully appreciated and that specimen description integrates modern scanning techniques such as X‐ray microtomography (Fig. 2B) with more traditional techniques involving physical sectioning (e.g. Meade *et al*., 2021).

### (2) Whole‐plant phylogenies

Similarly, the even more painstaking and increasingly unfashionable process of conceptually reconstructing whole plants (Bateman & Hilton, 2009) must continue if we are to understand better the relationships among major plant groups. It could be argued that much of this review is rooted in the simple but inspired use by Oliver & Scott (1904) of distinctive epidermal glands to correlate three isolated Pennsylvanian organ‐species and thereby circumscribe the earliest group of seed‐bearing plants, the pteridosperms. Further whole‐plant reconstructions would surely improve upon existing morphological phylogenies that combine the living and the dead to such instructive effect. Nevertheless, there remain radical discrepancies between morphological trees combining the living and the dead *versus* molecular trees employing DNA sequence data derived solely from extant species. Moreover, even within each of these two categories of analysis, there remain significant differences in resultant trees among competing studies, and a tendency to reduce fossil taxa to a small number of ‘placeholders’. In morphological analyses, such fossils fail to capture the full range of variation present in the group. In DNA‐based analyses, precisely stratigraphically dated fossils are relegated to the ignominy of inferring far less reliable dates for divergences among molecularly characterised lineages.

### (3) Homology assessment

All kinds of comparative morphological analyses are equally dependent on assertions of homology when attempting to justify the division of particular morphological features into characters for scoring or quantification. Homology recognition is one area where comparing the dead with the living becomes crucial to the goal of obtaining credible interpretations. We are fortunate that two of the three main lineages of cycads (*sensu* Coiro *et al*., 2023), one ginkgoid lineage, three disparate gnetalean lineages, and the majority of the major conifer lineages (although sadly not the earliest) have survived to the present day (Fig. 10). This review has benefited greatly from experimental observations made on extant gymnosperms, especially *Ginkgo* and the cycad genera *Cycas* and *Zamia*. Many of the evolutionary hypotheses discussed herein – synorganisation and the telome theory, transference of function, heterochrony and heterotopy – depend upon credible homology assessments that can be informed by modern descendants.

### (4) Genetics

To prevent this review expanding into a book, we have deliberately excluded discussion of the genetic structures and epigenetic processes that must ultimately underlie all of the morphological and behavioural issues that we have chosen to address in relation to gymnosperms. In recent years, great progress has been made in research areas such as evolutionary developmental genetics, but considerably less research has been conducted on the reproductive biology of gymnosperms relative to that of the angiosperms – the plant group that provides the basis of almost all of our plant‐based foods and medicines. Although the genome architectures of extant gymnosperms differ greatly from those of angiosperms (e.g. Wan *et al*., 2022), interesting results are now being obtained through reconstructing ancestral sequences and inserting the genes into extant model plants (e.g. Murat *et al*., 2017; Pont *et al*., 2019; C. Scutt, M. Morel, L. Rambaud‐Lavigne, B. Boussau, R. M. Bateman, P. J. Rudall & R. Tavares, in preparation). Also, a better understanding of whole‐genome duplications in gymnosperms (e.g. Jiao *et al*., 2011; Lomax *et al*., 2014; Scutt, 2021) could help to determine whether, as was recently theorised, duplication events played a crucial role as exaptations that gave some gymnosperm lineages the additional genomic flexibility needed to increase their survival potential through improving vital characteristics such as seed dormancy (e.g. Berry & Janganathan, 2022).

### (5) Epigenetics

Another key area where rapid progress is at last being made is epigenetics – essential knowledge if we are to understand the extraordinary complexities of how the genetic code is translated into plant development and behaviour. For example, preparing this review reinforced our prior belief that the myriad interactions between gymnosperm microgametophyte and megagametophyte – mediated initially *via* the pollination drop – are both bidirectional and extremely complex (e.g. Breygina *et al*., 2021; Muto *et al*., 2024), and presumably have been so since their inception. A cogent argument can be made for the details of this particular set of interactions constituting the origin of the seed, and thus the origin of recognisable gymnospermy.

### (6) Environmental change

One final area of increasingly profound significance is the nature and consequences of the interactions of seed plants in general – and ovules and seeds in particular – with an environment that is presently changing in so many ways, often at an ever‐increasing rate. There is considerable value in observing which morphological features in fossil lineages appear to have made them more (or less) vulnerable in the past to the kinds of rapid environmental change their descendants are experiencing today. In particular, we seek to identify the tipping points at which biotic interactions are likely to fail, potentially heralding ecosystem collapse. Reproductive biology, and the viability of the ensuing seeds, will inevitably be crucial aspects of all such studies.

## Supporting information


**Appendix S1.** Details of scored taxa.

**Appendix S2.** Details of scored characters.

**Data S1.** Morphological cladistic matrix (Excel .xlsx spreadsheet).

## Data Availability

The data that supports the findings of this study are available in the supplementary material of this article.
