# Supplementary material for: Early evolutionary history of the seed
Source: Biol Rev Camb Philos Soc. 2026 Jan 26;101(3):1511–53. doi: 10.1002/brv.70134 (PMC13149795; doi:10.1002/brv.70134)
Supplement: Supplementary file 1 — Appendix S1. Details of scored taxa. [file BRV-101-1511-s001.docx]

**Appendix S1. Details of Scored Taxa**

***Elkinsia polymorpha*** Rothwell, Scheckler & Gillespie. As described by Rothwell *et al.* (1989), Rothwell & Serbet (1992) and Serbet & Rothwell (1992). Specimens from shales in the Upper Hampshire Formation at Elkins, West Virginia (Rothwell *et al.*, 1989; Rothwell & Serbet, 1992), dated to the Fa2c Biozone, Famennian, late Devonian (Rothwell *et al.*, 1989). Cupulate ovules of the hydrasperman pteridosperm of the same name.

***Kerryia mattenii*** Rothwell & Wight. Cupulate ovules as described by Matten *et al.* (1980) and Rothwell & Wight (1989) bearing ovules of *Hydrasperma* *tenuis* (Long, 1961*b*; Rothwell & Wight, 1989). Megagametophytes as documented by Matten *et al.* (1984). Specimens from the Kilmore Sandstone Formation at Ballyheigue, Ireland. Stratigraphically dated to the LE miospore biozone (Klavins & Matten, 1996) of latest Famennian (Devonian) (Klavins & Matten, 1996). Ovulate structures of the early pteridosperm *Laceya hibernica* (Matten, 1992; Hilton & Bateman, 2006).

***Pullaritheca longii*** Rothwell & Wight. As described by Matten *et al.* (1980) and Rothwell & Wight (1989), with ovules of the *Hydrasperma*-type (Long, 1961*b*). Specimens from the Whiteadder river at Hutton Bridge, Berwickshire (Long, 1961*b*) and Oxroad Bay, East Lothian (Rothwell & Wight, 1989), UK. Ballagan Formation, late Tournaisian (Bateman & Rothwell, 1990; Bateman *et al.*, 2016). Parent plant unknown.

***Ruxtonia minuta*** Galtier, Feist, Talent & Meyer-Berthaud. As described by Galtier *et al*. (2007) comprising uni- and biovulate cupules bearing ovules of the *Hydrasperma*-type (Long, 1961*b*; Rothwell & Wight, 1989), and a single isolated, charcoalified ovule of the *Hydrasperma*-type from the same locality. Specimens from calcareous nodules in the Ruxton Formation, Queensland, Australia, dated as mid-Tourniasian (Galtier *et al*., 2007).

***Stamnostoma huttonese*** Long. Cupulate ovules as described by Long (1960*c*, 1975). Ballagan Formation, Whiteadder river, Berwickshire, UK. Late Tournaisian. Parent plant considered by Long (1979) to comprise stems of *Pitus primaeva*, leaves of ‘*Sphenopteris*’ (*Eusphenopteris*) *affinis*, petioles of *Lyginorachis papilio*, prepollen of *Colatisporites* *decorus* within *Telangium* pollen organs by Long (1979). Retallack & Dilcher (1988) considered the leaves of this conceptual whole-plant more likely to be *Aneimites acadica*.

***Stamnostoma oliveri*** Rothwell & Scott. Cupulate ovules as described by Rothwell & Scott (1992). Oxroad Bay, East Lothian, UK. Ballagan Formation, late Tournaisian. Parent plant unknown.

***Genomosperma kidstonii*** (Calder) Long. As described by Long (1959) and Meade *et al.* (2021) including features of its cupule. Specimens from the Langton Burn, Hutton Mill, Blanerne Bridge (Whiteadder), Chirnside Bridge (Whiteadder), Blackadder Bridge, Pease Bay (Long, 1959) localities in Berwickshire and Oxroad Bay (Bateman & Rothwell, 1990) in East Lothian, UK. Ballagan Formation, Ivorian regional substage, late Tournaisian (Bateman & Rothwell, 1990; Bateman *et al.*, 2016). Parent plant unknown.

***Salpingostoma dasu*** Gordon. As described by Gordon (1942), augmented by studies demonstrating it was borne in a cupule of *Calathospermum fimbriatum* Barnard (Barnard, 1960), and correlated with *in situ* pollen grains by Long (1975, p. 280). Ballagan Formation, Oxroad Bay, UK (Bateman & Rothwell, 1990); Late Tournaisian (Bateman & Rothwell, 1990; Bateman *et al.*, 2016). Parent plant speculated by Retallack & Dilcher (1988) to include leaves of *Sphenopteridium capillare* and stems of *Calathopteris heterophylla*.

***Tantallosperma setigera*** Barnard & Long. As described by Long (1961*b*, 1975), Barnard & Long (1973) and Bateman & Rothwell (1990). Oxroad Bay, East Lothian, UK. Ballagan Formation, late Tournaisian. Parent plant unknown.

***Dolichosperma sexangulatum*** Long. As described by Long (1961*b*, 1975) and Bateman & Rothwell (1990). Langton Burn, Berwickshire, UK (Long, 1961*b*), within the Ballagan Formation, late Tournaisian. Borne in a dichotomous, open cupule (Bateman & Rothwell, 1990) but other organs of parent plant unknown.

***Deltasperma fouldenense*** Long. As described by Long (1961*a*) and Bateman & Rothwell (1990). Specimens from Hutton Bridge and Blue Scaur in Berwickshire, and Oxroad Bay, East Lothian, UK (Long, 1961*a*). Ballagan Formation, late Tournaisian. Parent plant unknown.

***Hirsutisperma rothwellii*** Hilton, Galtier & Scott. Described by Scott *et al.* (2019) from scanning electron microscopy (SEM) images and X-ray micro-computed tomographic data of the specimen. Specimen preserved as charcoal within limestones from the Kingshorn Volcanic Group, Kingswood, UK, dated to the mid-late Asbian regional substage of the Viséan (Scott *et al.*, 2019). Parent plant unknown.

***Lyrasperma scotica*** Long. Described by Long (1960*b*) and supplemented with additional observations of specimens in the Albert Long slide collection at the National Museums of Scotland. Parent plant unknown but suggested associates include leaves of *Sphenopteridium pachyrrachis*, petioles of *Kalymma tuediana* and stems of *Stenomyelon tuedianum* (Retallack & Dilcher, 1988). Hydrasperman seed-fern having affinities within the Lyginopteridales. Specimens from the Langton Glen, Langton Burn, Cumledge, Broomhouse, Marden, Edrom, Horse Roads and Burnmouth localities in Berwickshire, UK (Long, 1960*b*), within the Ballagan Formation, late Tournaisian. Parent plant tentatively reconstructed by Retallack & Dilcher (1988) as comprising leaves of *Sphenopteridium pachyrrachis* and stems of *Stenomyelon tuedianum*, with ovules potentially born on *Alcicornopteris convoluta* “cupules” (however, we consider *A. convoluta* more likely to have borne the pollen organs of this or another whole-plant species rather than ovules).

***Eurystoma angulare*** Long. Ovules as described by Long (1960*b*, 1975) and cupules bearing ovules by Long (1965). Specimens from Hutton Mill, Broomhouse, Chirnside, Gavinton and Langton Glen in Berwickshire, UK (Long, 1960*b*). Specimens from Langton Glen and the Whiteadder river at Edrom House, Hutton Mill and Edington Mill. Ballagan Formation, late Tournaisian. Parent plant unknown.

***Eosperma oxroadense*** Barnard. As described by Barnard (1959) and Bateman & Rothwell (1990). Oxroad Bay, Berwickshire, UK. Ballagan Formation, late Tournaisian. Parent plant unknown.

***Eccroustosperma langtonense*** Long. As described by Long (1961*b*, 1975) but with abortive megaspores and ovules borne in clusters on a short stalk documented by Long (1975, p. 278). Specimens from Langton Glen; Ballagan Formation, late Tournaisian. Parent plant unknown.

***Sphaerostoma ovale*** Benson. As described by Benson (1914). Preserved in chert within the Pettycur Limestone, Kingshorn Volcanic Group, Pettycur, Scotland. Early Visean (Scott *et al.*, 2019). Parent plant unknown.

***Lagenostoma lomaxii*** Oliver & Scott. As described by Oliver & Scott (1904) from European coal balls extracted from the Hauptflöz (Germany), 1^st^ (Cheshire, UK), Union (Lancashire, UK), Upper Foot (Lancashire, UK), Bouxharmont (Belgium), Finefrau (Netherlands and Rhur coalfields) coal seams (Galtier, 1996). Species ranges stratigraphically from the top of the Namurian C to the Westphalian A (Galtier, 1996), spanning the full range of the Bashkirian stage of the Carboniferous. Ovule of the reconstructed plant of the same name also comprising stems of *Lyginorachis oldhamia*, roots of *Kaloxylon hookeri*, petioles of *Rachiopteris aspera*, leaves of *Sphenopteris hoeninghausii*.

***Lagenostoma ovoides*** Williamson. As described by Williamson (1877), augmented by information from Prankerd (1912) and on the megagametophyte by Long (1944). Specimens from European coal-ball assemblages in the Hauptflöz (Germany), 1^st^ (Cheshire, UK), Union (Lancashire, UK), Upper Foot (Lancashire, UK), Halifax (Yorkshire, UK), Bouxharmont (Belgium), Finefrau (Netherlands and Rhur coalfields) coal seams (Galtier, 1996). Species ranges stratigraphically from the top of the Namurian C to the Westphalian A (Galtier, 1996), spanning the full range of the Bashkirian stage of the Carboniferous. Parent plant unknown.

***Physostoma elegans*** (Williamson) Oliver. As described by Williamson (1877) and Oliver (1909), preserved in coal balls from the Dullesgate and Bacup mines (Union coal seam) and Shore mine (Upper Foot coal seam) in Lancashire (UK), the Halifax seam in Yorkshire, and the Finefrau seam in the Netherlands and Germany (Phillips, 1980; Galtier, 1996). These beds were deposited in the Namurian C to Westphalian A (Galtier, 1996), corresponding with the Bashkirian stage of the Carboniferous. Parent plant unknown but likely a hydrasperman-type pteridosperm.

***Tyliospermum orbiculatum*** Mamay. As described by Mamay (1954) from coal balls from the West Mineral Mine in Kansas, North America. Coal balls occur in the Mineral-Fleming (mid-Desmoinesian) and Iron Post (mid-Desmoinesian) coals (Mamay, 1954; Phillips, 1980), equivalent to the late Moscovian. Parent plant unknown.

***Conostoma augustodunensis*** (Renault) Galtier. Specimens preserved in chert from the Grand Croix locality in the St. Étienne Basin of the Massif Central, France (Doubinger *et al.*, 1995; Galtier, 2013) within the Rie de Dier Formation or in sediments immediately overlying it. According to Doubinger *et al.* (1995), the chert was either deposited during the Stephanian A or taphonomically re-worked during the Stephanian B (Doubinger *et al.*, 1995; Galtier, 2008, 2013), equating to the mid-late Kasimovian to earliest Gzhelian stages of the late Pennsylvanian (Galtier, 2008). Ovules were born in the multi-ovulate cupule *Gnetopsis elliptica* (Renault & Zeiller) Galtier (Galtier, 2013).

***Conostoma oblongum*** Williamson. As described by Oliver & Salisbury (1911). Specimens from coal-ball assemblages in the 1^st^, Union, Upper Foot and Halifax (UK), Bouxharmont (Belgium), and Finefrau (Germany) coal seams (Galtier, 1996). The species is confined to the Westphalian A (Galtier, 1996), equivalent to the latest part of the Bashkirian stage of the late Pennsylvanian. North American specimens in coal balls from the Copland (mid-Atokan), Rock Island (earliest Desmoinesian) and Springfield (mid-Desmoinesian) coal seams (Phillips, 1980), correlating with the middle half of the Moscovian stage of the Carboniferous. Hydrasperman pteridosperm but identity of parent plant unknown; presumed to have stems of *Heterangium*.

***Conostoma villosum*** Rothwell & Eggert (Rothwell & Eggert, 1970). Species described from coal balls in the Calhoun Coal, Mattoon Formation at Berryville, Illinois (Rothwell & Eggert, 1970). The Calhoun coal was deposited at the end of the Missourian regional substage of North America (Phillips, 1980; Willard *et al*., 2007), equivalent to the latest Kasimovian and earliest Gzhelian stages of the Carboniferous.

***Mitrospermum compressum*** (Williamson) Arber (1910*b*). Data from Williamson (1877), Arber (1910*a*,*b*), Taylor & Stewart (1964) and Long (1977*a*). Specimens from European coal balls from the 1st, Union and Upper Foot (UK) and Finefrau (Germany) coal seams of Westphalian A age (Galtier, 1996), equivalent to the latest part of the Bashkirian stage of the Carboniferous. North American specimens from coal balls in the Copland coal seam (mid-Atokan), equivalent to the early Moscovian stage of the Carboniferous. Ovule of the *Gothania*-type cordaitean coniferophyte *Mesoxylon sutcliffii* (Trivett & Rothwell, 1991).

***Callospermarion pusillum*** Eggert & Delevoryas. Data from Eggert & Delevoryas (1960), Stidd & Hall (1970) and Rothwell (1971*b*, 1980). Specimens occur in coal balls from the late Pennsylvanian of North America from the Ames (mid-Missourian), Calhoun (late Missourian), and Duquesne (late Missourian) coal seams (Rothwell, 1975, 1980; Phillips, 1980), correlating with the mid-late Kasimovian stage of the Carboniferous. Ovule of the callistophytalean seed fern *Callistophyton poroxyloides* (Rothwell, 1980).

***Callospermarion undulatum*** (Neely) Rothwell. Data from Neely (1951), Rothwell (1980) and Hilton *et al.* (2002). Specimens occur in coal balls from the Mineral-Fleming (mid-Desmoinesian), Bevier (mid-Desmoinesian), Summum (mid-Desmoinesian), Springfield (mid-late Desmoinesian), Herrin (mid-late Desmoinesian) and Baker (late Demoinesian) coal seams (Rothwell, 1980, 1981; Phillips, 1980), correlating with the late Moscovian stage of the Carboniferous, in cherts from the latest Pennsylvanian (Gzhelian) to earliest Permian (Asselian) of France (Rothwell, 1980, 1981), and in coal balls from the earliest Permian (Asselian-Sakmarian) of North China (Hilton *et al.*, 2002). Ovule of the callistophytalean pteridosperm *Callistophyton boysettii* (Rothwell, 1981). Parent plant of the Chinese specimens unknown but probably similar to that of the European and American ovule-species.

***Cardiocarpus dabiziae*** Hilton, Rothwell, Li, Liang & Galtier. Data from Hilton *et al.* (2001). Specimens preserved in reworked volcanic ash from the early Permian (Asselian–Sakmarian) aged Taiyuan Formation of North China (Hilton *et al.*, 2001). Parent plant unknown, most likely cordaitalean coniferophyte as ovules occur terminally on an extended sporophyll (Hilton *et al.*, 2001).

***Cardiocarpus samaratus*** Wang & Tian. As described by Wang and colleagues (Wang & Tian, 1991; Wang *et al.*, 2003*b*). Ovule of the *Cathayanthus*-type cordaitean coniferophyte *Shanxioxylon sinense* (Wang *et al.*, 2003*b*; Hilton *et al.*, 2009*b*). From coal balls in the Asselian (Cisuralian, early Permian) Taiyuan Formation of North China.

***Cardiocarpus taiyuanensis*** Hilton, Wang & Tian. Data from Hilton *et al.* (2003) and Hilton *et al.* (2009*b*). Specimens preserved in coal balls from the Asselian (Cisuralian, early Permian) of North China (Hilton *et al.*, 2003). Ovule of the *Cathayanthus*-type cordaitean coniferophyte *Shanxioxylon taiyuanense* (Wang, 1997; Hilton *et al.*, 2009*b*).

***Cardiocarpus tuberculatus*** Wang, Hilton & Tian. Based on description from Wang *et al.* (2003*a*) and Hilton *et al.* (2009*a*). Specimens preserved in coal balls from the Asselian (Cisuralian, early Permian) of North China. Cardiocarpalean-type ovule produced by the cordaitean whole-plant *Cordaixyon tianii* (Hilton *et al.,* 2009*a*,*b*).

***Muricosperma guizhouensis*** Seyfullah & Hilton. Data from Seyfullah *et al.* (2010) and personal observations by J. Hilton. Preserved in reworked volcanic ash from the Xuanwei Formation of South China. Wuchiapingian, Lopingian (late Permian). Parent plant unknown.

***Cardiocarpus huopuensis*** Wang *et al.* (Wang *et al.*, 2006). Occurs in volcaniclastic tuffs from the Xuanwei Formation of southern China, deposited during the Wujiapingian stage of the Lopingian (late Permian) (Neregato *et al.*, 2016). Parent plant unknown.

***Stephanospermum trunctatum*** (Wang *et al.*) Spencer *et al.* Data from Spencer *et al.* (2013*b*). Specimens preserved in coal balls from the Changshingian stage of the Lopingian (late Permian) Wangjiazhai Formation of South China. Parent plant unknown.

***Chaonostoma verruculosum*** Klavins *et al.* (Klavins *et al.*, 2001). Data from Klavins *et al.* (2001). Specimens from the Skaar Ridge, Transantarctic Mountains, Antarctica, preserved in chert within the Buckley Formation. Dated to the Lopingian (late Permian; Klavins *et al.*, 2001). Parent plant unknown, presumed to be a glossopterid.

***Lakkosia kerasata*** Ryberg (Ryberg, 2010). Data from Ryberg (2010). Specimens from the Skaar Ridge, Transantarctic Mountains, Antarctica, preserved in chert within the Buckley Formation. Dated to the Lopingian (late Permian; Klavins *et al.*, 2001). Born in clusters on the adaxial surface of a megasporophyll belonging to a glossopterid plant.

***Lonchiphyllum aplospermum*** Ryberg & E. L. Taylor (Ryberg & Taylor, 2013). Data from Ryberg & Taylor (2013). Specimens from the Skaar Ridge, Transantarctic Mountains, Antarctica, preserved in chert within the Buckley Formation. Dated to the Lopingian (late Permian; Ryberg & Taylor, 2013). Parent plant unknown, presumed to be a glossopterid.

***Homevaleia gouldii*** Nishida *et al.* (Nishida *et al.*, 2007). Description based on specimens preserved in chert from the Homevale locality in Queensland, Australia, documented by Nishida *et al.* (2003, 2004, 2007). Specimens occur in the Blackwater Group of Bowen Basin, dated as Lopingian (late Permian) (Nishida *et al.*, 2007). Ovules attached to a megasporophyll produced by a glossopterid plant.

***Illawarraspermum ovatum*** McLoughlin & Mays (McLoughlin *et al*., 2019). Specimens preserved in chert from the lower Wilton Formation, Illawarra Coal Measures Group in the Sydney Basin, Australia, dated to the Wuchiapingian stages of the Lopingian (late Permian) (McLoughlin *et al.*, 2019). Parent plant unknown, presumed to be a glossopterid.

***Pachytestopsis tayloriorum*** McLoughlin, Bomfleur & Drinnan (McLoughlin *et al.*, 2018). Specimens preserved in silicified peat from the Fort Cooper Coal Measures in the Blackwater Group of the Bowen Basin, Australia, dated to the Lopingian (late Permian). Parent plant unknown but presumed to be a glossopterid based on pollen observed *in situ* in the micropyle.

***Hexapterospermum delevoryii*** Taylor. As described by Taylor (1966) and Matten & Hopkins (1967) based on specimens preserved in coal balls from the Herrin No. 6 coal seam (late Desmoinesian) of North America (Phillips, 1980), dated to the late Moscovian stage of the Carboniferous. Parent plant unknown.

***Rhynchosperma quinnii*** (Taylor & Eggert) Dunn, Rothwell & Mapes (Dunn *et al.*, 2002). As described by Taylor & Eggert (1967) and Dunn *et al.* (2002), based on pyritized fossils from marine black shales from the Fayetteville Formation in Arkansas, USA. Fossils are dated to the Chesterian North American regional substage and the Pendellian, equivalent to the late Visean and early Serpukhovian stages in the late Mississippian (Dunn *et al.*, 2002).

***Sergeia neuburgii*** Rothwell, Mapes & Mapes. Specimens are preserved as limonite after pyrite and also partially preserved in phosphate and carbonate within marine black shales from the Finis Shale Member of the Graham Formation in Texas, USA (Rothwell *et al.*, 1996). The Finis Shale was dated to the Virgilian regional North American substage (Rothwell *et al.*, 1996) that correlates with the early to middle Gzhelian (Late Pennsylvanian). Parent plant unknown, but suspected to correspond with the ovulate cone of a putative vojnovskyalean coniferophyte (Rothwell *et al.*, 1996).

***Emporia lockardii*** Mapes & Rothwell. Data from Mapes & Rothwell (1984, 1991). Specimens are preserved in clasts in a channel conglomerate and overlying limestones as compression/impressions and partial permineralizations in the Hartford Limestone of the Topeka Limestone Formation in Hamilton Quarry, Kansas, USA (Mapes & Rothwell, 1984). The Hartford Limestone was dated to the Virgilian regional North American substage (Mapes & Rothwell, 1984) that correlates with the early to middle Ghzelian (Late Pennsylvanian). Ovule of the Palaeozoic conifer of the same name (Mapes & Rothwell, 1991).

***Emporia cryptica*** Hernandez-Castillo *et al*. Data from Hernandez-Castillo *et al.* (2009); locality, age and preservational context as for *E. lockardii* (above).

***Codonospermum anomalum*** Brongniart. Data from Brongniart (1881) and Combourieu & Galtier (1985). Specimens preserved in chert from Grand Croix, France. For stratigraphic information see above entry for *Conostoma augustodunensis*.

***Codonospermum olivaeforme*** (Williamson) Galtier. Data from Williamson (1877), Scott & Maslen (1906) and Combourieu & Galtier (1985). Frequent in British coal balls of early Moscovian age.

***Polypterospermum renaultii*** Brongniart. Data from Brongniart (1881) and Combourieu & Galtier (1985). Specimens preserved in chert from Grand Croix, France. For stratigraphic information see above entry for *Conostoma augustodunensis*.

***Polylophospermum stephanense*** Brongniart. Data from Brongniart (1881) and Combourieu & Galtier (1985). Specimens preserved in chert from Grand Croix, France. For stratigraphic information see above entry for *Conostoma augustodunensis*.

***Colpospermum multinerve*** Renault emend. Combourieu & Galtier. Data from Brongniart (1881) and Combourieu & Galtier (1985). For stratigraphic information see above entry for *Conostoma augustodunensis*.

***Cardiocarpus drupaceus*** Brongniart. Data from illustrations and descriptions in Brongniart (1874, 1881), Grand’Eury (1877), Bertrand (1908*c*) and Seward (1917), supplemented by recent observations of Doubinger *et al.* (1995). Specimens from cherts at Grand Croix, France. For stratigraphic information see above entry for *Conostoma augustodunensis*. Parent plant unknown, possibly a *Cordaitanthus*-type cordaitalean coniferophyte.

***Leptocaryum avellanum*** Brongniart. Data from Brongniart (1874, 1881), Bertrand (1908*b*) and Doubinger *et al.* (1995). Specimens from cherts at Grand Croix, France. For stratigraphic information see above entry for *Conostoma augustodunensis*.

***Cycadinocarpus augustodunensis*** (Brongniart) Bertrand. Data from Brongniart (1874, 1881) and Bertrand (1908*b*). Specimens in cherts from Autun, France, dated to the earliest Permian Asselian stage (Doubinger *et al*., 1995; Galtier, 2008) and Ogliastra, Sardinia, dated to the early Permian Asselian–Sakhmarian boundary (Galtier *et al*., 2011). Generic affinity of ovules unknown at present, and validity of the genus *Cycadinocarpus* uncertain; possibly synonymous with the over-inflated genus *Cardiocarpus* (Galtier, 2008). Parent plant unknown, but most likely a cordaitalean coniferophyte.

***Diplotesta avellana*** (Brongniart) Bertrand. Data from Brongniart (1874, 1881), Bertrand (1907*a*) and Doubinger *et al.* (1995). Specimens preserved in chert from Grand Croix, France. For stratigraphic information see above entry for *Conostoma augustodunensis*. Parent plant most likely a cordaitalean coniferophyte (Galtier, 2008).

***Diplotesta grandeuryana*** (Brongniart) Bertrand. Data from Brongniart (1874, 1881), Bertrand (1907*a*) and Doubinger *et al.* (1995). Specimens preserved in chert from Grand Croix, France. For stratigraphic information see above entry for *Conostoma augustodunensis*. Parent plant most likely a cordaitalean coniferophyte (Galtier, 2008).

***Taxospermum gruneri*** Brongniart. Data from illustrations and descriptions by Brongniart (1874, 1881), Bertrand (1907*c*), Seward (1917) and Doubinger *et al.* (1995). Specimens preserved in chert from Grand Croix, France. For stratigraphic information see above entry for *Conostoma augustodunensis*.

***Plectilospermum elliotii*** Taylor & Taylor. As described by Taylor & Taylor (1987). Specimens preserved in chert from Skaar Ridge in the Transantarctic Mountains of Antarctica. Dated late Permian (Taylor & Taylor, 1987), equating to the Lopingian.

***Conostoma chappellicum*** Stubblefield & Rothwell. As described by Stubblefield & Rothwell (1980), including specimens previously assigned to *Conostoma oblongum* by Rothwell (1971*b*). Specimens preserved in coal balls from the Copland (mid-Atokan) coal seam in eastern Kentucky (Rothwell, 1971*b*; Phillips, 1980), correlating with the early Moscovian stage. Hydrasperman pteridosperm but specific parent plant unknown.

***Cardiocarpus magnicellularis*** Baxter & Roth. Data from Baxter & Roth (1954). Occurs in coal balls from the Pennsylvanian of North America in the Rock Island (earliest Desmoinesian) and Buffaloville (earliest Desmoinesian) coal seams (Baxter & Roth, 1954; Phillips, 1980), corresponding with the mid-Moscovian. Whole-plant species unknown, presumed to be a cordaitalean coniferophyte (Baxter & Roth, 1954).

***Cardiocarpus oviformis*** Leisman. Data from Leisman (1961) and Rothwell (1982*a*, 1993). Specimens occur in Pennsylvanian coal balls from North America in the Buffaloville (early Desmoinesian), Murphysboro (early Desmoinesian), Mineral-Fleming (early Desmoinesian), Colchester (mid-Desmoinesian), Bevier (mid-Desmoinesian), Summum (mid-Desnoinesian), Springfield (mid-Desmoinesian), Herrin (late Desmoinesian), Baker (late Desmoinesian), Friendsville (mid-Missourian) and Calhoun (latest Missourian) coal seams (Phillips, 1980), correlating with the late Moscovian to early Kasimovian stages. Ovules of the *Cordaitanthus*-type cordaitalean coniferophyte *Cordaixylon dumusum* (Rothwell, 1982*a*, 1993; Rothwell & Warner, 1984).

***Cardiocarpus sclerotesta*** Brongniart (1881). Data from Brongniart (1881), Bertrand (1908*a*), Seward (1917), and Doubinger *et al.* (1995). Specimens preserved in chert from the Grand Croix locality in the St. Étienne Basin of the Massif Central, France (Doubinger *et al*., 1995).

***Cardiocarpus spinatus*** Graham (1935). Data from Andrews & Felix (1952) and Roth (1955). Specimens preserved in coal from the Rock Island (earliest Desmoinesian), Secor (earliest Desmoinesian), Buffaloville (early Desmoinesian), Murphysboro (early Desmoinesian), Weir-Pittsburg (early Desmoinesian), Mineral-Fleming (mid-Desmoinesian) and Bevier (mid-Desmoinesian) coal seams (Phillips, 1980), correlating with the mid-Moscovian. Ovule of the whole-plant cordaitalean coniferophytes *Mesoxylon birame* (Trivett & Rothwell, 1985) and *Cordaixylon iowensis* (Trivett & Rothwell, 1991; Trivett, 1992).

***Cardiocarpus tritolopus*** Serlin (1982). Data from Serlin (1982). Specimens preserved in coal balls from the Path Fork Coal, Hance Formation, USA, dating from the Moscovian (Westphalian B; Hubbard *et al*., 2002).

***Cyclospermum tenue*** (Brongniart) Seward (1917). Data from Brongniart (1881), Bertrand (1908*a*), and Seward (1917). Specimens preserved in chert from the Grand Croix locality in the St. Étienne Basin of the Massif Central, France (Brongniart, 1881). For stratigraphic information see above entry for *Conostoma augustodunensis*.

***Mitrospermum vinculum*** Grove & Rothwell. Data from Grove & Rothwell (1980). From coal balls in the Duquesne coal seam (late Missourian) correlating with the late Kasimovian stage of the Carboniferous. Ovule of the *Gothania*-type cordaitalean coniferophyte *Mesoxylon priapi* (Scott & Maslen, 1910; Trivett & Rothwell, 1985).

***Mitrospermum florinii*** (Darrah) Baxter (Baxter, 1972). Data from Darrah (1953, 1967) and Baxter (1972). Specimens from coal balls in the Rock Island (earliest Desmoinesian) seam (Phillips, 1980), correlating with the mid-Moscovian. Parent plant unknown, most likely a *Gothania*-type cordaitalean coniferophyte.

***Mitrospermum leeanum*** (Kern & Andrews) Baxter. Data from Kern & Andrews (1946) and Baxter (1972). From Pennsylvanian coal balls in North America from the Rock Island (earliest Desmoinesian) and Buffaloville (earliest Desmoinesian) coal seams (Phillips, 1980), correlating with the mid-Moscovian. Parent plant and affinities unknown, most likely a *Gothania*-type cordaitalean coniferophyte.

***Nucellangium glabrum*** (Darrah) Andrews. Data from Darrah (1941), Andrews (1949), Segal (1969) and Stidd & Cosentino (1976). Specimens from coal balls in Rock Island (earliest Desmoinesian) and Buffaloville (earliest Desmoinesian) coal seams (Phillips, 1980), correlating with the mid-Moscovian. Parent plant unknown but suspected of being the cordaitalean coniferophyte that produced *Cordaitanthus zeilleri*-type fertile shoots (Stidd & Cosentino, 1976).

***Rhabdospermum conicum*** (Grand’Eury) Doubinger *et al.* Data from Brongniart (1881), Bertrand (1907*b*) and Doubinger *et al*. (1995) and including J. Hilton’s examination of images of specimens in the Museum Nationale Naturale d’Histoire (Paris). Taxonomic treatment follows Seward (1917) who transferred the species from the compression/impression genus *Rhabdocarpus*. Specimens preserved in chert from the Grand Croix locality in France. For stratigraphic information see above entry for *Conostoma augustodunensis*. Parent plant unknown, suspected of being a cordaite (Stidd & Cosentino, 1976).

***Rhabdospermum subtunicatum*** (Grand’Eury) Doubinger *et al.* Data from Brongniart (1881), Bertrand (1907*b*), Doubinger *et al.* (1995) including J. Hilton’s examination of images of specimens in the Museum Nationale Naturale d’Histoire (Paris). Account follows Seward (1917) who transferred the species from the compression/impression genus *Rhabdocarpus* and updated the specific epithet to match the new genus name. Specimens preserved in chert from the Grand Croix locality in France. For stratigraphic information see above entry for *Conostoma augustodunensis*. Parent plant unknown, suspected of being a cordaite (Stidd & Cosentino, 1976).

***Pachytesta crenulata*** Raymond & McCarty. Specimens described by Raymond & McCarty (2009). Coal balls from the Cliffland and Blackoak coals (latest Atokan: Raymond & McCarty, 2009), equivalent to the early Moscovian stage of the Carboniferous. Medullosan-type pteridosperm, parent plant unknown.

***Pachytesta illinoensis*** (Arnold & Steidmann) Stewart. Data from Stewart (1954). From coal balls in the Bevier (mid-Desmoinesian), Springfield (mid-Desmoinesian), Herrin (mid-Desmoinesian), Parker (mid-Missourian) and Calhoun (latest Missourian) coal seams (Phillips, 1980) in North America, correlating with the late Moscovian to late Kasimovian stages of the Carboniferous. Medullosan-type pteridosperm; parent plant unknown.

***Pachytesta saharasperma*** Taylor. Described by Taylor (1965) from coal balls within the Springfield (mid-Desmoinesian) and Herrin (mid-Desmoinesian) coal seams (Phillips, 1980), correlating with the late Moscovian stage of the Carboniferous. Medullosan-type pteridosperm, parent plant unknown.

***Pachytesta stewartii*** Taylor & Delevoryas. As described by Taylor & Delevoryas (1964). Preserved in coal balls from the Buffaloville (early Desmoinesian), Weir-Pittsburg (early Desmoinesian), and Herrin (late Desmoinesian) coal seams in North America, correlating with the late Moscovian. Medullosan-type pteridosperm, parent plant unknown.

***Stephanospermum elongatum*** Hall. Data from Hall (1954) and Leisman & Roth (1963). Preserved in coal balls from the Herrin (late Desmoinesian), Calhoun (latest Missourian), Opdyke (late Missourian), Bevier (mid-Desmoinesian) and Mineral-Fleming (early Desmoinesian) coals (Phillips, 1980), correlating with the mid-Moscovian to latest Kasimovian stages of the Carboniferous. Medullosan-type pteridosperm, parent plant unknown.

***Stephanospermum akenioides*** Brongniart. As described by Brongniart (1874) and Oliver (1904). Specimens preserved in chert from Grand Croix, France. For stratigraphic information see above entry for *Conostoma augustodunensis*. Parent plant unknown, but likely a medullosan-type pteridosperm.

***Stephanospermum konopeonus*** (Langford) Drinnan, Schramke & Crane. Data from Langford (1958) and Drinnan *et al.* (1990). Preserved in siderite nodules from the Calhoun (latest Missourian) coal in Illinois, correlating with the late part of the Kasimovian stage of the Carboniferous. Medullosan-type pteridosperm, parent plant unknown.

***Stephanospermum tridentatum*** Serbet & Rothwell**.** Described by Serbet & Rothwell (1995). Preserved in coal balls from the Duquesne Coal at Steubenville, eastern Ohio. Dated to the Stephanian B (Serbet & Rothwell, 1995), equivalent to the later part of the Kasimovian stage. Medullosan-type pteridosperm, parent plant unknown.

***Albertlongia incostata*** Taylor. As described by Taylor (1967). Specimens from coal balls in the Herrin No. 6 coal seam of North America, dated to the late Desmoinesian (Phillips, 1980), correlating with the latest Moscovian. Parent plant unknown.

**Excluded taxa**

The following taxa have been removed from the associated data matrix (Data S1):

***Conostoma anglo-germanicum*** Oliver & Salisbury. As described by Oliver & Salisbury (1911). Specimens preserved in coal balls from the Union (UK), Upper Foot (UK), Bouxhardmont (Belgium) and Finefrau (Netherlands and Germany) coal seams (Galtier, 1996) that formed during the Westphalian A, equivalent to the latest Bashkirian stage of the Pennsylvanian. Specimens from North America in coal balls from the Copland coal (mid-Atokan) (Phillips, 1980), equivalent to the early Moscovian stage of the Carboniferous. Parent plant unknown, presumed to be a hydrasperman pteridosperm with stems of *Heterangium*. Excluded as the analysis includes three other species of *Conostoma* and lacks sufficient characters for species-level distinctions.

***Coumiasperma remyi*** Galtier & Rowe. Single specimen preserved in marine deposits of mid-Tournaisian (early Carboniferous) age from the Montagne Noire, France. Excluded because of its notoriously enigmatic affinity – either a non-hydrasperman seed with a consistently solid nucellar apex (Galtier & Rowe, 1989, 1991), an ontogenetically immature hydrasperman pteridosperm in which the pollen chamber has not yet differentiated (e.g. Rothwell, 1986), or a teratological ovule analogous with an indisputably teratological *Pullaritheca* cupule described by Long (1977*b*) and Bateman & DiMichele (2002).

REFERENCES

Andrews, H. N. (1949). *Nucellangium*, a new genus of fossil seeds previously assigned to *Lepidocarpon*. *Annals of the Missouri Botanical Garden* **36**, 479–505.

Andrews, H. N. & Felix, C. J. (1952). The gametophyte of *Cardiocarpus spinatus* Graham. *Annals of the Missouri Botanical Garden* **39**, 127–135.

Arber, A. (1910*a*). A note on *Cardiocarpon compressum* Will. *Proceedings of the Cambridge Philosophical Society* **15**, 393.

Arber, A. (1910*b*). On the structure of the Palaeozoic seed *Mitrospermum compressum* (Will.). *Annals of Botany* **24**, 37–39.

Barnard, P. D. W. (1959). On *Eosperma oxroadense* gen. et sp. nov.: a new Lower Carboniferous seed from East Lothian. *Annals of Botany* **23**, 285–296.

Barnard, P. D. W. (1960). *Calathospermum fimbriatum* sp. nov., a Lower Carboniferous pteridosperm cupule from Scotland. *Palaeontology* **3**, 265–275.

Barnard, P. D. W. & Long, A. G. (1973). On the structure of a petrified stem and some associated seeds from the Lower Carboniferous rocks of East Lothian, Scotland. *Transactions of the Royal Society, Edinburgh* **69**, 91–108.

Bateman, R. M. & DiMichele, W. A. (2002). Generating and filtering major phenotypic novelties: neoGoldschmidtian saltation revisited. In *Developmental Genetics and Plant Evolution* (eds Q. C. B. Cronk, R. M. Bateman and J. A. Hawkins), pp. 109–159. Taylor & Francis, London.

Bateman, R. M. & Rothwell, G. W. (1990). A reappraisal of the Dinantian floras at Oxroad Bay, East Lothian, Scotland. 1. Floristics and the development of whole-plant concepts. *Transactions of the Royal Society of Edinburgh B* **81**, 127–159.

Bateman, R. M., Stevens, L. G. & Hilton, J. (2016). Stratigraphy and palaeoenvironments of the Loch Humphrey Burn lagerstätte and other Mississippian plant-bearing localities of the Kilpatrick Hills, southwest Scotland. *PeerJ* **4,** 1700.

Baxter, R. W. (1972). A comparison of the Paleozoic seed genera, *Mitrospermum* and *Kamaraspermum*. *Phytomorphology* **21**, 108–121.

Baxter, R. W. & Roth, E. A., (1954). *Cardiocarpus magnicellularis* sp. nov., a preliminary report. *Transactions of the Kansas Academy of Science* **57**, 458–460.

Benson, M. J. (1914). *Sphaerostoma ovale* (*Conostoma ovale* et *intermedium*, Williamson), a Lower Carboniferous ovule from Pettycur, Fifeshire, Scotland. *Transactions of the Royal Society, Edinburgh* **50**, 1–15.

Bertrand, M. C. E. (1907*a*). Les charactéristiques du genre *Diplotesta* de Brongniart. *Bulletin de la Société Botanique du France* **54**, 398–402.

Bertrand, M. C. E. (1907*b*). Les charactéristiques du genre *Rhabdocarpus* d’apres les préparations de la collection B. Renault. *Bulletin de la Société Botanique du France* **54**, 654–664.

Bertrand, M. C. E. (1907*c*). Les charactéristiques du genre *Taxospermum* de Brongniart. *Bulletin de la Société Botanique du France* **54**, 213–224.

Bertrand, M. C. E. (1908*a*). La spécification des *Cardiocarpus* de la collection Renault. *Bulletin de la Société Botanique du France* **55**, 454–462.

Bertrand, M. C. E. (1908*b*). Les caractéristiques du *Cycadinocarpus augustodunensis* de B. Renault. *Bulletin de la Société Botanique du France* **55**, 326–333.

Bertrand, M. C. E. (1908*c*). Les charactéristiques du genre *Cardiocarpus* d’apres les graines silicifiée étudiées par Ad. Brongniart et B. Renault. *Bulletin de la Société Botanique Francais* **55**, 391–396.

Brongniart, A. (1874). Études sur les graines fossiles trouvées à l’état silicifié dans le terrain houiller de Saint-Étienne. *Extraits des Annales des Sciences Naturelle, Botanique, 5^th^ Séries* **20**, 234–265.

Brongniart, A. (1881). *Researches sur les Graines Fossiles Silicifieés*. Masson, Paris.

Combourieu, N. & Galtier, J. (1985). Nouvelles observations sur *Polypterospermum*, *Polylophospermum*, *Colpospermum* et *Codonospermum*, ovules de Ptéridospermales du Carbonifère supérieur Français. *Palaeontographica B* **196**, 1–29.

Darrah, W. C. (1941). The fossil flora of Iowa coal balls IV. *Lepidocarpon*. *Harvard University Botanical Museum Leaflet* **9**, 85–100.

Darrah, W. C. (1953). A new cardiocarp from the Pennsylvanian of Iowa. *Paleobotanical Notes* **3**, 1–16.

Darrah, W. C. (1967). The structure of *Cardiocarpus florinii* (Darrah), a Pennsylvanian cordaite seed from Iowa. *Proceedings of the Pennsylvania Academy of Science* **40**, 80–86.

Doubinger, J., Vetter, P., Langiaux, J., Galtier, J. & Broutin, J. (1995). *La Flore Fossile du Bassin Houiller de Saint-Étienne (Memoires du Museum National d’Histoire Naturelle).* Museum National d’Histoire Naturelle, Paris.

Drinnan, A. N., Schramke, J. M. & Crane, P. R. (1990). *Stephanospermum konopeonus* (Langford) comb. nov.: A medullosan ovule from the Middle Pennsylvanian Mazon Creek Flora of Northeastern Illinois, U.S.A. *Botanical Gazette* **151**, 385–401.

Dunn, M. T., Rothwell, G. W. & Mapes, G. (2002). Additional observations on *Rhynchosperma quinnii* (Medullosaceae): a permineralized ovule from the Chesterian (Upper Mississippian) Fayetteville Formation of Arkansas. *American Journal of Botany* **89**, 1799–1808.

Eggert, D. A. & Delevoryas, T. (1960). *Callospermarion* - a new seed genus from the Upper Pennsylvanian of Illinois. *Phytomorphology* **10**, 131–138.

Galtier, J. (1996). Coal-ball floras of the Namurian–Westphalian of Europe. *Review of Palaeobotany and Palynology* **95**, 51–72.

Galtier, J. (2008). A new look at the permineralized flora of Grand Croix (Late Pennsylvanian, Saint-Etienne basin, France). *Review of Palaeobotany and Palynology* **152**, 129–140.

Galtier, J. (2013). Reinvestigation of the Carboniferous multiovulate cupule *Gnetopsis eliptica* and its evolutionary significance. *International Journal of Plant Sciences* **174**, 382–395.

Galtier, J., Feist, R., Talent, J. A. & Meyer-Berthaud, B. (2007). New permineralized flora and trilobites from the mid Tournaisian (early Carboniferous) Ruxton Formation, Clarke River Basin, North-East Australia. *Palaeontology* **50**, 223–243.

Galtier, J., Ronchi, A. & Broutin, J. (2011). Early Permian silicified floras from the Perdasdefogu Basin (SE Sardinia): comparison and bio-chronostratigraphic correlation with the floras of the Autun Basin (Massif Central, France). *Geodiversitas* **33**, 43–69.

Galtier, J. & Rowe, N. P. (1989). A primitive seed-like structure and its implications for early gymnosperm evolution. *Nature* **340**, 225–227.

Galtier, J. & Rowe, N. P. (1991). A new permineralized seed-like structure from the basalmost Carboniferous of France. *Neues Jahrbruch Für Geologie und Paläontolgie Abhbeteilung* **183**, 103–120.

Gordon, W. T. (1942). On *Salpingostoma dasu*: a new Carboniferous seed from East Lothian. *Transactions of the Royal Society, Edinburgh* **50**, 427–464.

Graham, R. (1935). Pennsylvanian flora of Illinois as revealed in coal balls. II. *Botanical Gazette* **97**, 156–168.

Grand’Eury, M. (1877). *Flore Carbonifère du Départment de Loire et du Centre de la France.* Mémoires Présentés par Divers Savants à l’Académie des Sciences, Paris.

Grove, G. G. & Rothwell, G. W. (1980). *Mitrospermum vinculum* sp. nov., a cardiocarpalean ovule from the Upper Pennsylvanian of Ohio. *American Journal of Botany* **67**, 1051–1058.

Hall, J. W. (1954). The genus *Stephanospermum* in American coal balls. *Botanical Gazette* **115**, 346–360.

Hernandez-Castillo, G. R., Stockey, R. A., Rothwell, G. W. & Mapes, G. (2009). Reconstruction of the Pennsylvanian-age walchian conifer *Emporia cryptica* sp. nov. (Emporiaceae: Voltziales). *Review of Palaeobotany and Palynology* **157**, 218–237.

Hilton, J. & Bateman, R. M. (2006). Pteridosperms are the backbone of seed-plant evolution. *Journal of the Torrey Botanical Society* **133**, 119–168.

Hilton, J., Rothwell, G. W., Li, C.-S., Wang, S.-J. & Galtier, J. (2001). Permineralized cardiocarpalean ovules in wetland vegetation from Early Permian volcaniclastic sediments of China. *Palaeontology* **44**, 811–825.

Hilton, J., Wang, S. J., Galtier, J. & Bateman, R. M. (2009*a*). Cordaitalean seed plants from the Early Permian Taiyuan Formation of North China. II. Delimitation and reconstruction of the whole–plant *Cordaixylon* *tianii*. *International Journal of Plant Sciences* **170**, 400–418.

Hilton, J., Wang S. J., Galtier, J. & Bateman, R. M. (2009*b*). Cordaitalean seed plants from the Early Permian of North China. III. Reconstruction of the *Shanxioxylon taiyuanense* plant. *International Journal Plant Sciences* **170**, 951–967.

Hilton, J., Wang, S.-J. & Tian, B. (2003). Reinvestigation of *Cardiocarpus minor* (Wang) Li nomen nudum from the Lower Permian of China and its implications for seed plant taxonomy, systematics and phylogeny. *Botanical Journal of the Linnean Society* **141**, 151–175.

Hilton, J., Wang, S.-J., Zhu, W.-Q., Tian, B., Galtier, J. & Wei, A.-H. (2002). *Callospermarion* ovules from the Early Permian of northern China: palaeoforistic and palaeogeographic significance of callistophytalean seed-ferns in the Cathaysian flora. *Review of Palaeobotany and Palynology* **120**, 301–314.

Hubbard, T. E., Miller, T. R., Hower, J. C., Ferm, J. C. & Helfrich, C. T. (2002). The Upper Hance coal bed in southeastern Kentucky: palynologic, geochemical, and petrographic evidence for environmental succession. *International Journal of Coal Geology* **49**, 177–194.

Kern, E. M. & Andrews, H. N. (1946). Contributions to our knowledge of American Carboniferous floras. IX. Some petrified seeds from Iowa. *Annals of the Missouri Botanical Garden* **33**, 291–308.

Klavins, S. D. & Matten, L. C. (1996). Reconstruction of the frond of *Laceya hibernica*, a lyginopterid pteridosperm from the uppermost Devonian of Ireland. *Review of Palaeobotany and Palynology* **93**, 253–268.

Klavins, S. D., Taylor, E. L., Krings, M. & Taylor, T. N. (2001). An unusual, structurally preserved ovule from the Permian of Antarctica. *Review of Palaeobotany and Palynology* **115**, 107–117.

Langford, G. (1958). *The Wilmington Coal Flora from a Pennsylvanian Deposit in Will County, Illinois*. Esconi Associates, Downers Grove, Ill.

Leisman, G. A. (1961). A new species of *Cardiocarpus* in Kansas coal balls. *Transactions of. Kansas Academy of Science* **64**, 117–122.

Leisman, G. A. & Roth, J. (1963). A reconsideration of *Stephanospermum*. *Botanical Gazette* **124**, 231–240.

Long, A. G. (1944). On the prothallus of *Lagenostoma ovoides* Will. *Annals of Botany* **8**, 105–106.

Long, A. G. (1959). On the structure of *Calymmatotheca kidstoni* Calder and *Genomosperma latens* gen. et sp. nov. from the Calciferous sandstone series of Berwickshire. *Transactions of the Royal Society, Edinburgh* **59**, 29–44.

Long, A. G. (1960*b*). On the structure of *Samaropsis scotica* Calder (emended) and *Eurystoma angulare* gen. et sp. nov., petrified seeds from the Calciferous Sandstone Series of Berwickshire. *Transactions of the Royal Society, Edinburgh* **64**, 261–284.

Long, A. G. (1960*c*). *Stamnostoma huttonense* gen. et sp. nov. — a pteridosperm seed and cupule from the Calciferous Sandstone Series of Berwickshire. *Transactions of the Royal Society, Edinburgh* **64**, 201–215.

Long, A. G. (1961*a*). On the structure of *Deltasperma fouldenense* gen. et sp. nov., and *Camptosperma berniciense* gen. et sp. nov., petrified seeds from the Calciferous Sandstone Series of Berwickshire. *Transactions of the Royal Society, Edinburgh* **64**, 281–295.

Long, A. G. (1961*b*). Some pteridosperm seeds from the Calciferous Sandstone Series of Berwickshire. *Transactions of the Royal Society, Edinburgh* **64**, 401–419.

Long, A. G. (1965). On the cupule structure of *Eurystoma angulare*. *Transactions of the Royal Society of Edinburgh* **66**, 111–129.

Long, A. G. (1975). Further observations on some Lower Carboniferous seeds and cupules. *Transactions of the Royal Society, Edinburgh* **69**, 267–293.

Long, A. G. (1977*a*). Observations on Carboniferous seeds of *Mitrospermum*, *Conostoma* and *Lagenostoma*. *Transactions of the Royal Society, Edinburgh* **70**, 37–61.

Long, A. G. (1977*b*). Some Lower Carboniferous pteridosperm cupules bearing ovules and microsporangia. *Transactions of the Royal Society of Edinburgh* **70**, 1–11.

Long, A. G. (1979). Observations on the Lower Carboniferous genus *Pitu*s Witham. *Transactions of the Royal Society, Edinburgh* **70**, 111–127.

Mamay, S. H. (1954). *Two New Plant Genera of Pennsylvanian Age from Kansas Coal Balls*. Geological Survey Professional Paper No. 254D, United States Government Printing Office, Washington.

Mapes, G. & Rothwell, G. W. (1984). Permineralized ovulate cones of *Lebachia* from Late Palaeozoic limestones of Kansas. *Palaeontology* **27**, 69–94.

Mapes, G. & Rothwell, G. W. (1991). Structure and relationships of a primitive conifer. *Neues Jahrbüch Geologie und Paläontologie* **183**, 269–287.

Matten, L. C. (1992). Reconstruction of *Laceya*, an Upper Devonian seed plant. In Abstracts of the Fourth International Organisation of Palaeobotany Conference, Paris, p. 105.

Matten, L. C., Fine, T. I., Tanner, W. R. & Lacey, W. S. (1984). The megagametophyte of *Hydrasperma tenuis* Long from the Upper Devonian of Ireland. *American Journal of Botany* **71**, 1461–1464.

Matten, L. C. & Hopkins, W. M. (1967). *Hexaptospermum delevoryii* from the Middle Pennsylvanian of Southern Illinois. *Transactions of the Illinois State Academy of Science* **60**, 98–99.

Matten, L. C., Lacey, W. S. & Lucas, R. C. (1980). Studies on the cupulate seed genus *Hydrasperma* Long from Berwickshire and East Lothian in Scotland and County Kerry in Ireland. *Botanical Journal of the Linnean Society* **81**, 249–273.

McLoughlin, S., Bomfleur, B. & Drinnan, A. N. (2018). *Pacytestopsis tayloriorun* gen. et sp. nov., an anatomically preserved glossopteris seed from the Lopingian of Queensland, Australia. In Krings, M., Harper, C. J., Cúneo, N. R. & Rothwell, G. W. (eds). *Transformative Paleobotany: Papers to Commemorate the Life and Legacy of Thomas N. Taylor,* pp. 155–178*.* Academic Press, London.

McLoughlin, S., Maksimenko, A. & Mays, S. (2019). A new high-palaeolatitude late Permian permineralized peat flora from the Sydney Basin, Australia. *International Journal of Plant Sciences* **180** (suppl.), 513–539.

Meade, L. E., Plackett, A. R. G. & Hilton, J. (2021). Reconstructing development of the earliest seed integuments raises a new hypothesis for the evolution of ancestral seed-bearing structures. *New Phytologist* **229**, 1782–1794.

Neely, F. E. (1951). Small petrified seeds from the Pennsylvanian of Illinois. *Botanical Gazette* **113**, 165–179.

Neregato, R., D’Apolito, C., Glasspool, I. J., Wang, S. J., Feng, L., Windslow, P., Lu, J., Shao, L., & Hilton, J. (2016). Palynological constraints on the provenance and stratigraphic range of a Lopingian (late Permian) inter-extinction floral lagerstätte from the Xuanwei Formation, Guizhou Province, China. *International Journal of Coal Geology* **62**, 139–150.

Nishida, H., Pigg, K. B., Kudo, K. & Rigby, J. F. (2007). New evidence of reproductive organs of *Glossopteris* based on permineralized fossils from Queensland, Australia. I. Ovulate organs of *Homevaleia* gen. nov. *Journal of Plant Research* **120**, 539–549.

Nishida, H., Pigg, K. B. & Rigby, J. F. (2003). Swimming sperm in an extinct Gondwanan plant. *Nature* **422**, 396–397.

Nishida, H., Pigg, K. B. & Rigby, J. F. (2004). Zooidogamy in the Late Permian genus *Glossopteris*. *Journal of Plant Research* **117**, 323–328.

Oliver, F. W. (1904). On the structure and affinities of *Stephanospermum* Brongniart, a genus of fossil gymnosperm seeds. *Transactions of the Linnean Society London, Botany* **6**, 361–400.

Oliver, F. W. (1909). On *Physostoma elegans*, Williamson, an archaic type of seed from the Palaeozoic rocks. *Annals of Botany* **23**, 73–116.

Oliver, F. W. & Salisbury, E. J. (1911). On the structure and affinities of the Palaeozoic seeds of the *Conostoma* group. *Annals of Botany* **25**, 1–50.

Oliver, F. W. & Scott, D. H. (1904). On the structure of the Palaeozoic seed *Lagenostoma lomaxi*, with a statement of the evidence upon which it is referred to *Lyginodendron*. *Philosophical Transactions of the Royal Society, London, Biology* **197**, 193–247.

Phillips, T. L. (1980). Stratigraphic and geographic occurrences of permineralized coal-swamp plants: Upper Carboniferous of North America and Europe. In Dilcher, D. L. & Taylor, T. N. (eds), *Biostratigraphy of Fossil Plants: Successional and Paleoecological Analyses*, pp. 25–91. Dowden, Hutchinson & Ross, Stroudsburg, Pennsylvania.

Prankerd, T. L. (1912). On the structure of the Palaeozoic seed *Lagenostoma ovoides*, Will. *Journal of the Linnean Society of London, Botany* **40**, 461–490.

Raymond, A. & McCarty, R. (2009). *Pachytesta crenulata*, a new medullosan ovule from the Pennsylvanian of Iowa. *Review of Palaeobotany and Palynology* **156**, 283–306.

Retallack, G. J. & Dilcher, D. L. (1988). Reconstructions of selected seed ferns. *Annals of the Missouri Botanical Garden* **75**, 1010–1057.

Roth, E. (1955). The anatomy and modes of preservation of the genus *Cardiocarpus spinatus* Graham. *University of Kansas Science Bulletin* **38**, 151–174.

Rothwell, G. W. (1971*b*). Ontogeny of the Paleozoic ovule, *Callospermarion pusillum*. *American Journal of Botany* **58**, 706–715.

Rothwell, G. W. (1975). The Callistophytaceae (Pteridospermopsida). I. Vegetative structures. *Palaeontographica B, Palaeophytologie* **151**, 171–196.

Rothwell, G. W. (1980). The Callistophytaceae (Pteridospermopsida). II. Reproductive features. *Palaeontographica B, Palaeophytologie* **173**, 85–106.

Rothwell, G. W. (1981). The Callistophytales (Pteridospermopsida): reproductively sophisticated Paleozoic gymnosperms. *Review of Palaeobotany and Palynology* **32**, 103–121.

Rothwell, G. W. (1982*a*). *Cordianthus duquesnensis* sp. nov., anatomically preserved ovulate cones from the Upper Pennsylvanian of Ohio. *American Journal of Botany* **69**, 239–247.

Rothwell, G. W. (1986). Classifying the earliest gymnosperms. In *Systematic and Taxonomic Approaches in Palaeobotany* (eds R. A. Spicer and B. A. Thomas), pp. 137–162. Systematics Association Special Volume No. 31, Clarendon Press, Oxford.

Rothwell, G. W. (1993). *Cordaixylon dumusum* (Cordaitales). II. Reproductive biology, phenology, and growth ecology. *International Journal of Plant Sciences* **154**, 572–586.

Rothwell, G. W. & Eggert, D. A. (1970). A *Conostoma* with tentacular sarcotesta from the upper Pennsylvanian of Illinois. *Botanical Gazette* **131**, 359–366.

Rothwell, G. W., Mapes, G. & Mapes, R. H. (1996). Anatomically preserved vojnovskyalean seed plants in Upper Pennsylvanian (Stephanian) marine shales of North America. *Journal of Paleontology* **70**, 1067–1079.

Rothwell, G. W., Scheckler, S. E. & Gillespie, W. H. (1989). *Elkinsia* gen. nov., a Late Devonian gymnosperm with cupulate ovules. *Botanical Gazette* **150**, 170–189.

Rothwell, G. W. & Scott, A. C. (1992). *Stamnostoma oliveri*, a gymnosperm with systems of ovulate cupules from the Lower Carboniferous (Dinantian) floras at Oxroad Bay, East Lothian, Scotland. *Review of Palaeobotany and Palynology* **72**, 273–284.

Rothwell, G. W. & Serbet, R. (1992). Pollination biology of *Elkinsia polymorpha*: implications for the origin of the gymnosperms. *Courier Forschungs-Institut Senckenburg* **147**, 225–231.

Rothwell, G. W. & Warner, S. (1984). *Cordaixylon dumusum* n.sp. (Cordaitales). I. Vegetative structures. *Botanical Gazette* **145**, 275–291.

Rothwell, G. W. & Wight, D. (1989). *Pullaritheca longii* gen. nov. and *Kerryia mattenii* gen. et sp. nov., Lower Carboniferous cupules with ovules of the *Hydrasperma tenuis*-type. *Review of Palaeobotany and Palynology* **60**, 295–309.

Ryberg, P. E. (2010). *Lakkosia kerasata* gen. et sp. nov., a permineralized megasporangiate glossopterid structure from the Central Transantarctic Mountains, Antarctica. *International Journal of Plant Sciences* **171**, 332–244.

Ryberg, P. E. & Taylor, E. L. (2013). *Lonchiphyllum aplospermum* gen. et sp. nov., an anatomically preserved glossopterid megasporophyll from the Upper Permian of Skaar Ridge, Transantarctic Mountains, Antarctica. *International Journal of Plant Sciences* **174**, 396–405.

Scott, A. C., Hilton, J., Galtier, J. & Stampanoni, M. (2019). A charcoalified ovule adapted for wind dispersal and deterring herbivory from the Late Viséan (Carboniferous) of Scotland. *International Journal of Plant Science* **180**, 1059–1074.

Scott, D. H. & Maslen, A. J. (1906). Note on the structure of *Trigonocarpon olivaeforme. Annals of Botany* **20**, 109–112.

Scott, D. H. & Maslen, A. J. (1910). On *Mesoxylon*, a new genus of Corditales—preliminary note. *Annals Botany* **24**, 236–239.

Segal, R. H. (1969). A re-examination of the Carboniferous fossil *Nucellangium glabrum*. *American Midland Naturalist* **81**, 272–276.

Serbet, R. & Rothwell, G. W. (1992). Characterizing the most primitive seed ferns. I. A reconstruction of *Elkinsia polymorpha. International Journal of Plant Sciences* 153, 602–621.

Serbet, R. & Rothwell, G. W. (1995). Functional morphology and homologies of gymnospermous ovules: evidence from a new species of *Stephanospermum* (Medullosales). *Canadian Journal Botany* **73**, 650–661.

Serlin, B. S. (1982). On the structure of *Cardiocarpus tritolopus*, a cordaite seed from the lower–middle Pennsylvanian of Kentucky. *Review of Palaeobotany and Palynology* **36**, 297–304.

Seward, A. C. (1917). *Fossil Plants, a Text-Book for Students of Botany and Geology*, volume 3, 1st edn. Cambridge University Press, Cambridge.

Seyfullah, L. J., Hilton, J., Liang, M.-M. & Wang, S.-J. (2010). Resolving the systematic and phylogenetic position of isolated ovules: a case study on a new genus from the Permian of China. *Botanical Journal of the Linnean Society* **164**, 84–108.

Spencer, A. R. T., Wang, S.-J., Dunn, M. T. & Hilton, J. (2013*b*). Species of the medullosan ovule *Stephanospermum* from the Lopingian (late Permian) floras of China. *Journal of Asian Earth Sciences* **76**, 59–69.

Stewart, W. N. (1954). The structure and affinities of *Pachytesta illinoense* comb. nov. *American Journal of Botany* **41**, 500–508.

Stidd, B. M. & Cosentino, K. (1976). *Nucellangium*: gametophytic structure and relationship to cordaites. *Botanical Gazette* **137**, 242–249.

Stidd, B. M. & Hall, J. W. (1970). The natural affinity of the Carboniferous seed, *Callospermarion*. *American Journal of Botany* **57**, 827–836.

Stubblefield, S. P. & Rothwell, G. W. (1980). *Conostoma chappellicum* n. sp., lagenostomalean ovules from Kentucky. *Journal of Paleontology* **54**, 1012–1016.

Taylor, T. N. (1965). Paleozoic seed studies: a monograph of the American species of *Pachytesta*. *Palaeontographica B, Palaeophytologie* **117**, 1–46. pls 1–22.

Taylor, T. N. (1966). Paleozoic seed studies: on the genus *Hexapterospermum*. *American Journal of Botany* **53**, 185–192.

Taylor, T. N. (1967). Paleozoic seed studies: on the structure of *Conostoma leptospermum* n. sp., and *Albertlongia incostata* n. gen. and sp. *Palaeontographica B, Palaeophytologie* **121**, 23–29.

Taylor, T. N. & Delevoryas, T. (1964). Paleozoic seed studies: a new Pennsylvanian *Pachytesta* from southern Illinois. *American Journal of Botany* **51**, 189–195.

Taylor, T. N. & Eggert, D. A. (1967). Petrified plants from the Upper Mississippian of North America. I: the seed *Rhynchosperma* gen. nov. *American Journal of Botany* **55**, 306–313.

Taylor, T. N. & Stewart, W. N. (1964). The Paleozoic seed *Mitrospermum* in American coal balls. *Palaeontographica B, Palaeophytologie* **115**, 51–58, pls. 17–20.

Taylor, T. N. & Taylor, E. L. (1987). Structurally preserved fossil plants from Antarctica. III. Permian seeds. *American Journal of Botany* **74**, 904–913.

Trivett, M. L. (1992). Growth architecture, structure, and relationships of *Cordaixylon iowensis* nov. comb. (Cordaitales). *International Journal of Plant Sciences* **153**, 273–287.

Trivett, M. L. & Rothwell, G. W. (1985). Morphology, systematics, and paleoecology of Paleozoic fossil plants: *Mesoxylon priapi*, sp. nov. (Cordaitales). *Systematic Botany* **10**, 205–223.

Trivett, M. L. & Rothwell, G. W. (1991). Diversity among Palaeozoic Cordaitales. *Neue Jahrbuch für Geologie und Paläontologie Abhbeiulung* **183**, 289–305.

Wang, S.-J. (1997). A study on origin and evolution of Cordaitaceae in Late Palaeozoic. *Acta Phytotaxonica Sinica* **35**, 303–319.

Wang, S.-J., Hilton, J. & Galtier, J. (2003*a*). Cordaitalean seed plants from the Early Permian of North China. I. Delimitation and reconstruction of the *Shanxioxylon sinense* plant. *International Journal of Plant Sciences* **164**, 89–112.

Wang, S.-J., Hilton, J., Liang, M. & Stevens, L. (2006). Permineralized seed plants from the Late Permian of southern China: a new species of *Cardiocarpus*. *International Journal of Plant Sciences* **167**, 1247–1257.

Wang, S.-J., Hilton, J. & Tian, B. (2003*b*). A new species of permineralised cardiocarpalean ovule from the Early Permian Taiyuan Formation of northern China. *Review of Palaeobotany and Palynology* **123**, 303–319.

Wang, S.-J. & Tian, B.-L. (1991). A new species of petrified ovules of Late Paleozoic. *Acta Botanica Sinica* **33**, 958–962, pl 1.

Willard, D. A., Phillips, T. L., Lesnikowska, A. D. & DiMichele, W. A. (2007). Paleoecology of the Late Pennsylvanian-age Calhoun coal bed and implications for long-term dynamics of wetland ecosystems. *International Journal of Coal Geology* **69**, 21–54.

Williamson, W. C. (1877). On the organisation of the fossil plants of the coal measures, Part VIII. Ferns (continued) and gymnospermous stems and seeds. *Philosophical Transactions of the Royal Society, London* **167**, 271–312.
