# Supplementary material for: Early evolutionary history of the seed
Source: Biol Rev Camb Philos Soc. 2026 Jan 26;101(3):1511–53. doi: 10.1002/brv.70134 (PMC13149795; doi:10.1002/brv.70134)
Supplement: Supplementary file 2 — Appendix S2. Details of scored characters. [file BRV-101-1511-s002.docx]

**Appendix S2. Details of Scored Characters**

Tree-Building Characters

**1. Ovule symmetry.** Re-ordered from Character 1 in Hilton *et al.* (2003), Character 1 in Seyfullah *et al.* (2010), equivalent to Character 41 in Rothwell & Serbet (1994). Character reflects the synopsis of Palaeozoic ovule symmetry by Rothwell (1986).

0 Radial

1 Bilateral

2 180° rotational

**2. Ovule base.** Re-ordered from Character 3 in Hilton *et al.* (2003), Character 3 in Seyfullah *et al.* (2010). Character traditionally used to identify ovules of cordaitean plants but here viewed as a distinct chalazal morphology that is present in a diverse range of ovule-species.

0 Non-cordate

1 Cordate

**3. Micropyle.** New character.

0 Absent (pre-integumentary lobes not convergent)

1 Present, broad opening

2 Present, narrow opening

**4. Integumentary lobe fusion.** New character.

0 Lobes ± free (fusion 1–33%)

1 Moderately fused (34–66%)

2 Strongly fused (>67%)

3 Lobes absent

**5. Integument lobes greatly exceed the length of nucellar apex.** New character. Character designed to group hydrasperman ovules, including *Genomosperma kidstonii* (Long, 1960*b*), *Salpingostoma dasu* (Gordon, 1942) and *Tantallosperma setigera* (Barnard & Long, 1973; Bateman & Rothwell, 1990), in which integumentary lobes extend well beyond the nucellar apex. It has been argued that in these ovule-species the protruding integumentary lobes could potentially affect pollination differently from other taxa in which the integument is much shorter (e.g. Niklas, 1981).

0 Lobes absent or less than three times the length of the nucellar apex

1 Lobes present, at least three times the length of the nucellar apex

**6. Integumentary lobes terete in transverse section.** New character, based on observations by the authors on the morphology of hydrasperman ovule integuments.

0 Present

1 Absent

**7. Longitudinal grooves and ridges below free integumentary lobes.** New character, based on observations by Long (1975, p. 275) that species of *Tantallosperma*, *Salpingostoma* and *Dolichosperma* have prominent ridges from the chalaza that develop apically into free integumentary lobes.

0 Absent

1 Present

**8. Central concavity in minor plane.** Character 4 in Hilton *et al.* (2003), Character 4 in Seyfullah *et al.* (2010). Represents an inward-facing depression in the minor plane when observed in transverse section, such as those documented in *Cardiocarpus dabiziae* and *Cycadinocarpus augustodunensis*.

0 Absent

1 Present

**9. Commissure.** Expanded from Character 5 in Seyfullah *et al.* (2010) to recognise State 2 for commissured ribs, in which the commissures of some trigonocarpalean ovules occur in the same plane as integumentary ribs and bisect them longitudinally.

0 Absent

1 Present

2 Commissured ribs

**10. Nucellar fusion to integument.** Character 6 in Hilton *et al.* (2003), Character 6 in Seyfullah *et al.* (2010).

0 Extends distally from chalaza

1 Confined to chalaza

**11. Number of nucellar bundles.** New character (autapomorphic to *Conostoma chappellicum*).

0 One

1 Two (autapomorphic)

**12. Laterally extensive vascular pad/bowl below base of nucellus.** Character 7 in Hilton *et al.* (2003), Character 7 in Seyfullah *et al.* (2010).

0 Absent

1 Present

**13. Nucellar bundle/pad/platform protrudes into nucellar cavity.** New character, based on species in which the nucellus stands proud from the base of the nucellar cavity (as seen in some species of cardiocarpalean and trigonocarpalean ovules); absent from hydrasperman ovules.

0 Absent

1 Present

**14. Vascularised nucellar sheath.** Character 8 in Hilton *et al.* (2003), Character 8 in Seyfullah *et al.* (2010).

0 Absent

1 Present, proximal sheath

2 Present, distal sheath

**15. Course of vascular bundles arising from nucellar pad.** Reworded from Character 9 in Seyfullah *et al.* (2010) to distinguish this character more clearly from Character 15.

0 Absent

1 Present, passing from nucellus into integument

2 Present, confined to nucellus

**16. Organisation of vascular bundles in the nucellus.** New character.

0 Absent

1 Present, organised radially

2 Present, organised bilaterally in major plane

**17. Integumentary bundles depart from nucellar bundle.** Re-ordered from Character 10 in Seyfullah *et al.* (2010). Taxa lacking sclerotesta scored as Not Applicable (–).

0 Before entering sclerotesta

1 Within sclerotesta

2 After passing through sclerotesta

3 Absent from sclerotesta

**18. Integumentary bundles pass through sclerotesta.** Expanded from Character 10 in Hilton *et al.* (2003) and Character 11 in Seyfullah *et al.* (2010) to include State 3, recognising additional variation among trigonocarpalean-type ovules in which the integumentary bundles pass between basally orientated crenulations of the sclerotesta collar/projections.

0 Once

1 Twice

2 Do not pass through sclerotesta

3 Between chalazal sclerotesta collar/projections

4 Bundles absent

**19. Integumentary bundle organisation.** Expanded and re-ordered from Character 12 in Seyfullah *et al.* (2010) to add state (3) for species lacking integumentary vascularisation, and (4) for *Conostoma* *elliptica* (ovules of the *Gnetopsis elliptica* cupule) that has paired bundles to each side of the major plane but orientated in the minor plane. This character distinguishes the integumentary vascularisation of platyspermic (bilateral and 180° rotational symmetry) from radially symmetrical ovules, and recognises that cardiocarpalean ‘platyspermic’ ovules can either have two bundles with one to each side of the ovule in the major plane, or multiple bundles to each side of the ovule in the major plane as in species of *Mitrospermum*.

0 Radially organised

1 Paired in major plane

2 Multiple bundles in major plane

3 Absent

4 Paired bundles in minor plane to either margin in major plane (autapomorphic)

**20. Proximal/chalazal position of integumentary bundles.** Re-ordered from Character 13 in Seyfullah *et al.* (2010). Character distinguishes hydrasperman-type ovules in which integumentary bundles tend to occur within the endotesta from other kinds of ovule in which they tend to be located in the sclerotesta or sarcotesta, or wholly absent. In the present data set, State 2 is autapomorphic to the Permian glossopterid *Chaonostoma verruculosum* (Klavins *et al*., 2001).

0 Within endotesta

1 Within sclerotesta or sarcotesta

2 Absent (autapomorphic)

**21. Integumentary bundles deeply descending and recurrent.**Character 11 in Hilton *et al.* (2003), Character 14 in Seyfullah *et al.* (2010), based on the recurrent bundles of Brongniart (1881, p. 21) and as elaborated by Bertrand in *Diplotesta* (Bertrand, 1907*c*) and *Rhabdocarpus* (Bertrand, 1907*b*) who compared different vascular configurations within cardiocarpalean ovules.

0 Absent

1 Present

**22. Integumentary bundle tracheids.** Re-ordered from Character 15 in Seyfullah *et al.* (2010).

0 Less than 20

1 More than 20

2 Absent

**23. Integument with trichomes on exterior surface.** New character.

0 Absent

1 Present

**24. Integumentary trichomes on exterior surface organised in vertical files.** New character.

0 Absent

1 Present

**25. Integument with trichomes on interior surface.** New character.

0 Absent

1 Present

**26. Integumentary trichome density on exterior surface.** New character.

0 Absent

1 Sparse, distance between adjacent trichomes longer than trichome length

2 Dense, distance between adjacent trichomes shorter than trichome length

**27. Integumentary trichomes with radially expanded glandular apex.** New character.

0 Absent

1 Present

**28. Integumentary trichome length.** New character.

0 Absent

1 Short, less than twice length of adjacent epidermal cells

2 Long, more than twice length of adjacent epidermal cells

**29. Epidermis with columnar cells.** New character.

0 Absent

1 Present, secretory or mucilage filled

2 Present, lack cell contents

**30. Sarcotesta.** New character recognising the presence of a distinct parenchymatous sarcotesta external to a sclerotesta (as scored, sarcotesta is only recognised in ovules with a sclerotesta; ovules lacking sclerotesta comprise only endotesta plus epidermis).

0 Absent

1 Present

**31. Sarcotestal longitudinal ribs.** Character 16 in Seyfullah *et al.* (2010).

0 Absent

1 In major plane only

2 In radial organisation

**32. Sarcotestal wing in major plane.** Re-ordered from Character 15 in Hilton *et al.* (2003), Character 17 in Seyfullah *et al.* (2010).

0 Absent

1 Well developed

2 Weakly developed

**33. Sarcotesta with irregular (including undulate or tuberculate) outer surface.** Character 18 in Seyfullah *et al.* (2010).

0 Absent

1 Present

**34. Sarcotestal layering.** Character 17 in Hilton *et al.* (2003), Character 19 in Seyfullah *et al.* (2010).

0 Absent

1 Present

**35. Sarcotesta with thick walls.** Re-ordered from Character 18 in Hilton *et al.* (2003), Character 20 in Seyfullah *et al.* (2010).

0 Absent

1 Present

**36. Sarcotestal cells radially expanded.** Re-ordered from Character 19 in Hilton *et al.* (2003), Character 21 in Seyfullah *et al.* (2010).

0 Absent

1 Present

**37. Sarcotesta with secretory cells or mucilage-filled cells.** Character 20 in Hilton *et al.* (2003), Character 22 in Seyfullah *et al.* (2010).

0 Absent

1 Present

**38. Sarcotestal secretory/mucilage-filled cells larger than surrounding cells.** Re-worded from Character 21 in Hilton *et al.* (2003) and Character 23 in Seyfullah *et al.* (2010).

0 Absent

1 Isolated

2 Aggregated into secretory canals

**39. Large spherical–ovoid glands within sarcotesta.** Character 22 in Hilton *et al.* (2003), Character 24 in Seyfullah *et al.* (2010).

0 Absent

1 On inner surface only

2 On outer surface only

**40. Sarcotesta with long, unvascularised apical projections.** New character recognising presence of apical sarcotestal extensions in ovules of the *Conostoma*-type contained within *Gnetopsis elliptica* cupules (Galtier, 2013).

0 Absent

1 Present (autapomorphic)

**41. Sclerotesta.** New character recognising presence of distinct tissue layer composed of sclereids.

0 Absent

1 Present

**42. Sclerotesta internal layering.** Character 24 in Hilton *et al.* (2003), Character 25 in Seyfullah *et al.* (2010).

0 Absent

1 Present, indistinct

2 Present, distinct

**43. Sclerotesta with pitted sclereids.** Character 26 in Hilton *et al.* (2003), Character 26 in Seyfullah *et al.* (2010).

0 Absent

1 Present

**44. Sclerotesta with elongated cells.** Character 27 in Hilton *et al.* (2003), Character 27 in Seyfullah *et al.* (2010).

0 Absent

1 Present

**45. Sclerotesta with secretory or mucilage-filled cells.** New character following variation noted in *Emporia cryptica* (Hernandez-Castillio *et al*., 2009).

0 Absent

1 Present (autapomorphic)

**46. Sclerotesta thickness.** New character, recognising variation in degree of sclerotestal development through the ovule.

0 Sclerotesta absent

1 Uniform

2 Thickness increases distally

3 Thickness decreases distally

4 Thickness decreases around mid-point only.

**47. Sclerotesta external surface texture.** New character for seeds with sclerotesta that is textured (i.e. excluding ribs).

0 Sclerotesta absent

1 Smooth

2 Irregular

**48. Sclerotesta internal surface texture.** New character recognising variation in the micromorphology of the inner wall of the sclerotesta; the irregular condition is autapomorphic to *Albertlongia incostata*, and the Sclerotesta Absent state is itself absent from the present matrix after applying optimisation.

0 Sclerotesta absent [not applicable]

1 Smooth

2 Irregular (autapomorphic)

**49. Sclerotestal ribs in major plane.** Character 29 in Hilton *et al.* (2003), Character 49 in Seyfullah *et al.* (2010).

0 Absent

1 Bilateral organisation

2 Radial organisation

**50. Sclerotestal rib with lateral gutters.** New character based on some cardiocarpalean ovules in which sclerotestal ribs in the major plane have bilaterally lateral projections that Bertrand (1907*c*) termed “*guttières carénales de la coque*” (“keels with lateral gutters”) and Doubinger *et al*. (1995, p. 82) regarded as “lateral gutters”. This feature, of unknown function, is evident in figures of *Cycadinocarpus augustodunensis* and *Leptocaryum avellanum* as illustrated by Brongniart (1881), and is mentioned by Bertrand (1907*c*) and Doubinger *et al*. (1995) for *Taxospermum grunerii*.

0 Absent

1 Present

**51. Sclerotestal rib in minor plane.** Expanded from Character 30 in Seyfullah *et al.* (2010) to segregate state 1 (Present) into (1) Present, entire length of ovule, and (2) Present, proximal only. This character relates to variation observed in platyspermic ovules in which species of *Mitrospermum* have a rib in the minor plane though their entire length whereas species of *Diplotesta* and *Cardiocarpus sclerotesta* have prominent ribs in the minor plane in the proximal part of the ovule only. The platyspermic hydrasperman-type ovule *Deltasperma fouldenense* is scored as (2), recognising its integumentary rib expressed in the minor plane.

0 Absent

1 Present, entire length of ovule

2 Present, proximal only

**52. Sclerotesta apical projections/teeth.** New character, defined as differentiated tissue teeth/projections circumscribing the exterior apical region (distal to the level of the pollen chamber).

0 Absent

1 Present, lacking secondary teeth/projections

2 Present, also possessing secondary teeth/projections

**53. Sclerotesta apical collar.** New character, defined as an unbroken ring of tissue circumscribing the exterior apical region (distal to the level of the pollen chamber); it may partly consist of undifferentiated teeth/projections.

0 Absent

1 Present

**54. Sclerotesta buttress supporting salpinx/nucellar beak.** Character excluded by Seyfullah *et al.* (2010) as autapomorphic to *Stephanospermum tridentatum* but here included as linking *S. tridentatum* with *S. elongatum*.

0 Absent

1 Present

**55. Sclerotesta tubular salpinx/nucellar beak surrounding micropylar canal.** New character to incorporate sclerotesta tubular projection that surrounds micropylar canal and projects upwards; short = same height or lower than any apical collar or projections; long = exceeding any apical collar or projections.

0 Absent

1 Short

2 Long

**56. Sclerotesta basal collar/projections.** Character 31 in Seyfullah *et al.* (2010).

0 Absent

1 Discontinuous longitudinal ribs

2 Continuous

**57. Large air chamber below nucellus.** New character, defined as a chamber formed by sclerotesta that is basal to the nucellus.

0 Absent

1 Present

**58. Chalazal skirt encircling nucellus.** Character 32 in Seyfullah *et al.* (2010).

0 Absent

1 Present

**59. Endotesta.** Re-ordered and expanded from Character 33 in Seyfullah *et al.* (2010) to include State (3) for hydrasperman taxa lacking a differentiated endotesta.

0 Biseriate–multiseriate

1 Uniseriate

2 Thin cuticle proximally, biseriate apically

3 Absent

**60. Endotesta with secretory cells.** New character.

0 Absent

1 Present

**61. Endotesta secretory cells larger than surrounding cells.** New character, autapomorphic for *Elkinsia polymorpha*.

0 Absent

1 Present (autapomorphic)

**62. Distally elongated endotesta cells.** Character 33 in Hilton *et al.* (2003), Character 34 in Seyfullah *et al.* (2010).

0 Absent

1 Present

**63. Distal apex of functional megaspore.** Reworded from Character 35 in Seyfullah *et al.* (2010).

0 Rounded

1 With central tent pole/domed apical projection

**64. Pollen chamber size.** Character 36 in Hilton *et al.* (2003), Character 37 in Seyfullah *et al.* (2010). State (2) was included as an autapomorphy of *Coumiasperma remyi* (Galtier & Rowe, 1989) – an ambiguous taxon subsequently omitted from our analysis.

0 Large

1 Small

[2 Absent (autapomorphic)]

**65. Pollen chamber shape.** Character 38 in Seyfullah *et al.* (2010). State (2) was included as an autapomorphy of *Coumiasperma remyi* (Galtier & Rowe, 1989) – an ambiguous taxon subsequently omitted from our analysis.

0 Hemispherical dome

1 Bell- or funnel-shaped

[2 Absent (autapomorphic)]

**66. Hydrasperman-type pollen chamber length:width ratio.** New character based on size distributions of pollen chamber length and width measurements.

0 Less than 5

1 Equal to or greater than 5

**67. Membranous pollen chamber floor.** Character 37 in Hilton *et al.* (2003), Character 40 in Seyfullah *et al.* (2010).

0 Present

1 Absent

**68. Salpinx morphology.** New character segregated from Character 38 of Seyfullah *et al.* (2010).

0 Narrow, tubular

1 Intermediate

2 Broad, open

3 Absent

**69. Salpinx length:width ratio.** New character based on size distributions of salpinx length and width measurements.

0 Less than 1.5

1 Equal to or greater than 1.5

**70. Central column.** Character 38 in Hilton *et al.* (2003), Character 41 in Seyfullah *et al.* (2010).

0 Present

1 Absent

**71. Nucellar beak extending to micropyle.** Segregated from Character 38 of Seyfullah *et al.* (2010).

0 Absent

1 Present

**72. *Sphaerostoma*-type nucellar ontogeny.** New character based on ontogenetic development pattern in the nucellar apices of some hydrasperman ovules, first recognised by Benson (1914) in *Sphaerostoma* (discussed by Scott *et al.*, 2019).

0 Absent

1 Present

**73. Post-pollination sealing of the megagametophyte by the central column.** Character 39 in Hilton *et al.* (2003), Character 42 in Seyfullah *et al.* (2010).

0 Present

1 Absent

**74. Post-pollination sealing of the megagametophyte by the nucellar apex.** Character 40 in Hilton *et al.* (2003), Character 43 in Seyfullah *et al.* (2010).

0 Absent

1 Present

**75. Post-pollination sealing of the megagametophyte by integument.** Character 41 in Hilton *et al.* (2003), Character 44 in Seyfullah *et al.* (2010).

0 Absent

1 Present

**76. One ovule per cupule.** New character.

0 Absent

1 Present

**77. Two or more ovules per cupule.** New character.

0 Present

1 Absent

**78. Overtopping amongst ovules within cupule.** New character describing patterns of ovule insertion within cupules that deviate from a single horizontal plane.

0 Absent

1 Present

**79. Cupule lobe fusion.** New character describing the proximal–distal lateral fusion of cupule lobes.

0 Free lobes (fusion 1–33%)

1 Intermediately fused lobes (fusion 34–67%)

2 Highly fused lobes (fusion >67%)

3 Cupule absent

**80. Cupule symmetry in transverse section.** New character based on variation in the architecture of hydrasperman cupules bearing ovules of the *Hydrasperma*-type.

0 Symmetrical

1 Asymmetrical

**81. Cupule with glandular bodies.** New character autapomorphic to the archetypal pteridosperm, *Lagenostoma lomaxii* (Oliver & Scott, 1904).

0 Absent

1 Present (autapomorphic)

**82. Cupule with trichomes on exterior surface.** New character reflecting variation in hydrasperman-type cupules that typically lack hairs, whereas cupules of *Calathospermum fimbriatum* bearing *Salpingostoma dasu* ovules and *Gnetopsis elliptica* bearing *Conostoma*-type ovules have trichomes on both internal and external cupule surfaces (Galtier, 2013).

0 Absent

1 Present

**83. Ovules borne on extended sporophylls aggregated as cone.** New character.

0 Absent

1 Present

**84. Ovules borne terminally on dichotomous branching system.** New character, autapomorphic to *Stephanospermum konopeonus*.

0 Absent

1 Present (autapomorphic)

**85. Ovules attached directly to leaf or leaf homologue (sporophyll).** New character reflecting variation in ovule position on a leaf with either abaxial ovules (Callistophytales; Rothwell, 1981) or adaxial (some glossopterids, including *Homevaleia gouldii*; Nishida *et al*., 2007), or lateral to the laminar part of the leaf (some cycads).

0 Absent

1 Present, abaxial

2 Present, adaxial

**86. (Pre)Pollen structure.** New character

0 Proximal tetrad scar

1 Distal sulcus or circular germination area

**87. (Pre)Pollen symmetry.** New character

0 Radial

1 Bilateral

**88. (Pre)Pollen sacs.** New character

0 Non-saccate or subsaccate

1 Saccate

**89. (Pre)Pollen infratectal structure.** New character

0 Massive or spongy alveolar

1 Honeycomb alveolar

Excluded Characters

These characters remain in the associated data-matrix (Data S1) as C90 and C91

**90. Cellular megagametophyte.** We assessed as a potential character the contrasting cellular patterns in megagametophytes noted in Palaeozoic ovules by Rothwell (1971*a*), Grove & Rothwell (1980) and Serlin (1982). However, tissues of the megagametophyte change during ontogeny, and in any case they are found only in the best-preserved specimens.

0 Absent, free cellular

1 Present, alveolar

2 Present, cellular megagametophyte

**91. Hydrasperman-type pollen chamber length.** New character based on size distributions of pollen chamber lengths. Eventually judged to be too vulnerable to ontogenetic effects.

0 Less than 0.4 mm

1 0.4–0.8 mm

2 Equal to or greater than 0.8 mm

Mapped Characters

Three features extrinsic to the ovule-species were also scored, but were withheld for *post-hoc* ‘mapping’ across the topologies previously constructed entirely from intrinsic characters. These mapped properties (not strictly characters) remain in the associated data matrix (Data S1) as C92–C94.

**92. Depositional environment.** Virtually all fossil seeds are transported at least locally, but nonetheless offer useful hints regarding the taphonomic cycle of life-habitat, transport medium, depositional environment and subsequent preservation.

0 Deltaic/estuarine/paralic settings

1 Volcanic (epiclastics, agglomerates, ashes)

2 Peat-forming mires

3 Lacustrine

4 Marine

**93. Permineralising medium.** Distinguishing contrasting styles of anatomical preservation.

0 Calcium carbonate – bedded calcite/dolomite

1 Calcium carbonate – coal ball

2 Iron carbonate (siderite)

3 Pyrite (pyritisation)

4 Silica (silicification)

5 Charcoal (charcoalification; inertinite)

**94. Phytogeographic realm.** Occurrence in widely recognised late Palaeozoic floral provinces (e.g. Scotese, 2021).

0 Euramerica

1 Cathaysia

2 Gondwana

REFERENCES

Bateman, R. M. & Rothwell, G. W. (1990). A reappraisal of the Dinantian floras at Oxroad Bay, East Lothian, Scotland. 1. Floristics and the development of whole-plant concepts. *Transactions of the Royal Society of Edinburgh B* **81**, 127–159.

Barnard, P. D. W. & Long, A. G. (1973). On the structure of a petrified stem and some associated seeds from the Lower Carboniferous rocks of East Lothian, Scotland. *Transactions of the Royal Society of Edinburgh* **69**, 91–108.

Benson, M. J. (1914). *Sphaerostoma ovale* (*Conostoma ovale* et *intermedium*, Williamson), a Lower Carboniferous ovule from Pettycur, Fifeshire, Scotland. *Transactions of the Royal Society, Edinburgh* **50**, 1–15.

Bertrand, M. C. E. (1907*b*). Les charactéristiques du genre *Rhabdocarpus* d’apres les préparations de la collection B. Renault. *Bulletin de la Société Botanique du France* **54**, 654–664.

Bertrand, M. C. E. (1907*c*). Les charactéristiques du genre *Taxospermum* de Brongniart. *Bulletin de la Société Botanique du France* **54**, 213–224.

Brongniart, A. (1881). *Researches sur les Graines Fossiles Silicifieés*. Masson, Paris.

Doubinger, J., Vetter, P., Langiaux, J., Galtier, J. & Broutin, J. (1995). *La Flore Fossile du Bassin Houiller de Saint-Étienne (Memoires du Museum National d’Histoire Naturelle).* Museum National d’Histoire Naturelle, Paris.

Galtier, J. (2013). Reinvestigation of the Carboniferous multiovulate cupule *Gnetopsis eliptica* and its evolutionary significance. *International Journal of Plant Sciences* **174**, 382–395.

Galtier, J. & Rowe, N. P. (1989). A primitive seed-like structure and its implications for early gymnosperm evolution. *Nature* **340**, 225–227.

Gordon, W. T. (1942). On *Salpingostoma dasu*: a new Carboniferous seed from East Lothian. *Transactions of the Royal Society, Edinburgh* **50**, 427–464.

Grove, G. G. & Rothwell, G. W. (1980). *Mitrospermum vinculum* sp. nov., a cardiocarpalean ovule from the Upper Pennsylvanian of Ohio. *American Journal of Botany* **67**, 1051–1058.

Hernandez-Castillo, G. R., Stockey, R. A., Rothwell, G. W. & Mapes, G. (2009). Reconstruction of the Pennsylvanian-age walchian conifer *Emporia cryptica* sp. nov. (Emporiaceae: Voltziales). *Review of Palaeobotany and Palynology* **157**, 218–237.

Hilton, J., Wang, S. J. & Tian, B. (2003). Reinvestigation of *Cardiocarpus minor* (Wang) Li from the Early Permian Taiyuan Formation of northern China, and an evaluation of cardiocarpalean ovule taxonomy, systematics and phylogeny. *Botanical Journal of the Linnean Society* **141**, 151–175.

Klavins, S. D., Taylor, E. L., Krings, M. & Taylor, T. N. (2001). An unusual, structurally preserved ovule from the Permian of Antarctica. *Review of Palaeobotany and Palynology* **115**, 107–117.

Long, A. G. (1960*b*). On the structure of *Samaropsis scotia* Calder (emended) and *Eurystoma angulare* gen. et sp. nov., petrified seeds from the Calciferous Sandstone Series of Berwickshire. *Transactions of the Royal Society of Edinburgh* 64, 261–284.

Long, A. G. (1975). Further observations on some Lower Carboniferous seeds and cupules. *Transactions of the Royal Society of Edinburgh* **69**, 278–293.

Niklas, K. J. (1981). Airflow patterns around some early seed plant ovules and cupules: implications concerning efficiency in wind pollination. *American* *Journal of Botany* **68**, 635–650.

Nishida, H., Pigg, K. B., Kudo, K. & Rigby, J. F. (2007). New evidence of reproductive organs of *Glossopteris* based on permineralized fossils from Queensland, Australia. I. Ovulate organ *Homevaleia*. *Journal of Plant Research* **120**, 539–549.

Oliver, F. W. & Scott, D. H. (1904). On the structure of the Palaeozoic seed *Lagenostoma lomaxi*, with a statement of the evidence upon which it is referred to *Lyginodendron*. *Philosophical Transactions of the Royal Society* *of London B* **197**, 193–247.

Rothwell, G. W. (1971*a*). Additional observations on *Conostoma anglo-germanicum* and *C*. *oblongum* from the Lower Pennsylvanian of North America. *Palaeontographica B* **131**, 167–178.

Rothwell, G. W. (1981). The Callistophytales (Pteridospermopsida): reproductively sophisticated Paleozoic gymnosperms. *Review of Palaeobotany and Palynology* **32**, 103–121.

Rothwell, G. W. (1986). Classifying the earliest gymnosperms. In *Systematic and Taxonomic Approaches in Palaeobotany* (eds R. A. Spicer and B. A. Thomas), pp. 137–162. Systematics Association Special Volume No. 31, Clarendon Press, Oxford.

Rothwell, G. W. & Serbet, R. (1994). Lignophyte phylogeny and the evolution of spermatophytes: a numerical cladistic analysis. *Systematic Botany* **19**, 443–482.

Scotese, C. R. (2021). An atlas of Phanerozoic Paleogeographic maps: the seas come in and the seas go out. *Annual Review of Earth and Planetary Sciences* **49**, 669–718.

Scott, A. C., Hilton, J., Galtier, J. & Stampanoni, M. (2019). A charcoalified ovule adapted for wind dispersal and deterring herbivory from the Late Viséan (Carboniferous) of Scotland. *International Journal of Plant Science* **180**, 1059–1074.

Serlin, B. S. (1982). On the structure of *Cardiocarpus tritolopus*, a cordaite seed from the lower–middle Pennsylvanian of Kentucky. *Review of Palaeobotany and Palynology* **36**, 297–304.

Seyfullah, L. A., Hilton, J., Liang, M. & Wang, S. J. (2010). Resolving the systematic and phylogenetic position of isolated ovules: a case study on a new genus from the Permian of China. *Botanical Journal of the Linnean Society* **164**, 84–108.
